# Carbohydrate-Based NK1R Antagonists with Broad-Spectrum Anticancer Activity

**DOI:** 10.1021/acs.jmedchem.1c00793

**Published:** 2021-07-08

**Authors:** Rocío Recio, Patricia Lerena, Esther Pozo, José Manuel Calderón-Montaño, Estefanía Burgos-Morón, Miguel López-Lázaro, Victoria Valdivia, Manuel Pernia Leal, Bernard Mouillac, Juan Ángel Organero, Noureddine Khiar, Inmaculada Fernández

**Affiliations:** † Departamento de Química Orgánica y Farmacéutica, Facultad de Farmacia, Universidad de Sevilla, C/ Profesor García González, 2, 41012 Sevilla, Spain; ‡ Departamento de Farmacología, Facultad de Farmacia, Universidad de Sevilla, C/ Profesor García González, 2, 41012 Sevilla, Spain; § Institut de Génomique Fonctionnelle (IGF), INSERM, Université de Montpellier, CNRS, F-34094 Montpellier, France; ∥ Departamento de Química Física, Facultad de Ciencias Ambientales y Bioquímicas and INAMOL, Universidad de Castilla-La Mancha, Avenida Carlos III, s/n, 45071 Toledo, Spain; ⊥ Instituto de Investigaciones Químicas (IIQ), CSIC-Universidad de Sevilla, Avenida Américo Vespucio, 49, Isla de la Cartuja, 41092 Sevilla, Spain

## Abstract

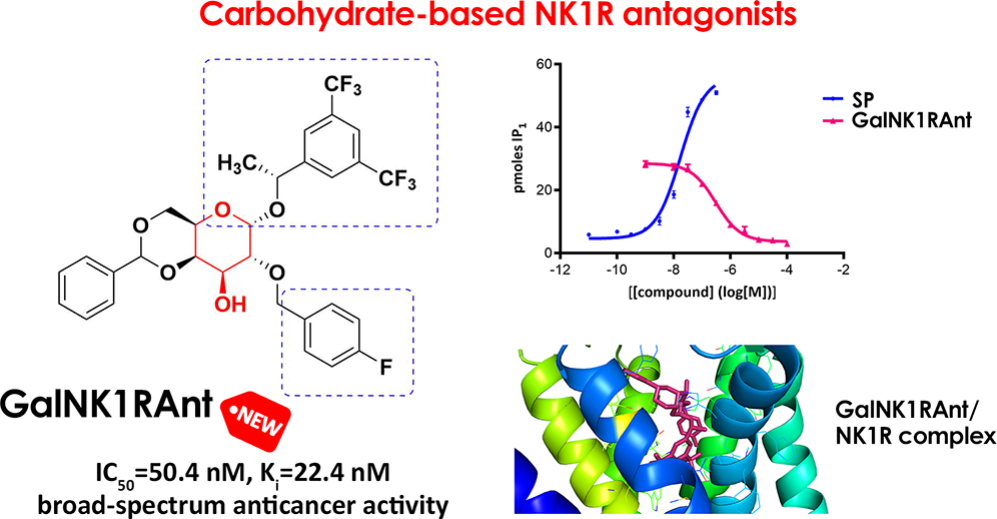

NK1R antagonists, investigated for the treatment of several pathologies, have shown encouraging results in the treatment of several cancers. In the present study, we report on the synthesis of carbohydrate-based NK1R antagonists and their evaluation as anticancer agents against a wide range of cancer cells. All of the prepared compounds, derived from either d-galactose or l-arabinose, have shown high affinity and NK1R antagonistic activity with a broad-spectrum anticancer activity and an important selectivity, comparable to Cisplatin. This strategy has allowed us to identify the galactosyl derivative **14α**, as an interesting hit exhibiting significant NK1R antagonist effect (*k*_inact_ 0.209 ± 0.103 μM) and high binding affinity for NK1R (IC_50_ = 50.4 nM, *K*_i_ = 22.4 nM by measuring the displacement of [^125^I] SP from NK1R). Interestingly, this galactosyl derivative has shown marked selective cytotoxic activity against 12 different types of cancer cell lines.

## Introduction

**Figure 1:**
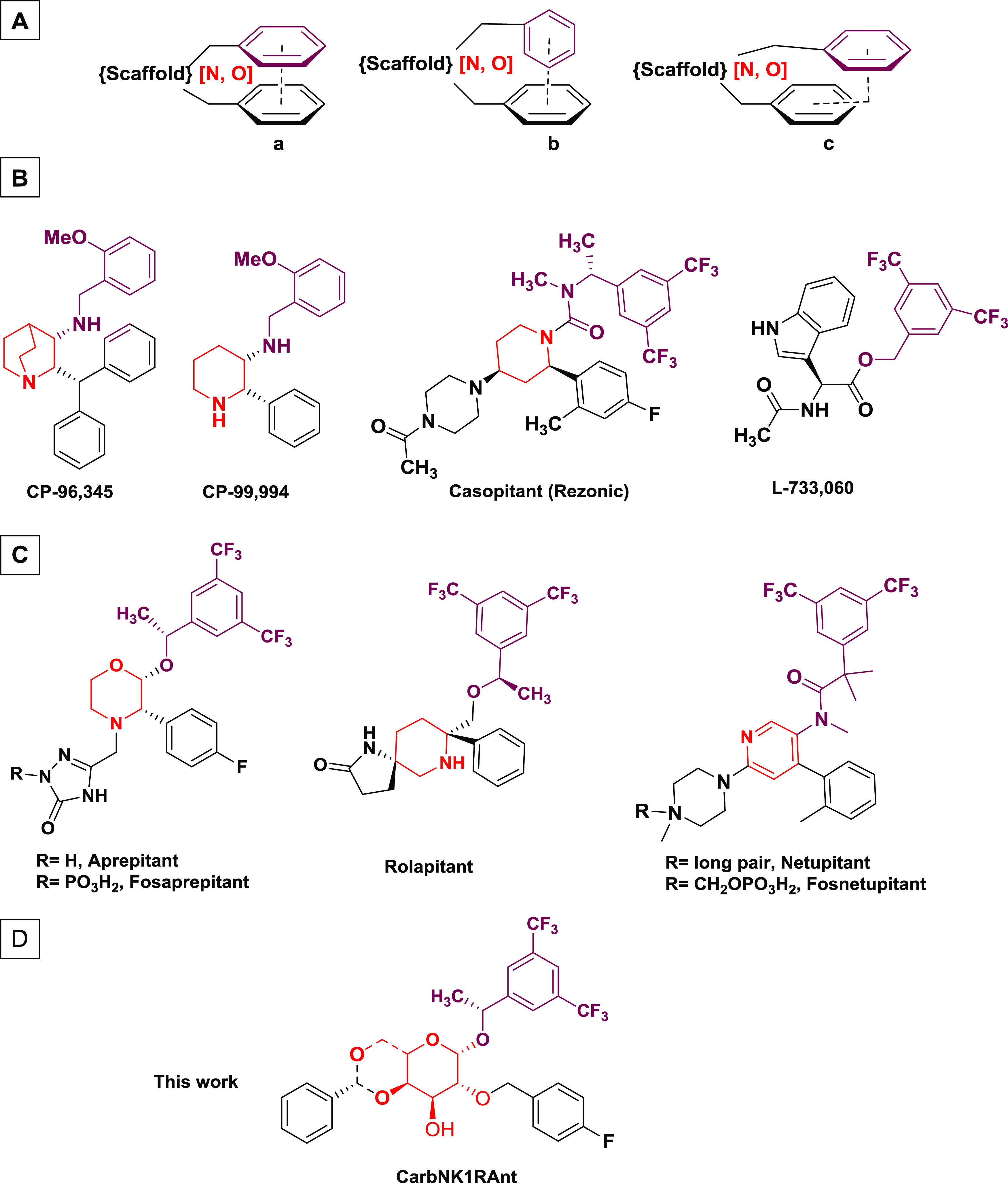
(A) Generalized nonpeptide NK1R antagonist pharmacophore consisting of two aromatic rings held together by various scaffolds, which contains at least one hydrogen-bond acceptor. Possible arrangement of the aromatic rings: parallel face-to-face (a), perpendicular “T” (b), and edge-on “L” (c). (B) Structures and color visualization of differences and similarities of some pioneering NK1R antagonists, (C) marketed NK1 antagonists, and (D) new NK1R antagonists derived from carbohydrates described in this work.

The NK1 receptor (NK1R), also known as tachykinin receptor 1 (TACR1),^1^ belongs to the superfamily of G-protein coupled receptors, which constitute ∼35% of the therapeutic targets of all of the pharmaceutical products on the market.^2^ The preferred endogenous agonist of NK1R is the undecapeptide substance P (SP), which acts as a neurotransmitter and neuromodulator.^3^ NK1R is present in the central and peripheral nervous systems, smooth muscle, endothelial cells, and also on cells that participate in immune response.^4^ Over the past four decades, intensive research has linked the SP-NK1R system to broad pathophysiological processes including nausea,^5^ analgesia,^6^ inflammation,^7^ and depression.^8^ In addition, NK1R is overexpressed in several cancers,^9^ including melanoma,^10^ astrocytoma,^11^ pancreatic ductal carcinomas,^12^ bone marrow,^13^ and gastric cancer,^14^ highlighting the potential therapeutic value of NK1R antagonists. This potential has recently been accentuated following several studies demonstrating the beneficial effect of NK1R antagonists on the health of patients infected with the SARS-CoV-2 virus, responsible for the current COVID19 pandemic.^15^ This perspective has boosted the search not only in academia but also in industry, with almost all important pharmaceutical companies investing in this field of selective and potent NK1R antagonists.^16^ A turning point in this race was the discovery of the first nonpeptide NK1R antagonist CP-96,345,^17^ which has been instrumental in the development of a number of antagonists with improved pharmacological properties; Figure 1.^16,18^ Structural optimizations around the central skeleton ultimately led to the development of Aprepitant,^19^ which became the first oral drug approved to enter the clinic, specifically targeting NK1R for the treatment of chemotherapy-induced nausea and vomiting.^20^ During the last 5 years, two other molecules, namely, Netupitant and Rolapitant, have been approved for clinical use for the same indication.^21^ It is worth mentioning that the discovery of effective NK1R antagonists is challenging due to the complexity of the NK1 transmembrane receptor, the crystal structure of which has only recently been determined.^22^ In a project directed toward the asymmetric synthesis of new NK1R antagonists, we have recently reported the asymmetric synthesis of 5-arylsulfinyl-2-amino-4*H*-pyrans and their application as antitumoral compounds.^23^ In the present work, we report on the stereoselective synthesis of carbohydrate-based NK1R antagonists (CarbNK1RAnt) and the determination of their selective cytotoxic activities against different types of cancer cell lines.^24^

## Results and Discussion

### Chemistry

**Figure 2:**
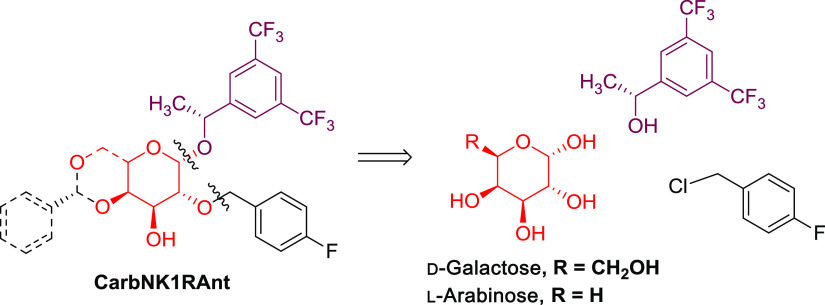
General structure and retrosynthetic route of carbohydrate-based NK1R antagonists.

Preliminary structure–activity relationship studies carried out on a large number of NK1R antagonists developed after the discovery of CP-96,345 allowed the proposition of a pharmacophore model, which consists of a heterocyclic scaffold substituted with at least two aromatic rings in a cis orientation; Figure 1A.^25^ In most cases, the fixed orientation between the two aromatic groups aforementioned can be a parallel face-to-face (Figure 1A(a)),^26^ a perpendicular T (Figure 1A(b)), or an edge-on L arrangement (Figure 1A(c)).^27^ The scaffolds used have evolved from the quinuclidine in CP-96,345, to a simpler piperidine in CP-99,994, Casopitant and Ralopitant; a morpholine ring in Aprepitant; a pyridine ring in Netupitant; or a simple acyclic chain in L-733,060; Figure 1B,C. The use of a heterocyclic saturated scaffold with two substituted carbons implies that the molecule is chiral with at least two stereogenic centers. Indeed, both Aprepitant and Ralopitant have three chiral centers, and of the eight possible diastereoisomers, only one has the desired activity. Accessing the desired compound as a single enantiomer is challenging, time consuming, and highly expensive. As an illustrative example, the Merck process developed for the synthesis of Aprepitant consists of a catalytic asymmetric (transfer) hydrogenation process coupled with a successful crystallization-induced diastereoselective transformation and a diastereoselective imine hydrogenation for the creation of three stereocenters.^28^ A simple and economical alternative to access heterocyclic compounds with multiple chiral centers in close proximity is to use compounds belonging to the chiral pool. A family of compounds well suited for this comprises the carbohydrates, stereochemically rich compounds with hydroxyl groups in virtually all arrangements, allowing the easy tuning of their steric, electronic, and three-dimensional (3D) structures. Moreover, as abundant and renewable biomolecules, they are accessible on a large scale at low cost. Indeed, some monosaccharides are even less expensive than the most common solvents used in synthetic laboratories. As part of a large program aimed at the utilization of carbohydrates in the asymmetric synthesis of synthetically and pharmacologically relevant molecules as well as in the synthesis of biomaterials,^29^ we decided to develop new NK1R antagonists using sugars as raw materials. As functional groups, we planned to incorporate at the anomeric position the 3,5-bis(trifluoromethyl)benzyl fragment, present in many NK1R antagonists including all currently marketed ones. As a second aromatic fragment, we opted for a *p*-fluorophenyl function, present in Aprepitant, Fosaprepitant, and other selective NK1R antagonists, as *O*-benzyl ether at the C-2 position of the carbohydrate (Figure 2). When both aromatic groups are at the α-face of the pyranose ring, an intramolecular π–π stacking interaction can be established, which, as stated before, has been proposed to play a significant role in other active analogues profiled in structure–activity relationship studies.

#### Synthesis of d-Galactose-Derived NK1R Antagonists (GalNKR1Ant)

**Scheme 1:**
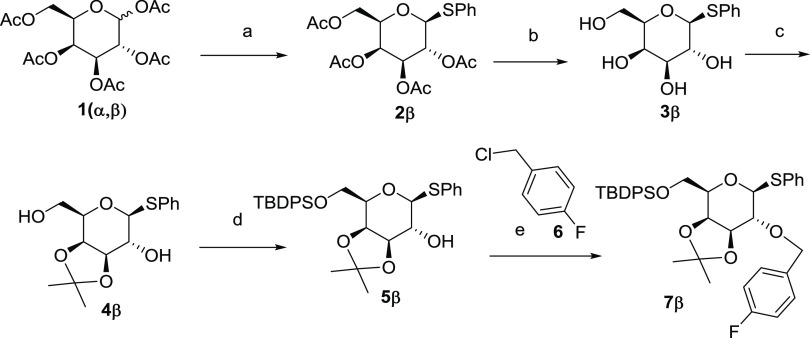
Synthesis of *p*-Fluorobenzyl d-Galactose Derivative **7β** Reagents and conditions: (a) PhSH, BF_3_·OEt_2_, CH_2_Cl_2_, 0 °C, 90%; (b) MeONa, MeOH, 0 °C, quant; (c) 2,2-dimethoxypropane (2,2-DMP), 10-camforsulfonic acid (CSA), rt, 85%; (d) *tert*-butyldiphenylsilyl chloride (TBDPSCI), imidazole, dimethylformamide (DMF), rt, 96%; (e) NaH, TBAI, (**6**), tetrahydrofuran (THF), rt, 90%.

Considering the planned sugar-based 1,2-cis substituted NK1R antagonist analogues, the choice of the starting carbohydrate as well as the sequence followed for implementing the substituents is crucial. From a synthetic point of view, it is a question of using the right sugar and a synthetic sequence that allows the selective functionalization of the C2-OH and to introduce the aglycon group in the carbohydrate α-face. Taking these considerations into account, among all of the hexopyranoses, d-galactose is the sugar of choice due to the different reactivity of its five hydroxyl groups, mainly due to the cis arrangement of C3-OH and C4-OH. Moreover, the α position of the aglyconic group requires the use of a nonparticipating group at C2 during the glycosylation step. Therefore, using commercially available d-galactose pentaacetate **1**(α,β), we first introduced the *p*-fluorophenyl group at position 2 of the pyranose ring as the corresponding *O*-benzyl ether, before introducing the (*R*)-1-[3,5-bis(trifluoromethyl) phenyl]ethan-1-ol at the anomeric position; Scheme 1.

Condensation of thiophenol with d-galactose pentaacetate **1(α,β)** in the presence of boron etherate trifluoride afforded the corresponding thioglycoside **2β** in high chemical yield (90%, Scheme 1), as a single anomer. A Zemplen deacetylation, followed by acid-catalyzed acetalation with 2,2-dimethoxypropane (DMP), afforded the 3,4-acetal **4β** in 85% yield. A regioselective silylation of the primary alcohol with *tert*-butyldiphenylsilyl chloride in DMF at rt afforded the mono hydroxylated derivative **5β** in high yield (96%). Finally, the installation of the *p*-fluorophenyl fragment was carried out in THF, using NaH as the base and *p*-fluorobenzyl chloride **6**. Thus, in only five high-yielding steps, the fully *O*-protected derivative **7β** was obtained on a multigram scale (Scheme 1).

**Scheme 2:**
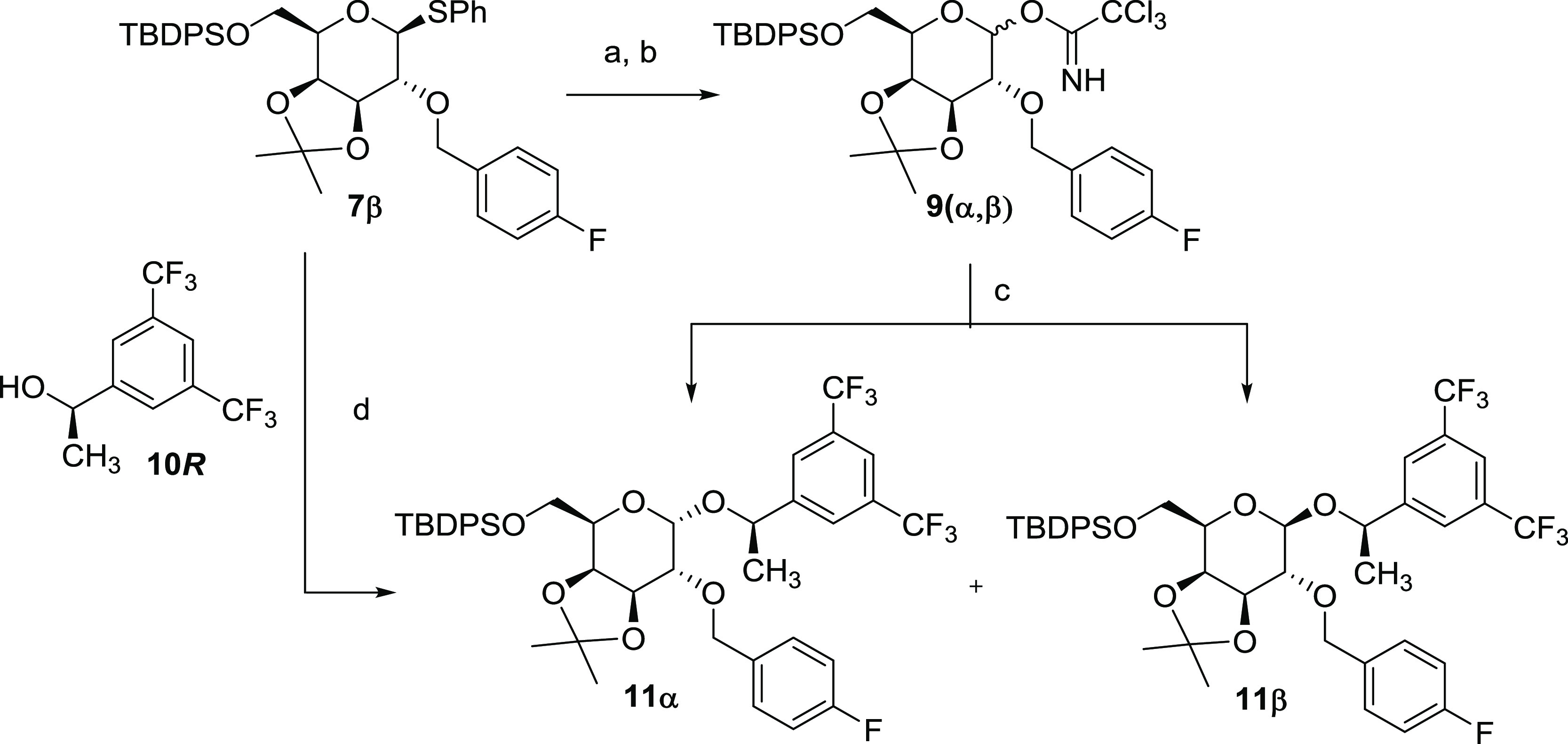
Syntheses of **11α** and **11β** by Glycosylation of *p*-Fluorobenzyl d-Galactose Derivative **7β** Reagents and conditions: (a) NBS, acetone (99%), darkness, −15 °C, **8α**:**8β** = 2:1, 91%; (b) CCl_3_CN, DBU (cat), c-hex:CH_2_Cl_2_ 4:1, rt, **9α**:**9β** = 3:1, 97%; (c) (*R*)-1-[3,5-bis(trifluoromethyl)phenyl]ethan-1-ol, **10*****R***, TMSOTf, diethyl ether, 4 Å MS, 0 °C to rt, column chromatography: **11α** 60% and **11β** 25%; (d) (*R*)-1-[3,5-bis(trifluoromethyl)phenyl]ethan-1-ol, **10*****R***, NIS, TMSOTf, CH_2_Cl_2_, 4 Å MS, 0 °C to rt, **11α**:**11β** = 1:1 (81%).

The introduction of the (*R*)-1-[3,5-bis(trifluoromethyl)phenyl]ethan-1-ol fragment in the anomeric position was accomplished as indicated in Scheme 2, following two different approaches. The first one, based on the use of the well-established trichloroacetimidate glycosylation reaction, consisted of treating **7β** with *N*-bromosuccinimide (NBS) in wet acetone and subsequent base-catalyzed addition of the obtained lactol **8(α**,**β)** to trichloroacetonitrile, to give the trichloroacetimidate donor as a 3:1 mixture of both anomers **9α** and **9β** (Scheme 2). Lewis-acid-catalyzed glycosylation of the chiral alcohol acceptor, (*R*)-1-[3,5-bis(trifluoromethyl)phenyl]ethan-1-ol **10*****R***, with the mixture of the trichloroacetimidate donors gave the fully protected *O*-glycosyl derivative **11(α,β)**, as a 3:1 mixture of both diastereomers, which were easily separated by column chromatography to give the **11α** and **11β** anomers, in 60 and 25% chemical yields, respectively. As an alternative route, the alcohol **10*****R*** was directly *O*-glycosylated, using thioglycoside **7β** as the glycosyl donor. For this, the mixture NIS/trimethylsilyl trifluoromethanesulfonate was used as an activator in the presence of MS (4 Å), affording an equimolecular mixture of both anomers, **11(α**,**β**), in a high 81% chemical yield.

**Scheme 3:**
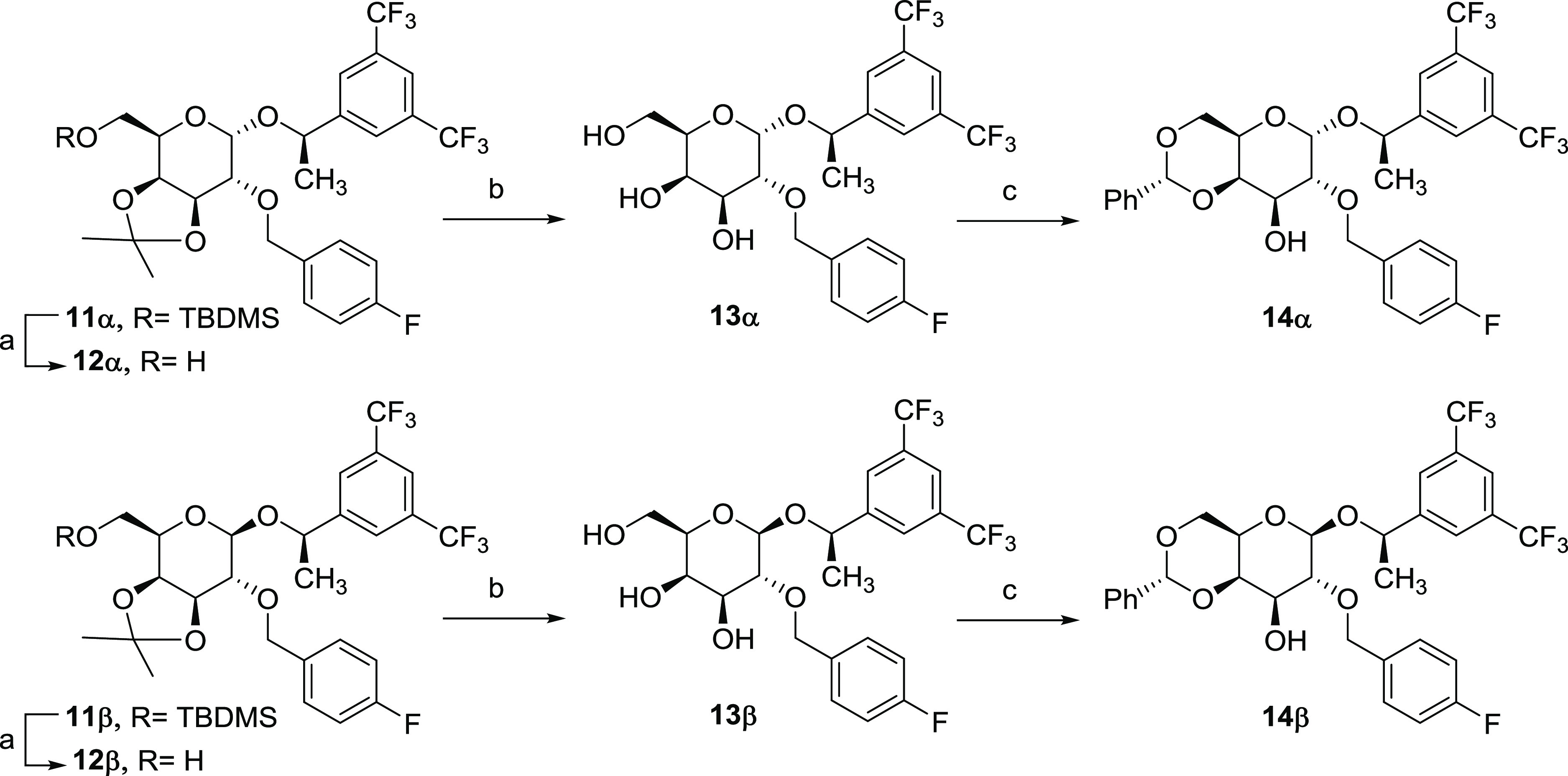
Deprotection and Protection of Hydroxylic Groups at **3**, **4**, and **6** Positions of d-Galactose Derivatives Reagents and conditions: (a) TBAF, THF, rt, 80% **12α**, and 75% **12β**; (b) CSA (cat), MeOH, rt, quant **13α** and **13β**; (c) benzaldehyde dimethyl acetal, CSA (cat), DMF, 40 °C, 95% **14α** and **14β**.

Considering the rigid conformations of the new d-galactoderivatives, as a fused bicyclic compound with a pyranose ring and a cyclic acetal, we were interested in studying the difference in bioactivity between both anomers, despite the fact that in the case of most NK1R antagonist analogues the cis isomer is the most active one. Moreover, in both diastereomers, **11α** and **11β**, deprotection of the hydroxylic groups at 3, 4, and 6 positions and modification of the protecting groups, as indicated in Scheme 3, give us the opportunity to modulate the lipophilicity of the carbohydrate derivatives and study, at the same time, the structure–activity relationship. Desilylation of the *O*-silyl ether in position 6 with TBAF, followed by acid hydrolysis of 3,4-dimethyl acetal and subsequent formation of the 4,6-*O*-benzylidene acetal, allows us to obtain the corresponding monoalcohols (**12** and **14**) or the more hydrophilic trihydroxylated analogues (**13**), with α or β configurations, starting from **11α** and **11β**, respectively (Scheme 3). The presence of the phenyl ring in the benzylidene moiety of **14α** and **14β** favors their hydrophobic and/or π–π stabilizing interactions with some NK1R amino acids, as we have determined by docking studies (vide infra).

Upon regioselective protection of the trihydroxylated epimers **13** (Scheme 3), a single benzylidene acetal diastereoisomer was formed. Although expected, we, however, conducted selective NOESY1D experiments to confirm the stereochemical outcome of the process.^30^ For both **14α** and **14β**, the registered NOESY1D spectra with selective excitation of the benzylidene acetal protons (see the Supporting Information) display three sets of signals, for an aromatic proton, H4, and H6 of the sugar. While the NOE contacts observed with the aromatic proton and H6 may be seen for the two diastereomers, the NOE contact observed with H4 is clearly indicative that the absolute configuration of the benzylidene acetal carbon center is indeed *R*.

#### Synthesis of l-Arabinose-Derived NK1R Antagonists (AraNK1Ant)

**Scheme 4:**
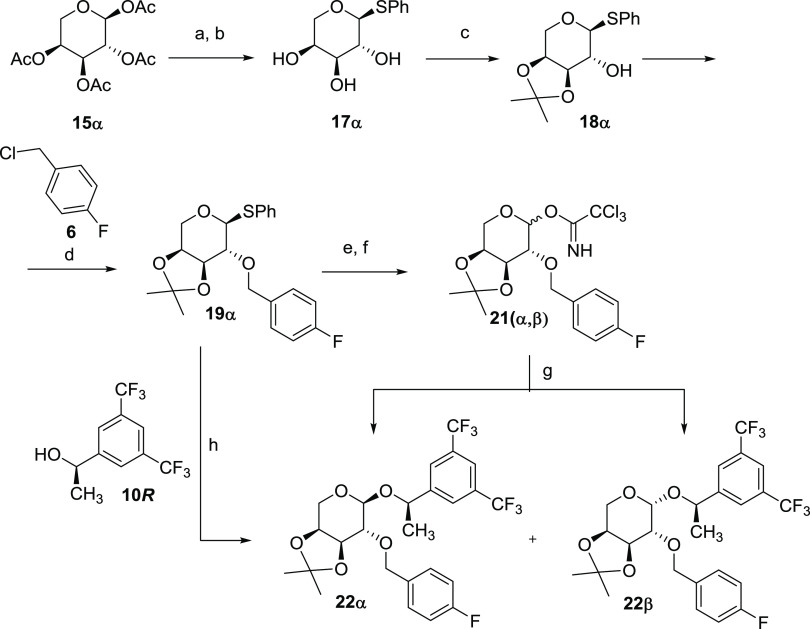
Synthesis of the Fully Protected l-Arabinose Derivatives **22α** and **22β** Reagents and conditions: (a) PhSH, BF_3_·OEt_2_, CH_2_Cl_2_, 0 °C, quant; (b) MeONa, MeOH, 0 °C to rt, quant; (c) 2,2-DMP, CSA, rt, 89%; (d) NaH, TBAI, **6**, THF, rt, quant; (e) NBS, acetone (99%), darkness, −15 °C, **20α**:**20β** = 1:2, 80%; (f) CCl_3_CN, DBU (cat), CH_2_Cl_2_, rt, **21α**:**21β** = 1:2, quant; (g) (*R*)-1-[3,5-bis(trifluoromethyl)phenyl]ethan-1-ol (**10*****R***), Et_2_O, 4 Å MS, 0 °C to rt, column chromatography: **22α** 16% and **22β** 50%; (h) (*R*)-1-[3,5-bis(trifluoromethyl) phenyl]ethan-1-ol (**10*****R***), NIS, CH_2_Cl_2_, 4 Å MS, 0 °C to rt, **22α**:**22β** = 1:1 (66%).

The pentapyranose l-arabinose is structurally related to d-galactose with the sole, and important, difference of lacking the 6-hydroxymethyl group. Consequently, starting from l-arabinose tetraacetate **15α**, a synthetic approach similar to that developed for d-galactose has allowed us to obtain a series of analogues in only five or six steps and in the multigram scale; Scheme 4. Additionally, the absence of the 6-hydroxymethyl group in the obtained analogues (Figure 2) will affect both their lipophilicity and conformational behavior.

**Scheme 5:**
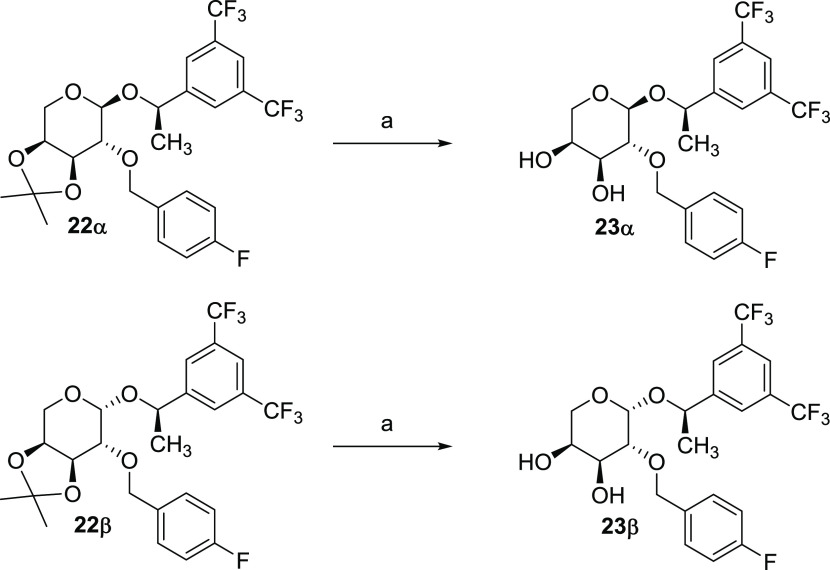
Syntheses of the Dihydroxylic l-Arabinose Derivatives **23α** and **23β** Reagents and conditions: (a) CSA (cat), MeOH, rt, quant.

Thus, the 2-*O*-*p*-fluorobenzyl derivative **19α** was obtained as a single diastereomer, starting from per-*O*-acetylated α-l-arabinose **15α**, in only four high-yielding steps, with a 77% overall yield; Scheme 4. The chiral alcohol **10*****R*** was introduced in the anomeric position using the trichloroacetimidate method to give a 1:2 mixture of both anomeric *O*-glycosyl derivatives **22α** and **22β**. They were also obtained directly from the phenylthioglycoside **19α**, as a 1:1 mixture of anomers by activation with trifluoromethylsilyl triflate as the Lewis acid, in the presence of NIS and 4 Å MS, at 0 °C. The two diastereoisomers **22α** and **22β** showed a very different separation factor, which, after column chromatography, allowed them to be obtained in the pure form with an overall 66% yield. Finally, acid hydrolysis of the 3,4-dimethyl acetal using CSA in methanol yielded the corresponding dihydroxy derivatives **23α** and **23β**, in quantitative yields; Scheme 5.

### Anticancer Activity

First, we evaluated the cytotoxicity of our carbohydrate derivatives against MRC-5 human nonmalignant lung cells and A549 human lung cancer cells using the 3-(4,5-dimethylthiazol-2-yl)-2,5-diphenyltetrazolium bromide (MTT) assay under the same experimental conditions. Both cell lines were exposed to different concentrations of compounds **12α**, **12β**, **13α**, **13β**, **14α**, **14β**, **22α**, **22β**, **23α**, and **23β** during 48 h before quantifying the cell viability. Aprepitant was used as a reference control to assess the activity of the new derivatives with a known NK1R antagonist, and Cisplatin, a well-known anticancer standard drug, was used as a positive control to study the possible selective cytotoxicity. The obtained results are collected in Table 1 (see Figures S2 and S3). All of the carbohydrate derivatives showed some selective cytotoxicity against the cancer cell line, i.e., for a given concentration, A549 cancer cells were more sensitive than MRC-5 normal cells. **12α**, **12β**, and Aprepitant showed the lowest selective activity, with selectivity index values ∼1.5 (Table 1). **13α**, **13β**, **23α**, and **23β** showed a modest selectivity, with IC_50_ values in A549 ∼2-fold lower than in MRC-5. Interestingly, both α and β diastereomers of **14** and **22** showed the highest selective cytotoxicity against cancer cells, even higher than that of the anticancer drug Cisplatin. **14α** and **22β** were the most selective compounds. A549 cancer cells were over 10 times more sensitive than MRC-5 cells to these derivatives.

**Table 1:** IC_50_ Values of Carbohydrate Derivatives and Cisplatin on Lung Cancer Cells (A549) versus Lung Normal Cells (MRC-5)

	IC_50_ (mean ± SEM; μM)		
compound	MRC-5 (normal)	A549 (cancer)	selectivity index^a^
**12α**	27.8 ± 5.7	18.7 ± 0.2	1.5 ± 0.3
**12β**	39.7 ± 4.2	23.4 ± 3.8	1.7 ± 0.2
**13α**	92.23 ± 16.7	40.9 ± 3.1	2.3 ± 0.4
**13β**	155.7 ± 29.6	67.5 ± 18.2	2.5 ± 0.3
**14α**	**225.8 ± 100.9**	**24.2** ± **7.8**	**20.1** ± 9.4
**14β**	130.8 ± 10.7	29.7 ± 5.1	5.8 ± 1.0
**22α**	503.7 ± 48.7	171.9 ± 47.3	3.9 ± 1.0
**22β**	**>800**	**59.5** ± **11.4**	**>10.7**
**23α**	50.4 ± 3.3	20.8 ± 4.0	2.8 ± 0.7
**23β**	56.2 ± 2.4	31.3 ± 5.6	2.1 ± 0.4
**Aprepitant**	28.9 ± 6.8	18.3 ± 3.4	1.5 ± 0.1
**Cisplatin**	99.2 ± 37.0	13.5 ± 2.7	8.6 ± 3.9

a The selectivity index is the mean of the selectivity indices calculated in each individual experiment. The selectivity index is calculated by dividing the IC_50_ value obtained in the nonmalignant cell line (MRC-5) by that in the cancer cell line (A549). The most selective compounds are shown in bold.

It should be noted that, in general, the carbohydrate derivatives showed higher selectivity than Aprepitant. d-Galactosyl derivatives were the most active compounds, with similar activity to Aprepitant and Cisplatin against A549 cancer cells. Deprotection of the hydroxyl groups at 3, 4, and 6 positions increased the selective activity in **13α** and **13β**, but it also decreased their cytotoxic activity. The introduction of the 4,6-*O*-benzylidene acetal in diastereomers **14** increased the selective activity without compromising the cytotoxicity against cancer cells. Indeed, **14α** showed similar cytotoxicity as Cisplatin against A549 cancer cells, being less cytotoxic against MRC-5 normal cells. l-Arabinose derivatives **22α** and **22β** also showed high selective anticancer activity; however, they were less cytotoxic than the galactose derivatives **14** against cancer cells. **14α** was ∼2.5-times more cytotoxic against A549 than **22β**, being both the most selective anticancer derivatives. For that reason, **14α** was selected to delve into its anticancer activity.

**Figure 3:**
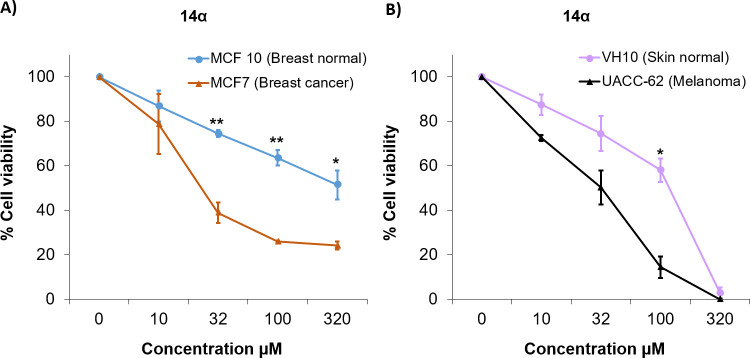
Compound **14α** induces selective cytotoxicity toward breast cancer and melanoma cells. Breast cell lines (A) and skin cell lines (B) were exposed to the NK1R antagonist **14α** for 48 h. Cell viability was estimated with the MTT assay. Values (percentage of cell viability) derived from two independent experiments performed in duplicate, mean with SEM, and *p* values (paired *t*-test) are indicated.

Next, we used MCF7 breast cancer cells, MCF 10 normal breast epithelial cells, UACC-62 melanoma cells, and VH10 skin nonmalignant cells to explore whether the new carbohydrate derivatives were also selective against other types of cancer. These cells were exposed to several concentrations of the compound **14α** for 48 h, and cell viability was measured by the MTT assay (Figure 3). **14α** was ∼10 times more cytotoxic against MCF7 breast cancer cells than against MCF 10 normal cells. The IC_50_ values (mean ± standard error of mean (SEM); μM) in MCF7 and MCF 10 cells were, respectively, 23.9 ± 5.0 and 291.3 ± 52.6. **14α** also showed selective cytotoxic activity against melanoma cells. UACC-62 melanoma cells were 4.3 times more sensitive than VH10 skin normal cells to the cytotoxic effect of **14α**. The IC_50_ values (mean ± SEM; μM) in UACC-62 and VH10 were, respectively, 31.9 ± 6.0 and 117.9 ± 12.3. It is worth mentioning that **14α** showed similar cytotoxicity against the three cancer cell lines, with IC_50_ values between 25 and 30 μM.

As **14α** displayed cytotoxicity against three cancer cell lines (lung cancer, breast cancer, and melanoma), we decided to assess whether **14α** was also active against other types of cancer. Thirteen human cancer cells lines and one human nonmalignant cell line were treated with **14α** for 96 h. We used longer exposure times because some of these cell lines have long cell cycles (24–48 h); this longer exposure time allows the cells to pass several cycles in the presence of **14α**. Cell viability was estimated with the resazurin assay. HaCaT human skin nonmalignant cells were used to study selectivity; these cells are derived from normal adult tissue and have a division rate similar to that of cancer cells. One key limitation of most anticancer drugs is that, in addition to targeting cancer cells, they also target nonmalignant cells with similar division rates. Indeed, results in Table 2 show that our positive control, Cisplatin, did not spare normal HaCaT cells from its cytotoxicity, with **14α** being more selective than Cisplatin. Compound **14α** showed a modest selectivity against HT29 (colorectal cancer) and A64-CLS (submaxillary gland adenoma) cells and a very low selectivity against T24 (bladder cancer) and MDA-MB-231 (triple-negative breast cancer). Interestingly, **14α** showed a marked selective cytotoxic activity against GAMG (glioblastoma), HNO97 (tongue cancer), MeWo (melanoma), PC-3 (prostate), Sk-Br-3 (HER2-positive breast cancer), An3Ca (endometrial cancer), and Sk-OV-3 (ovarian cancer). These cell lines were at least 3 times more sensitive than the normal cell line to **14α** treatment. HepG2 (hepatocarcinoma) and KATO III (gastric cancer) were at least 9-fold more vulnerable to **14α** than HaCaT nonmalignant cells. These data show that **14α** induces selective anticancer activity against a variety of cancer cell lines and suggest that our d-galactose derivative has anticancer potential. The key role of NK1R in cell proliferation and the elevated expression of NK1R identified in several cancer types^31^ could explain the higher sensitivity of cancer cell lines (A549, MCF7, UACC-62, GAMG, HNO97, MeWO, PC-3, HepG2, KATO III) than normal cell lines (MRC-5, MCF 10, VH10, HaCat) to compound **14α**.

**Table 2:** IC_50_ Values of **14α** and Cisplatin on a Panel of Human Cell Lines^a^

	14α		Cisplatin	
cell line	IC_50_ (mean ± SEM; μM)	selectivity index (vsHaCaT: mean ± SEM)^b^	IC_50_ (mean ± SEM; μM)	selectivity index (vsHaCaT; mean ± SEM)^b^
HaCaT (human skin normal)	596.6 ± 8.7		2.1 ± 0.7	
GAMG (glioblastoma)	183.8 ± 1.9	3.2 ± 0.0	3.2 ± 0.9	0.7 ± 0.0
HNO97 (tongue cancer)	175.1 ± 73.9	4.1 ± 1.7	2.5 ± 0.4	0.8 ± 0.1
A64-CLS (submaxillary gland adenoma)	236.5 ± 15.7	2.5 ± 0.2	4.5 ± 1.2	0.5 ± 0.0
MeWo (melanoma; BRAF WT)	112.9 ± 16.1	5.4 ± 0.8	2.2 ± 1.1	1.0 ± 0.1
T24 (bladder cancer)	490.8 ± 249.5	1.7 ± 0.8	1.6 ± 0.5	1.3 ± 0.0
PC-3 (prostate cancer)	149.4 ± 44.0	4.4 ± 1.2	3.1 ± 0.2	0.7 ± 0.2
Sk-Br-3 (HER2-positive breast cancer)	192.4 ± 14.8	3.1 ± 0.2	4.3 ± 0.9	0.5 ± 0.1
MDA-MB-231 (triple-negative breast cancer)	332.2 ± 32.8	1.8 ± 0.2	10.2 ± 6.3	0.3 ± 0.1
AN3Ca (endometrial adenocarcinoma)	137.9 ± 6.4	4.3 ± 0.1	1.8 ± 1.4	2.1 ± 1.2
Sk-OV-3 (ovarian cancer)	153.1 ± 28.4	4.0 ± 0.8	4.3 ± 0.5	0.5 ± 0.2
KATO III (gastric cancer)	28.8 ± 11.7	24.7 ± 9.8	1.8 ± 0.2	1.2 ± 0.5
HepG2 (hepatocarcinoma)	133.3 ± 95.9	9.4 ± 6.8	1.8 ± 0.2	1.2 ± 0.2
HT29 (colorectal cancer)	256.0 ± 107.9	2.9 ± 1.2	4.9 ± 0.8	0.4 ± 0.1

a Cells were treated for 96 h, and cell viability was determined by the Resazurin assay.

b The selectivity index is the mean of the selectivity indices calculated in each individual experiment. The selectivity index is calculated by dividing the IC_50_ value obtained in the nonmalignant cell line (HaCaT) by that in the cancer cell line.

### Study of the Mechanisms of Anticancer Activity

Our next aim was to study the possible mechanisms involved in the selective anticancer activity of **14α**. Because tumor cells rely on glycolysis for survival more than normal cells,^32^ we evaluated if our compound behaved as a glycolysis inhibitor. Since glycolysis consumes glucose and produces lactate, we measured the concentrations of glucose and lactate in untreated A549 cells and in cells exposed for 8 h to **14α** and to the known glycolysis inhibitor dichloroacetate. Unlike dichloroacetate, **14α** did not reduce glucose consumption or lactate production, therefore indicating that **14α** does not inhibit glycolysis.

It is known that cancer cells have higher basal levels of reactive oxygen species (ROS) than normal cells, making them more sensitive to exogenous induction of ROS.^33^ Several studies have shown that Aprepitant and other NK1R antagonists as SR140333 increased mitochondrial ROS production, leading to apoptosis.^34^ Therefore, we decided to study whether the generation of ROS was involved in the selective cytotoxic effect of **14α**. A549 cancer cells were treated with **14α** for 48 h in the presence or absence of three antioxidants: *N*-acetylcysteine (antioxidant activity through the glutathione system), catalase (hydrogen peroxide-degrading enzyme), and Mn(III) tetrakis(1-methyl-4-pyridyl)porphyrin pentachloride (MnTMPyP; superoxide anion scavenger). None of these three antioxidants reduced the cytotoxicity of **14α** on A549 cells. These results suggest that the anticancer effect of **14α** is not mediated by ROS production.

Several anticancer drugs (e.g., antimetabolites) inhibit DNA synthesis, block replication fork progression, and generate DNA damage that ultimately leads to cell death. In normal cells, this type of DNA damage is usually repaired by homologous recombination (HR).^35^ However, some cancers are HR-deficient and, therefore, they are more sensitive to these drugs.^36^ Because data suggest that NK1R blockade decreases the synthesis of DNA through the MAPK pathway,^31a,37^ we tested whether HR-deficient cells were more sensitive to **14α**. The HR-deficient VC8 cell line (V79 Chinese hamster lung cells mutated in BRCA2) and the HR-proficient VC8B2 cell line (VC8 cells complemented with human BRCA2) were treated with **14α** for 24 h. After a recovery period of 48 h, cell viability was estimated with the MTT assay. The HR-deficient VC8 cells were slightly more sensitive to **14α** than the HR-proficient VC8B2 cells. IC_50_ values (mean ± SEM; μM) in VC8 and VC8B2 were, respectively, 36.5 ± 4.9 and 63.7 ± 11.1. These data suggest that the possible generation of DNA damage by **14α**, which could be repaired by HR, might play a minor role in the cytotoxicity of this compound.

### Evaluation of the NK1R Antagonist Effect and Correlation with the Anticancer Effect of Selected Compounds

Finally, tests were carried out to, first, confirm the NK1R antagonist activity of the new carbohydrate derivatives and, then, its correlation with the observed anticancer effect. For this, we have chosen three representative derivatives for their chemical structure and their antitumor activity including the d-galactosyl derivative **14α**, which provided the best effect, its trihydroxylated analogue **13α**, exhibiting a lower anticancer activity, and an arabinose derivative **23β**, exhibiting an intermediate activity.

The antagonist activity of these compounds was determined by their ability to inhibit NK1R using the IPone test. This test quantifies the inositol monophosphate (IP1) accumulated inside the cell by time homogeneous fluorescence (HTRF) technology. The accumulation of IP1 is an indicator of the activation of NK1R, so that the NK1R agonist ligands cause an increase in the levels of IP1 in the absence of SP while, on the contrary, the antagonist ligands produce a decrease of these levels in the presence of the endogenous ligand SP. As a control compound, we used the N-acetyl-l-typtophan 3,5(bis-trifluorometil)benzyl ester derivative (L732,138), whose NK1R antagonist activity is well-known at the molecular level.

**Figure 4:**
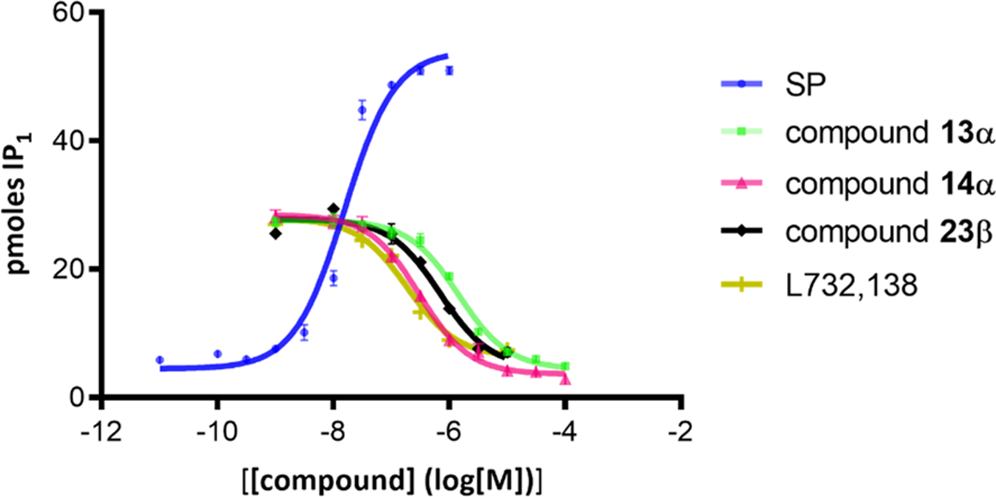
Concentration–response curves for the agonist and antagonist effects of SP (blue), L732,138 (olive green), α-d-galactosyl derivatives (**13α** green; **14α** pink), and β-d-arabinosyl derivative **23β** (black) using the IPone assay. The IP_1_ accumulation (pmoles) was measured as described in the Experimental Section. Data are illustrated from a representative experiment performed at least three times.

As shown in Figure 4, both d-galactosyl derivatives, **13α** and 1**4α**, as well as the arabinose derivative **23β**, exhibit significant inhibitory effect on the SP activity and can therefore be considered as NK1R antagonists. Specifically, the *k*_inact_ values for the galactosyl derivatives **13α** and 1**4α** are 0.651 ± 0.239 and 0.209 ± 0.103 μM, respectively, and 0.494 ± 0.047 μM for the arabinosyl derivative **23β**; Figure 4. Interestingly, by comparing the potency of the NK1R inhibitory activity of the three synthetic derivatives (Table 3, entries 2–4), as well as that known of Aprepitant (Table 3, entry 1), with their anticancer activity against the lung cancer cell line A549, determined previously, we note that there is a clear correlation between both activities; Table 3.

**Table 3:** NK1R Antagonist Effects and Anticancer Activities of **13α**, **14α**, and **23β** Derivatives and Aprepitant

		NK1R antagonist activity		anticancer activity (A549)
entry	compound	IC_50_ (mean ± SEM; μM)	*k*_inact_ (mean ± SEM; μM)	IC_50_ (mean ± SEM; μM)
**1**	**Aprepitant**^a^	1.41 × 10^–3^ a	0.023 × 10^–2^ a	18.3 ± 3.4
**2**	**13α**	1.100 ± 0.403	0.651 ± 0.239	40.9 ± 3.1
**3**	**14α**	0.353 ± 0.173	0.209 ± 0.103	24.2 ± 7.8
**4**	**23β**	0.833 ± 0.079	0.494 ± 0.047	31.3 ± 5.6

a Values from ref (38).

Indeed, Aprepitant (Table 3, entry 1) > **14α** (Table 3, entry 3) > **23β** (Table 3, entry 4) > **13α** (Table 3, entry 2) for both activities. Although the exact mechanism(s) of the antitumoral activity of NK1R antagonists is poorly understood, and is currently the subject of intense research,^39^ the results described indicate that the anticancer activity obtained with the carbohydrate analogues is mediated, at least in part, by the NK1 receptor.

Next, a binding affinity assay conducted by measuring the displacement of [^125^I]SP from the hNK1R from U-373MG cells^40^ reveals that **14α** has an excellent affinity for NK1R with an IC_50_ = 50.4 nM and *K*_i_ = 22.4 nM. The improved NK1R antagonist activity of **14α** over the trihydroxylated analogue **13α** is likely due to the presence of the benzylidene acetal moiety, which can establish additional stabilizing interactions with the NK1 receptor, as has been confirmed in modeling calculations.

### Docking Calculations

To gain knowledge about the interactions involved in the molecular recognition among ligands **13α**, **13β**, **14α**, **14β**, Aprepitant, and NK1R, we performed docking calculations. The obtained docking scores were −9.5, −9.6, −12.0, −12.4, and −11.2 kcal/mol for **13α**, **13β**, **14β**, **14α**, and Aprepitant, respectively. The capability of docking calculations has been widely recognized for precise predictions of the optimal ligand binding geometries as well as binding interactions.^41^ Thus, using the obtained docking poses and analyzing the hydrophobicity of the binding site, we characterize the intermolecular interactions between the studied ligands and NK1R.

**Figure 5:**
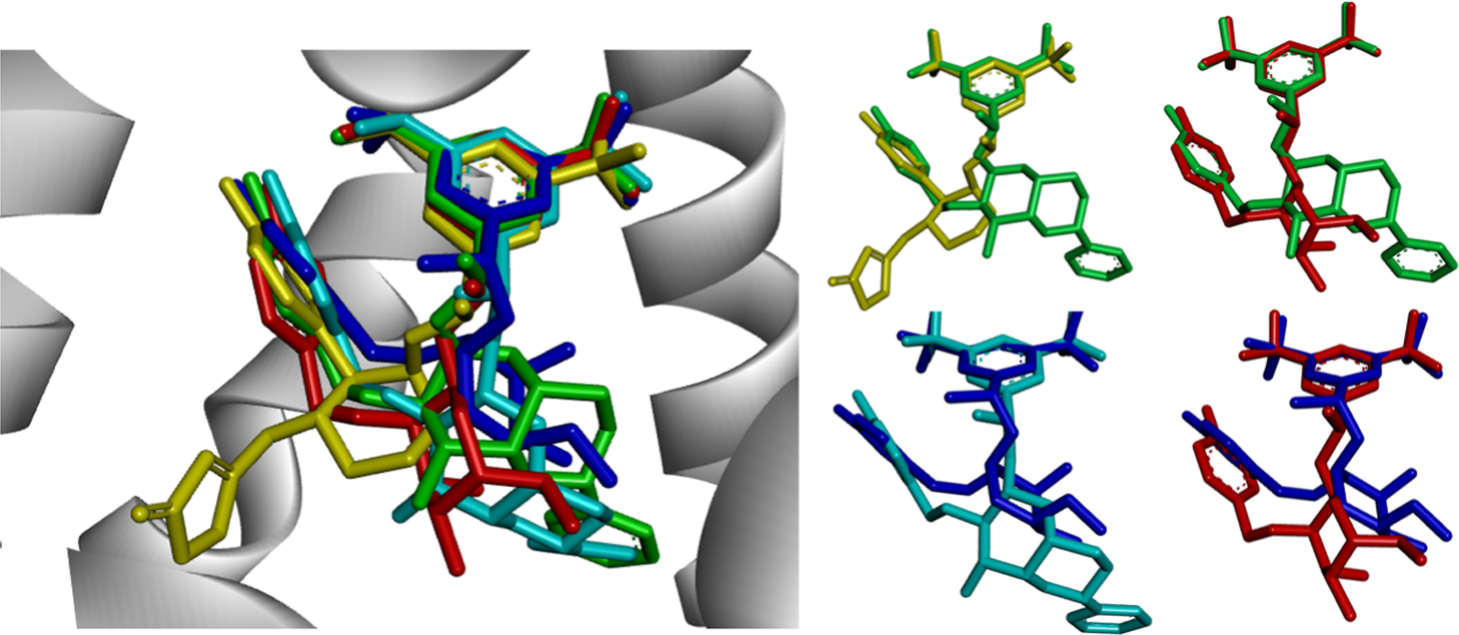
Superposition of the docked poses of Aprepitant (yellow), **13α** (red), **13β** (dark blue), **14α** (green), and **14β** (light blue) complexed with NK1R (PDB ID: 6HLO). For clarity, hydrogen atoms have been removed.

The binding site of NK1R has been previously described,^22b^ and it consists of a deep concave pocket with a large hydrophobic region that can maximize favorable protein–ligand contacts. For this reason, we used the octanol–water partition coefficient (or log *P*) of the different fragments of the ligands to obtain information about the interaction patterns of the ligands in the binding site.^42^ Docking results revealed that all of the carbohydrate derivatives show a noticeable spatial overlap of the hydrophobic fragments 3,5-bis(trifluoromethyl)phenyl and 4-fluorophenyl, with root-mean-square deviation (RMSD) values from 0.115 to 0.207 Å for the former and from 0.120 to 0.950 Å for the latter (Figure 5). The hydrophobicity surface of the binding pocket was performed because according to complementary ligand–protein binding interactions, strong hydrophobic regions of a binding site are usually occupied by hydrophobic fragments of ligands.

**Figure 6:**
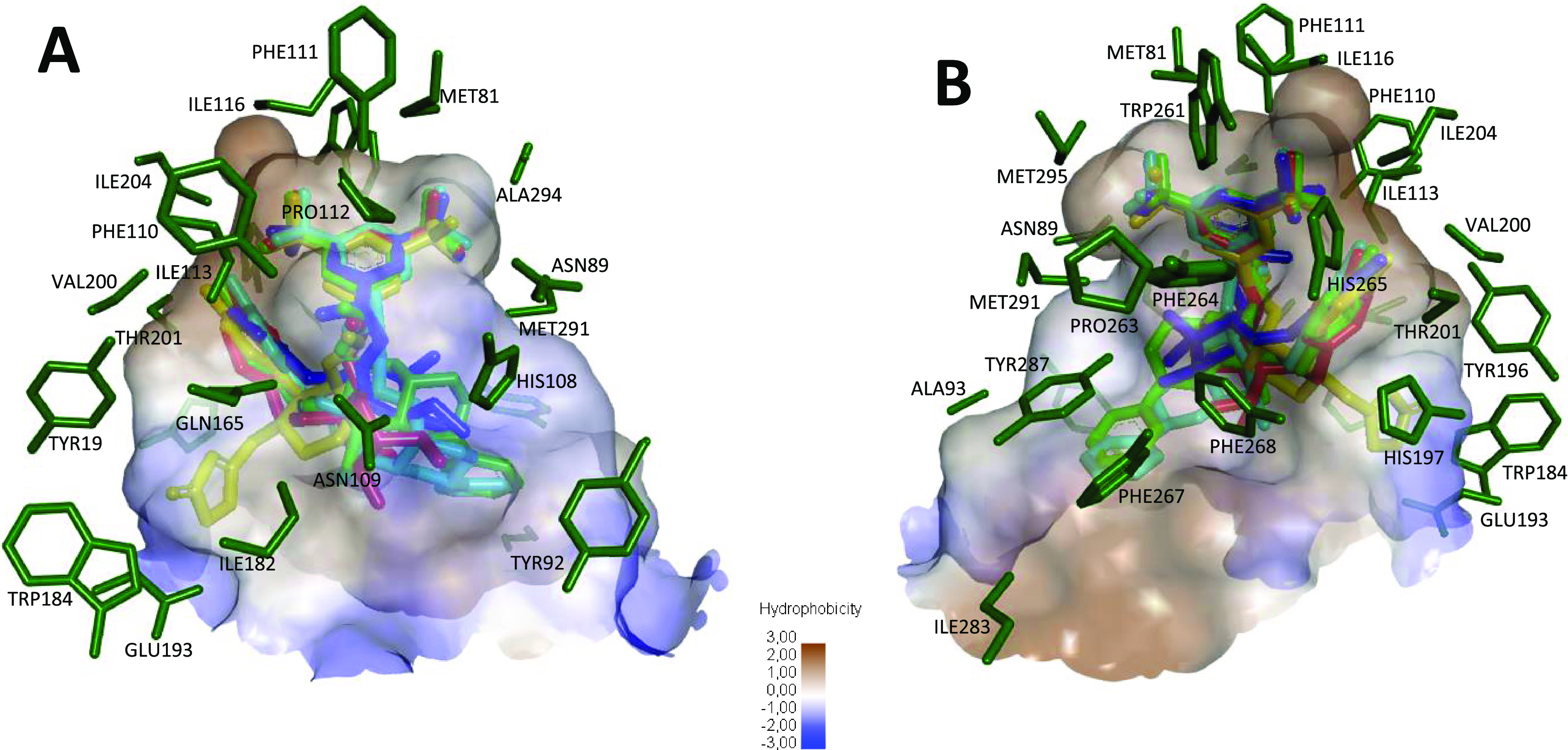
Binding site residues (dark green), hydrophobicity surface, and docked poses for Aprepitant (yellow), **14α** (green), **13α** (red), **13β** (dark blue), and **14β** (light blue), complexed with NK1R (PDB ID: 6HLO). Top (A) and down (B) views are on the left and right sides, respectively. Brown color represents the most hydrophobic surface area and blue color, the least hydrophobic.

Figure 6 reveals that most of the amino acids located at the bottom of the binding site have hydrophobic or amphipathic character (Ile113, Ile116, Ile204, Ala294, Met81, Met295, Met291, Phe110, Phe111, Phe264, Trp261, Tyr196). On the other hand, Figure 6A shows a top view of the middle-region binding site, which is dominated by charged/hydrophilic amino acids with large H-bonding donor/acceptor capacities (Gln165, His108, Asn109). On the contrary, Figure 6B indicates that hydrophobic amino acids are located at the down view of the middle region (Phe264 and Phe268). In addition to this, a strong hydrogen-bonding acceptor residue, Glu193, is found on the left side of the top view of the binding site outer region, which is accompanied by other amino acids with noticeable H-bonding donor/acceptor capacities (Trp184, Tyr196, Hys197). By contrast, the opposite side of this outer region is mainly surrounded by Ala93, Phe287, Ile283, and Tyr287 residues.

**Figure 7:**
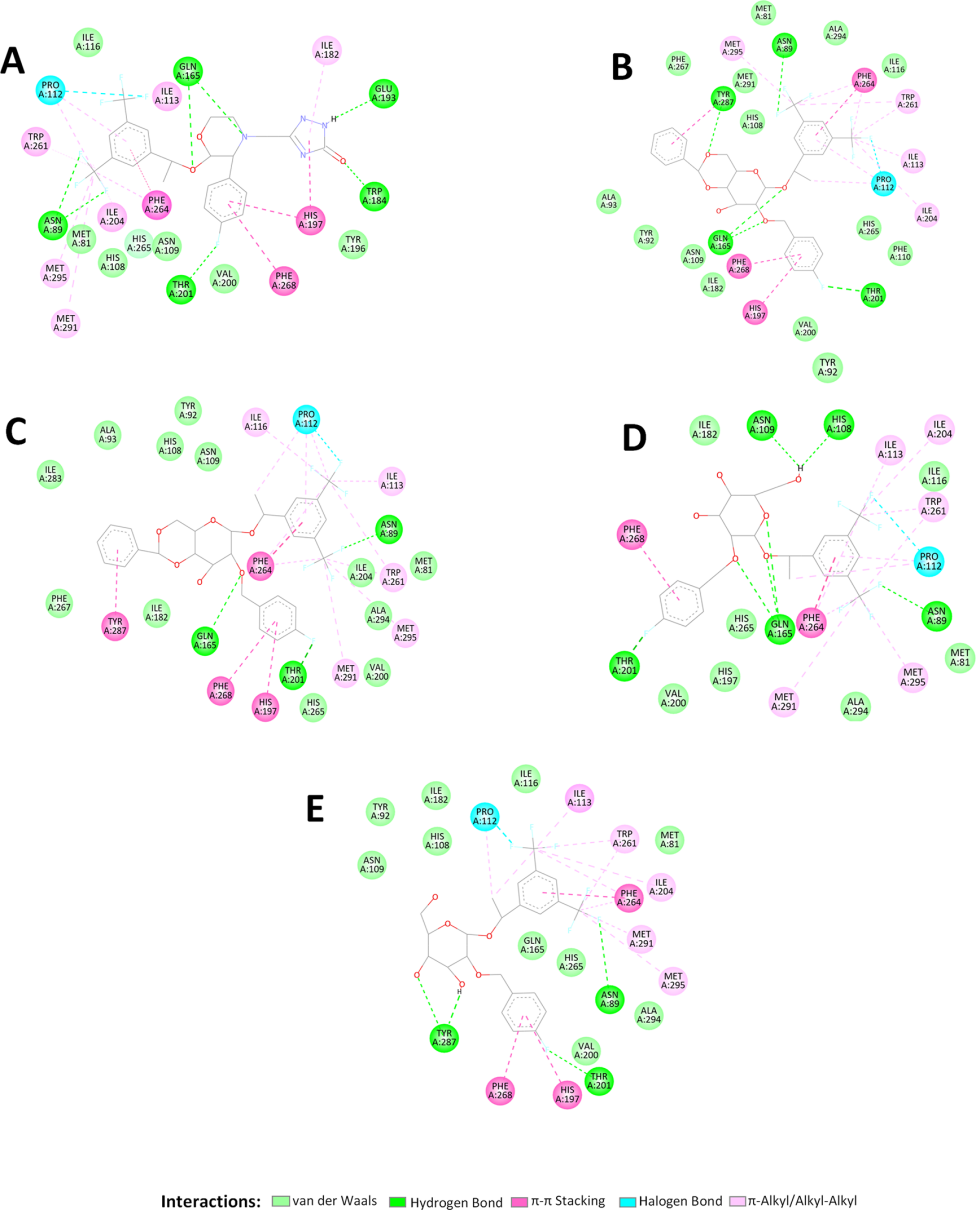
Two-dimensional view of the interaction type of Aprepitant (A), **14α** (B), **14β** (C), **13α** (D), and **13β** (E) with surrounding amino acids of NK1R (PDB ID: 6HLO).

The analysis of the ligand–protein interactions of the docked complexes (Figure 7) shows that the hydrophobic fluorinated aromatic fragments, 3,5-bis(trifluoromethyl)phenyl (log *P* = 3.70) and *p*-fluorophenyl (log *P* = 2.10), in all of the studied carbohydrate derivatives are stabilized at the bottom side of the binding site through a large number of hydrophobic interactions, π–π stacking interactions with Phe268, His197, and Phe264, π–alkyl interaction with Pro112, and alkyl–alkyl interactions with Met295, Phe264, Met291, Ile113, Ile204, and Trp261 (Figure 7). Besides, two types of electrostatically driven interactions were observed (values in parenthesis reflect the interaction distances range found): (i) weak hydrogen bonds between fluoromethyl fragments and amino groups of Asn89 (2.51–2.65 Å) and Thr201 (2.36–3.22 Å), and (ii) halogen bonding between some fluorine atoms of the 3,5-bis(trifluoromethyl)phenyl fragment and the carbonyl group of Pro112 (3.36–3.48 Å). These latter interactions are present in many protein–ligand complexes.^43^

The nonhydrophobic residues Gln165, Asn109, and His108 are in the central part of the binding pocket, where the O-substituted groups and central moieties of the ligands are placed. Oxygen atoms O-1 and O-2 and the amino group of Gln165 established two hydrogen bonds with lengths of 2.52 and 2.67 Å for **14α** and 2.49 and 2.91 Å for **13α** (Figure 7), which largely contributes to the stabilization of the central moieties of these ligands in the binding site. Such a hydrogen bond was also found for **14β** (2.62 Å) and none for **13β** (Figure 7).

The calculations revealed that the pyranosyl fragment in **14α** and **14β** is a moderate hydrophilic moiety (log *P* = −0.42), which can make close contacts with the polar amino acids Asn109 and His108 (Figure 6A). On the other hand, Figure 6B reveals that the large hydrophobic character of the opposite side of the middle binding region is mainly caused by the presence of Phe268 and Phe264 residues, so a small ligand hydrophobicity would tend to have a weaker binding ability with these residues, but the moderate hydrophilicity of the pyranose fragment in **14α** and **14β** will result in low docking score penalties due to its interaction with these residues. In addition to this, the oxygen atom O-6 participates in the formation of a strong hydrogen bond with Tyr287 in **14α** (2.16 Å) but not in **14β**, suggesting that the former shows better structural requirements for the docking.

Regarding compounds **13α** and **13β**, the different spatial orientations of their hydroxylated pyranosyl fragments lead to different intermolecular interactions. The amino group of the Glu165 residue establishes a hydrogen bond (2.82 Å) with the oxygen atom of the pyranose fragment of ligand **13α**. Moreover, the carbonyl group of Asn109 (2.52 Å) and the basic nitrogen of His108 (2.16 Å) form hydrogen bonds with the hydroxyl group at the 6 position. In the case of **13β**, the hydroxyl groups at 3 and 4 positions and the oxygen atom of Tyr268 act as a H-bonding donor (2.17 Å) and acceptor (2.00 Å), respectively. The log *P* value of this pyranosyl fragment (−1.29) is significantly more negative than that obtained for the galactosyl derivative (see above), which suggests that the observed poor docking score in these ligands in part may be related to the extremely low hydrophobicity of the pyranosyl fragment, which is partially surrounded by a hydrophobic environment (Figure 6B).

Concerning Aprepitant, two hydrogen bonds are formed between the amino group of Glu165 and an oxygen (2.29 Å) and nitrogen (2.67 Å) atom, located on opposite sides of the morpholine moiety. This provokes an optimal anchoring of this fragment within the central region of the binding site. Besides, the log *P* value of this fragment (−0.41) is similar to that of the pyranosyl fragment of galactosyl derivatives, which allows a low scoring penalty due to the interactions with hydrophobic residues of the middle binding region.

Finally, together with Aprepitant, the only ligands that have fragments on the binding site outer region are **14α** and **14β**. The first one has the triazolone group, while the other two have the phenyl group of the benzylidene acetal fragment. These fragments are docked in different binding regions (Figure 6). Thus, the triazolone group (log *P* = −0.64) is located in a more polar region of this binding site region and it interacts through strong H-bonds with Glu193 (2.04 Å) and Trp184 (1.67 Å) and establishes π–π staking interactions and π–alkyl interactions with His197 and Ile182, respectively. By contrast, the benzylidene groups (log *P* = 1.94) of **14α** and **14β** are surrounded by apolar residues like Ala93 and Phe267 and they form π–π staking interactions with Tyr287.

The obtained results show that hydrophobic and π–π stacking interactions play an important role in the binding affinity at the bottom of the binding site. By contrast, hydrogen-bond interactions seem to be the key factor for the docking in the middle binding region, for which the Gln165 residue plays an important role; however, a low hydrophobicity value of the ligand moiety docked in this region seems to provide an unfavorable binding factor. Finally, the presence of molecular fragments in the outer binding region is needed to increase the docking score. The different nature of residues in separate locations of the outer region binding site allows either a hydrophilic fragment, through hydrogen-bonding interactions, or a hydrophobic one, through strong hydrophobic interactions, to increase the binding affinity.

## Conclusions

In summary, we have reported the synthesis of a family of compounds designed as NK1R antagonists, using carbohydrate as the central scaffold. The use of sugars as starting substrates greatly facilitates the obtaining of final products as single isomers, important both for their biological activity and for their possible translation to the market. In addition, the multiple hydroxyl groups allow the regioselective anchoring of different substituents, which facilitates the modulation of the lipophilic/hydrophilic balance and optimizes the bioavailability of the final products. The products synthesized showed a strong affinity and antagonist activity against NK1R and were furthermore shown to be broad-spectrum anticancer agents with high selectivity, comparable to Cisplatin. Among all of the analogues tested, compound **14α** derived from galactose and whose aromatic groups in positions 1 and 2 are in the cis disposition was found to be the most active and the most selective, exhibiting a significant NK1R antagonist effect (*k*_inact_ 0.209 ± 0.103 μM) and a high binding affinity for NK1R (IC_50_ = 50.4 nM, *K*_i_ = 22.4 nM by measuring the displacement of [^125^I] SP from NK1R).

A clear correlation between antagonist and anticancer activities was observed by comparing the potency of the NK1R inhibitory activity of this galactosyl derivative **14α** with two other synthetic derivatives (**13α** and **23β**) and that of Aprepitant.

Interestingly, this galactosyl derivative has shown marked cytotoxic activity against 12 different types of cancer cell lines. Even more interesting is the selectivity observed, with cancer lines being up to 20 times more sensitive than nonmalignant cell lines to the treatment with **14α**. Docking studies on selected carbohydrate derivatives have provided new information on the key role of the 4,6-*O*-benzylidene acetal fragment of **14α** as the most likely responsible for its higher NK1R affinity. Taken together, these results strongly support the possibility of using carbohydrate-based NK1R antagonists as selective anticancer drugs, with the product **14α** being an interesting compound toward this end.

## Experimental Section

### Chemistry: General Procedures

For the reactions that were run under an atmosphere of dry argon, oven-dried glassware and dried solvents were used. Chemicals were obtained from commercial sources and were used without further purification. Thin-layer chromatography (TLC) was carried out on silica gel GF254 (Merck), and compounds were detected by charring with phosphomolybdic acid/EtOH or sulfuric acid/EtOH. For flash chromatography, Merck 230–400 mesh silica gel was used. Chromatographic columns were eluted with a positive pressure of air, and eluents are given as volume-to-volume ratios (v/v). Nuclear magnetic resonance (NMR) spectra were recorded with Bruker Avance 500 MHz spectrometers. Chemical shifts are reported in ppm, and coupling constants are reported in Hz. High-resolution mass spectra (HRMS) were recorded in the Centro de Investigación, Tecnología e Innovación in the University of Seville with a Kratos MS-80RFA 241-MC apparatus. Different ionization methods than chemical ionization (CI) are indicated. Optical rotations were determined with a Perkin-Elmer 341 polarimeter. Melting points were measured with a Stuart SMP3 apparatus in open-ended capillary tubes. A Waters Alliance 2690 HPLC instrument with an ACE Excel C18 (4.6 mm × 100 mm, 2 μm particle size) column was used for analytical high-performance liquid chromatography (HPLC) analyses. The elution conditions were as follows: CH_3_CN/H_2_O, 70% (v/v) CH_3_CN gradient with 0.1% formic acid in 7 min, flow rate 1.0 mL/min, calculation of the relative purity of each compound at 254 nm. For compound **22β**, 80% (v/v) CH_3_CN gradient with 0.1% formic acid in 7 min was used. The purity of all tested compounds was above 95%.

#### Phenyl 2,3,4,6-Tetra-*O*-acetyl-1-thio-β-d-galactopyranoside (**2β**)^24^

To a solution of 3.90 g of d-galactose pentaacetate (10.00 mmol) in dry dichloromethane (40 mL) at 0 °C under an argon atmosphere, 4.93 mL of boron trifluoride etherate (40.00 mmol) was added dropwise. After 15 min of stirring at room temperature, 1.07 mL of thiophenol (10.50 mmol) was added. After stirring overnight, the starting material was consumed. The reaction mixture was quenched with a saturated NaHCO_3_ aqueous solution. The aqueous phase was extracted with dichloromethane (2 × 40 mL), and the combined organic phases were washed with saturated NaCl aqueous solution and dried with anhydrous Na_2_SO_4_. The solvent was evaporated under reduced pressure and the reaction crude was purified by flash chromatography (EtOAc/hexane, 1:4) to obtain 3.96 g of **2β** (9.00 mmol, 90% yield) as a white solid; *R*_f_ = 0.79 (EtOAc/hexane, 1:1); m.p.: 115–116 °C; ^1^H NMR (500 MHz, CDCl_3_): δ 7.52–7.50 (m, 2H), 7.32–7.31 (m, 3H), 5.42 (d, *J* = 2.7 Hz, 1H), 5.24 (t, *J* = 10.0 Hz, 1H), 5.05 (dd, *J* = 3.3, 9.9 Hz, 1H), 4.72 (d, *J* =, 10.0 Hz, 1H), 4.19 (dd, *J* = 6.9, 11.4 Hz, 1H), 4.12 (dd, *J* = 6.2, 11.3 Hz, 1H), 3.94 (t, *J* = 6.9 Hz, 1H), 2.12 (s, 3H), 2.10 (s, 3H), 2.04 (s, 3H), 1.97 (s, 3H) ppm; ^13^C NMR (125 MHz, CDCl_3_): δ 170.5, 170.3, 170.2, 169.6, 132.8, 132.6, 129.1, 128.3, 86.8, 74.6, 72.2, 67.5, 67.4, 61.8, 53.6, 21.0, 20.8(2C), 20.7 ppm. [α]_D_^20^: +3.6 (*c*1, chloroform). HRMS: calcd for C_20_H_25_O_9_S: [M + H] ^+^ 441.1219, found 441.1200 (−4.4 ppm)

#### Phenyl 1-Thio-β-d-galactopyranoside (**3β**)

To a solution of 3.96 g of **2β** (9.00 mmol) in methanol, at 0 °C under an argon atmosphere, 36.00 mL of a 1 M sodium methoxide methanolic solution (36.00 mmol) was added dropwise. After stirring for 35 min, the starting material was consumed. The reaction mixture was neutralized with acid resin and filtered to obtain 2.40 g of **3β** (8.80 mmol, quantitative yield) as a white solid, which was used in the next reaction without further purification; *R*_f_ = 0.72 (EtOAc/hexane, 1:1); m.p.: 114–115 °C; ^1^H NMR (500 MHz, MeOD): δ 7.56–7.54 (m, 2H), 7.30–7.27 (m, 2H), 7.24–7.21 (m, 1H), 4.59 (d, *J* = 9.8 Hz, 1H), 3.90 (d, *J* = 3.0 Hz, 1H), 3.78–3.74 (m, 1H), 3.72–3.69 (m, 1H), 3.61 (t, *J* = 9.5 Hz, 1H), 3.57 (t, *J* = 6.1 Hz, 1H), 3.50 (dd, *J* = 3.3, 9.2 Hz, 1H) ppm; ^13^C NMR (125 MHz, MeOD): δ 136.1, 132.2, 129.8, 128.1, 90.4, 80.7, 76.4, 71.1, 70.5, 62.7 ppm. [α]_D_^20^: −28.3 (*c*1, chloroform). HRMS: calcd for C_12_H_16_0_5_NaS: [M + Na] ^+^ 295.0616, found 295.0605 (3.6 ppm).

#### Phenyl 3,4-*O*-Isopropylidene-1-thio-β-d-galactopyranoside (**4β**)

To a suspension of 2.21 g of **3β** (8.10 mmol) in 60.00 mL of 2,2-dimethoxypropane (2,2-DMP) at room temperature under an argon atmosphere, 7.00 mg of 10-camphorsulfonic acid (CSA) (0.03 mmol) was added. After stirring for 48 h, the reaction mixture was neutralized with triethylamine and filtered to remove the ammonium salts formed. The solvent was evaporated under reduced pressure, and the residue obtained was dissolved in the minimum possible amount of toluene and evaporated to dryness. This process was repeated twice to obtain the mixed acetal with a small amount of the desired diol. Then, the crude obtained was dissolved in the minimum possible amount of methanol and a catalytic amount of CSA at 0 °C was added. After 5 min of stirring at room temperature, the reaction mixture was neutralized with triethylamine, the ammonium salts were filtered, and the solvent was evaporated under reduced pressure. The residue obtained was dissolved in toluene and evaporated to dryness. This process was repeated twice, and the reaction crude was purified by flash chromatography (EtOAc/hexane, 1:2) to obtain 2.16 g of **4β** (6.90 mmol, 85% yield) as a white solid; *R*_f_ = 0.40 (EtOAc/hexane, 1:1); m.p.: 92–93 °C; ^1^H NMR (500 MHz, CDCl_3_): δ 7.48–7.46 (m, 2H), 7.25–7.18 (m, 3H), 4.45 (d, *J* = 10.0 Hz, 1H), 4.08–4.03 (m, 2H), 3.91–3.87 (m, 1H), 3.81–3.78 (m, 1H), 3.76–3.72 (m, 1H), 3.58 (bs, 1H), 3.55–3.51 (m, 1H), 3.17 (bs, 1H), 1.35 (s, 3H), 1.26 (s, 3H) ppm; ^13^C NMR (125 MHz, CDCl_3_): δ 132.5, 131.8, 128.8, 127.5, 110.1, 87.2, 79.3, 76.9, 73.6, 71.2, 62.1, 27.8, 26.1 ppm. [α]_D_^20^: +3.7 (*c*1, chloroform). HRMS: calcd for C_15_H_21_O_5_S: [M + H]^+^ 313.1 110, found 313.1 107 (−0.9 ppm).

#### Phenyl 6-*O*-*tert*-Butyldiphenylsilyl-3,4-*O*-isopropylidene-1-thio-β-d-galactopyranoside (**5β**)

To a solution of **4β** (103.10 mg, 0.30 mmol) in dry DMF (2.00 mL) at room temperature under an argon atmosphere, *tert*-butyldiphenylsilyl chloride (TBDPSCI) (0.11 mL, 0.40 mmol) and imidazole (56.34 mg, 0.80 mmol) were added. After 5 h, the reaction was diluted with ethyl acetate (2.00 mL) and quenched with a saturated NH_4_Cl aqueous solution. The aqueous phase was extracted with *n*-pentane (3 × 20.00 mL) and the combined organic phases were dried with anhydrous Na_2_SO_4_. The solvent was evaporated under reduced pressure and the reaction crude was purified by flash chromatography (EtOAc/hexane, 1:4) to obtain 174.49 mg of the desired compound **5β** (0.30 mmol, 96% yield) as a white solid; *R*_f_ = 0.39 (EtOAc/hexane, 1:3); mp.: 50 °C; ^1^H NMR (500 MHz, CDCl_3_): δ 7.72–7.71 (m, 4H), 7.55–7.53 (m, 2H), 7.45–7.36 (m, 6H), 7.28–7.27 (m, 3H), 4.45 (d, *J* = 10.2 Hz, 1H), 4.28 (dd, *J* = 1.9, 5.4 Hz, 1H), 4.08 (t, *J* = 6.2 Hz, 1H), 4.00–3.90 (m, 3H), 3.55 (ddd, *J* = 2.2, 7.1, 10.1 Hz, 1H), 2.43 (d, *J* = 2.0 Hz, 1H), 1.41 (s, 3H), 1.34 (s, 3H), 1.07 (s, 9H) ppm; ^13^C NMR (125 MHz, CDCl_3_): δ 135.8, 133.6, 133.5, 132.6, 132.5, 129.9 (2), 129.2, 128.1, 127.9, 127.8, 110.3, 88.5, 79.2, 73.5, 71.8, 63.2, 28.3, 27.0, 26.5, 19.4 ppm. [α]_D_^20^: +3.3 (*c*1, chloroform). HRMS: calcd for C_31_H_38_O_5_NaSSi: [M + Na]^+^ 573.2107, found 573.2123 (2.8 ppm).

#### Phenyl 6-*O*-*tert*-Butyldiphenylsilyl-2-*O*-(*p*-fluorobenzyl)-3,4-*O*-isopropylidene-1-thio-β-d-galactopyranoside (**7β**)

To a solution of 4.00 g of **5β** (6.07 mmol) in THF (80.00 mL) at room temperature under an argon atmosphere, a solution of 0.73 g of sodium hydride (18.25 mmol) in THF (10.00 mL) was added. After 1 h, 0.90 g of Bu_4_NI (2.43 mmol) was added and the reaction mixture was stirred for 30 min. Then, a solution of 1.10 mL of *p*-fluorobenzyl chloride **6** (9.10 mmol) in THF (5.00 mL) was added. After 48 h, the reaction mixture was quenched with a saturated NH_4_Cl aqueous solution. The aqueous phase was extracted with ethyl acetate (3 × 40 mL), and the combined organic phases were washed with a saturated NaCl aqueous solution and dried with anhydrous Na_2_SO_4_. The solvent was evaporated under reduced pressure and the reaction crude was purified by flash chromatography (EtOAc/hexane, 1:8) to obtain 3.60 g of **7β** (5.46 mmol, 90% yield) as a yellow oil *R*_f_ = 0.81 (EtOAc/hexane, 1:4); ^1^H NMR (500 MHz, CDCl_3_): δ 7.78–7.75 (m, 4H), 7.58–7.56 (m, 2H), 7.49–7.40 (m, 9H), 7.30–7.24 (m, 2H), 7.10–7.04 (m, 2H), 4.84 (d, *J* = 11.3 Hz, 1H), 4.69 (d, *J* = 11.3 Hz, 1H), 4.65 (d, *J* = 9.7 Hz, 1H), 4.34 (dd, *J* = 2.0, 5.6 Hz, 1H), 4.29 (t, *J* = 6.0 Hz, 1H), 4.02–4.00 (m, 2H), 3.92 (td, *J* = 1.9, 6.5 Hz, 1H), 3.55 (dd, *J* = 6.4, 9.7 Hz, 1H), 1.44 (s, 3H), 1.40 (s, 3H), 1.12 (s, 9H) ppm; ^13^C NMR (125 MHz, CDCl_3_): δ 162.5 (d, *J*_CF_ = 244.1 Hz), 135.8(2), 135.5, 135.0, 134.0, 133.9 (d, *J*_CF_ = 3.1 Hz), 133.5 (2C), 131.9, 130.1 (d, *J*_CF_ = 8.4 HZ), 129.9, 129.8, 129.0, 127.9, 127.8, 127.4, 115.2 (d, *J*_CF_ = 21.4 Hz), 110.1, 86.6, 79.9, 78.4, 76.9, 73.6, 72.9, 63.2, 28.0, 27.0, 26.7, 26.5, 19.4 ppm. [α]_D_^20^: +8.4 (*c*1, chloroform). HRMS: calcd for C_38_H_43_O_5_FNaSSi: [M + Na]^+^ 681.2482, found 681.2479 (−0.5 ppm).

#### 6-*O*-*tert*-Butyldiphenylsilyl-2-*O*-(*p*-fluorobenzyl)-3,4-*O*-isopropylidene-α,β-d-galactopyranoside (**8**(α,β))

To a solution of 3.02 g of **7β** (4.59 mmol) in acetone/water 99:1 (120.00 mL) in the darkness at −15 °C, 1.03 g of *N*-bromosuccinimide (NBS) (5.78 mmol) was added. After 30 min, the reaction mixture was quenched with a saturated NaHCO_3_ aqueous solution. The aqueous phase was extracted with dichloromethane (3 × 40.00 mL), and the combined organic phases were dried with anhydrous Na_2_SO_4_. The solvent was evaporated under reduced pressure and the reaction crude was purified by flash chromatography (EtOAc/hexane, 1:8) to obtain 2.37 g of a mixture of both anomers **8α**:**8β** in a 2:1 ratio (4.18 mmol, 91% yield) as a yellow syrup; *R*_f_ = 0.72 (EtOAc/hexane, 1:4).

#### 6-*O*-*tert*-Butyldiphenylsilyl-2-*O*-(*p*-fluorobenzyl)-3,4-*O*-isopropylidene-α-d-galactopyranoside (**8α**)

^1^H NMR (500 MHz, CDCl_3_): δ 7.71–7.66 (m, 4H), 7.43–7.31 (m, 8H), 7.05–7.00 (m, 2H), 5.15 (dd, *J* = 3.7, 5.0 Hz, 1H), 4.78–4.73 (m, 1H), 4.67–4.64 (m, 1H), 4.41–4.28 (m, 2H), 3.96–3.80 (m, 2H), 3.55 (dd, *J* = 7.2, 5.9 Hz, 1H), 2.92 (d, *J* = 4.1 Hz, 1H), 1.40 (s, 3H), 1.36 (s, 3H), 1.05 (s, 9H) ppm; ^13^C NMR (125 MHz, CDCl_3_): δ 162.7, (d, *J*_CF_ = 245.6 Hz), 135.9, 135.8 (2C), 133.9 (d, *J*_CF_ = 3.5 Hz), 133.8 (2C), 133.7, 133.5, 130.0 (d, *J*_CF_ = 8.2 Hz), 129.9, 129.8, 127.8, 115.5 (d, *J*_CF_ = 21.3 Hz), 110.0, 96.3, 91.0, 80.1, 78.0, 76.4, 74.7, 73.3, 73.0, 72.8, 72.2, 68.6, 63.0, 27.8 (2C), 27.0, 26.1, 19.4 ppm. HRMS: calcd for C_32_H_39_O_6_FNaSi: [M + Na]^+^ 589.2398, found 589.2398 (0.1 ppm).

#### 6-*O*-*tert*-Butyldiphenylsilyl-2-*O*-(*p*-fluorobenzyl)-3,4-*O*-isopropylidene-β-d-galactopyranoside (**8β**)

^1^H NMR (500 MHz, CDCl_3_): δ 7.71–7.66 (m, 4H), 7.43–7.31 (m, 8H), 7.05–7.00 (m, 2H), 4.78–4.73 (m, 1H), 4.67–4.64 (m, 1H), 4.41–4.28 (m, 2H), 4.23 (t, *J* = 6.1 Hz, 1H), 3.96–3.80 (m, 2H), 3.37 (t, *J* = 6.5 Hz, 1H), 2.98 (d, *J* = 6.4 Hz, 1H), 1.41 (s, 3H), 1.36 (s, 3H), 1.05 (s, 9H) ppm; ^13^C NMR (125 MHz, CDCl_3_): δ 162.7, (d, *J*_CF_ = 245.6 Hz), 135.9, 135.8 (2C), 133.8 (2C), 133.7 (d, *J*_CF_ = 3.2 Hz), 133.7, 133.5, 129.9 (d, *J*_CF_ = 8.5 Hz), 129.9, 129.8, 127.8, 115.3 (d, *J*_CF_ = 21.1 Hz), 109.5, 96.3, 91.0, 80.1, 78.0, 76.4, 74.7, 73.3, 72.8, 72.5, 72.2, 68.6, 62.8, 27.8 (2C), 27.0, 26.1, 19.4 ppm. HRMS: calcd for C_32_H_39_O_6_FNaSi: [M + Na]^+^ 589.2398, found 589.2398 (0.1 ppm).

#### (*R*)-{1-[3,5-Bis-(trifluoromethyl)phenyl]ethyl} 6-*O*-*tert*-Butyldiphenylsilyl-2-*O*-*p*-fluorobenzyl-3,4-*O*-isopropylidene-α,β-d-galactopyranoside, (**11α**) and (**11β**)

##### Method A

To a solution of 2.12 g of **8(α,β)** (3.74 mmol) in 50 mL of cyclohexane/dichloromethane 4:1 at room temperature under an argon atmosphere, a catalytic amount of DBU (0.23 mL, 1.50 mmol) and 0.96 mL of a 98% 2,2,2-trichloroacetonitrile solution was added dropwise. After stirring overnight, the reaction mixture was quenched with water, and the organic phase was washed with a saturated NaCl aqueous solution and dried with anhydrous Na_2_SO_4_. The solvent was evaporated under reduced pressure to obtain 2.58 g of trichloroacetimidate *tert*-butyldiphenylsilyl-2-*O*-(*p*-fluorobenzyl)-3,4-*O*-isopropylidene-6-*O*-α,β-d-galactopyranoside, **9(α,β)** (3.63 mmol, 97% yield), as a mixture of both anomers α:β in a 1:1 ratio, as a yellow syrup, which was used directly in the next reaction without further purification; *R*_f_ = 0.69 (EtOAc/hexane, 1:6).

#### 6-*O*-*tert*-Butyldiphenylsilyl-2-*O*-(*p*-fluorobenzyl)-3,4-*O*-isopropylidene-α-d-galactopyranosyl Trichloroacetimidate (**9α**)

^1^H NMR (500 MHz, CDCl_3_): δ 8.58 (s, 1H), 7.71–7.64 (m, 4H), 7.43–7.31 (m, 8H), 7.03–7.00 (m, 2H), 6.38 (d, *J* = 3.4 Hz, 1H), 4.82–4.65 (m, 2H), 4.45–4.34 (m, 2H), 4.07 (td, *J* = 1.9, 6.8 Hz, 1H), 3.98–3.85 (m, 2H), 3.71 (dd, *J* = 3.5, 6.9 Hz, 1H), 1.40 (s, 3H), 1.36 (s, 3H), 1.04 (s, 9H) ppm. HRMS: calc. for C_34_H_39_O_9_FNCl_3_NaSi: [M + Na]^+^ 732.1488, found 732.1471 (−2.3 ppm).

#### 6-*O*-*tert*-Butyldiphenylsilyl-2-*O*-(*p*-fluorobenzyl)-3,4-*O*-isopropylidene-β-d-galactopyranosyl Trichloroacetimidate (9β)

^1^H NMR (500 MHz, CDCl_3_): δ 8.65 (s, 1H), 7.71–7.64 (m, 4H), 7.43–7.31 (m, 8H), 7.03–7.00 (m, 2H), 5.71 (d, *J* = 8.3 Hz, 1H), 4.82–4.65 (m, 2H), 4.45–4.34 (m, 2H), 4.27 (t, *J* = 6.2 Hz, 1H), 3.98–3.85 (m, 2H), 3.64 (dd, *J* = 6.9, 8.2 Hz, 1H), 1.39 (s, 3H), 1.36 (s, 3H), 1.05 (s, 9H) ppm. HRMS: calc. for C_34_H_39_O_9_FNCl_3_NaSi: [M + Na]^+^ 732.1488, found 732.1471 (−2.3 ppm).

To a solution of 0.16 g of the obtained tricloroacetimidate **9(α,β)** (0.22 mmol) and 0.17 g of (*R*)-1-[3,5-bis(trifluoromethyl)phenyl]ethanol (0.66 mmol) with 200.00 mg of a molecular sieve (4 Å) in 7.00 mL of ether at 0 °C under an argon atmosphere, 2.80 mL of trimethylsilyl trifluoromethanesulfonate (0.02 mmol) was added dropwise. After stirring for 1 h at room temperature, the reaction mixture was quenched with a saturated NaHCO_3_ aqueous solution. The resulting suspension was filtered through a pad of Celite. The aqueous phase was extracted with dichloromethane (3 × 40 mL), and the combined organic phases were dried with anhydrous Na_2_SO_4_. The solvent was evaporated under reduced pressure to obtain 0.15 mg (0.19 mmol, 85% yield) of a mixture of both anomers **11α:11β** in a 3:1 ratio, as a yellow syrup. After purification by flash chromatography (EtOAc/hexane, 1:15), 104.00 mg of **11α** (0.13 mmol, 60% yield) and 44 mg of **11β** (0.06 mmol, 25% yield) were obtained.

##### Method B

To a solution of 0.20 g of thioglycoside **7β** (0.30 mmol) and 0.23 g of (1*R*)-1-[3,5-bis(trifluoromethyl)phenyl]ethanol (0.91 mmol) with 200.00 mg of a molecular sieve (4 Å) in 8.00 mL of dichloromethane at 0 °C under an argon atmosphere, 3.90 mL of trimethylsilyl trifluoromethanesulfonate (0.02 mmol) and 0.30 g of NIS (1.50 mmol) were added. After stirring for 1 h at room temperature, the reaction mixture was quenched with a saturated NaHCO_3_ aqueous solution. The resulting suspension was filtered through a pad of Celite. The aqueous phase was extracted with dichloromethane (3 × 40 mL), and the combined organic phases were dried with anhydrous Na_2_SO_4_. The solvent was evaporated under reduced pressure to obtain 193.0 mg (0.24 mmol, 81% yield) of the mixture of both anomers **11α**:**11β** in a 1:1 ratio, as a yellow syrup; *R*_f_ = 0.81 (EtOAc/hexane, 1:6). After purification by flash chromatography (EtOAc/hexane, 1:15), 97.00 mg of **11α** (0.12 mmol, 40% yield) and 97.00 mg of **11β** (0.12 mmol, 40% yield) were obtained.

#### (*R*)-{1-[3,5-Bis-(trifluoromethyl)phenyl]ethyl} 6-*O*-*tert*-Butyldiphenylsilyl-2-*O*-*p*-fluorobenzyl-3,4-*O*-isopropylidene-α-d-galactopyranoside (**11α**)

^1^H NMR (500 MHz, CDCl_3_): δ 7.86 (bs, 2H), 7.82 (bs, 1H), 7.73–7.70 (m, 4H), 7.46–7.37 (m, 6H), 7.21–7.18 (m, 2H), 6.97–6.92 (m, 2H), 4.91 (q, *J* = 6.6 Hz, 1H), 4.61 (d, *J* = 3.1 Hz, 2H), 4.58 (d, *J* = 3.6 Hz, 1H), 4.42 (dd, *J* = 5.5, 7.8 Hz, 1H), 4.29 (dd, *J* = 2.5, 5.5 Hz, 1H), 4.18 (td, *J* = 2.4, 6.4 Hz, 1H), 3.98–3.89 (m, 2H), 3.42 (dd, *J* = 3.7, 7.8 Hz, 1H), 1.49 (d, *J* = 6.6 Hz, 3H), 1.33 (s, 3H), 1.32 (s, 3H), 1.10 (s, 9H) ppm; ^13^C NMR (125 MHz, CDCl_3_): 162.5 (d, *J*_CF_ = 244.8 Hz), 145.9, 135.8(2C), 133.9 (d, *J*_CF_ = 3.2 Hz), 133.7, 133.6, 132.1 (q, *J*_CF_ = 33.5 Hz), 130.0, 129.7 (d, *J*_CF_ = 8.3 Hz), 127.9 (2C), 126.9 (q, *J*_CF_ = 2.4 Hz), 123.5 (q, *J*_CF_ = 272.9 Hz), 121.9 (sept, *J*_CF_ = 4.0 Hz), 115.3 (d, *J*_CF_ = 21.4 Hz), 109.4, 95.0, 76.3, 76.1, 73.5, 72.6, 71.5, 68.9, 63.4, 28.3, 27.0, 26.6, 24.5, 22.8, 19.5 ppm. [α]_D_^20^: +87.8 (*c*1, chloroform). HRMS: calcd for C_42_H_45_O_6_F_7_SiNa: [M + Na]^+^ 829.2771, found 829.2811 (4.1 ppm).

#### (*R*)-{1-[3,5-Bis-(trifluoromethyl)phenyl]ethyl} 6-*O*-*tert*-Butyldiphenylsilyl-2-*O*-*p*-fluorobenzyl-3,4-*O*-isopropylidene-β-d-galactopyranoside (**11β**)

^1^H NMR (500 MHz, CDCl_3_): δ 7.85 (bs, 1H), 7.79–7.74 (m, 3H), 7.66–7.61 (m, 4H), 7.43–7.32 (m, 7H), 7.05–7.00 (m, 2H), 4.97 (q, *J* = 6.5 Hz, 1H), 4.82 (s, 2H), 4.46 (d, *J* = 8.0 Hz, 1H), 4.25 (dd, *J* = 1.7, 5.5 Hz, 1H), 4.17 (dd, *J* = 5.8, 6.7 Hz, 1H), 3.87 (dd, *J* = 6.0, 8.5 Hz, 1H), 3.77–3.71 (m, 2H), 3.42 (t, *J* = 7.5 Hz, 1H), 1.50 (d, *J* = 6.5 Hz, 3H), 1.36 (s, 3H), 1.33 (s, 3H), 1.02 (s, 9H) ppm; ^13^C NMR (125 MHz, CDCl_3_): δ 162.6 (d, *J*_CF_ = 245.2 Hz), 148.4, 146.3, 135.8, 135.7, 134.1 (d, *J*_CF_ = 3.1 Hz), 133.6, 133.4, 131.9 (q, *J*_CF_ = 33.1 Hz), 131.5, 129.9 (d, *J*_CF_ = 8.2 Hz), 129.9, 127.9, 127.8, 125.8 (q, *J*_CF_ = 2.2 Hz), 123.5 (q, *J*_CF_ = 272.3 Hz), 121.4 (sept, *J*_CF_ = 3.8 Hz), 115.3 (d, *J*_CF_ = 21.5 Hz), 110.1, 100.8, 79.7, 79.3, 75.0, 73.6, 73.4, 73.1, 62.6, 28.0, 26.9, 26.4, 25.8, 22.3, 19.3 ppm. [α]_D_^20^: +21.2 (*c*1, chloroform). HRMS: calcd for C_42_H_45_O_6_F_7_SiNa: [M + Na]^+^ 829.2771, found 829.2809 (4.5 ppm).

#### (*R*)-{1-[3,5-Bis-(trifluoromethyl)phenyl]ethyl} 2-*O*-*p*-Fluorobenzyl-3,4-*O*-isopropylidene-α-d-galactopyranoside (**12α**)

To a solution of 0.70 g of **11α** (0.87 mmol) in THF (20 mL) at room temperature under an argon atmosphere, 4.34 mL (4.34 mmol) of 1 M tetrabutylammonium fluoride solution was added dropwise. After stirring for 1 h, the reaction mixture was diluted with ether and quenched with saturated NaCl aqueous solution. The aqueous phase was extracted with ethyl acetate (3 × 40 mL), and the combined organic phases were dried with anhydrous Na_2_SO_4_. The solvent was evaporated under reduced pressure and the reaction crude was purified by flash chromatography (EtOAc/hexane, 1:4) to obtain 0.40 g of **12α** (0.70 mmol, 80% yield) as a yellow syrup; *R*_f_ = 0.13 (EtOAc/hexane, 1:4); ^1^H NMR (500 MHz, CDCl_3_): δ 7.91 (bs, 2H), 7.83 (bs, 1H), 7.20–7.17 (m, 2H), 6.96–6.92 (m, 2H), 4.92 (q, *J* = 6.7 Hz, 1H), 4.64 (d, *J* = 3.6 Hz, 1H), 4.62 (d, *J* = 12.5 Hz, 1H), 4.58 (d, *J* = 12.4 Hz, 1H), 4.47 (dd, *J* = 5.6, 7.9 Hz, 1H), 4.30 (dd, *J* = 2.7, 5.6 Hz, 1H), 4.19–4.16 (m, 1H), 3.97 (dd, *J* = 6.0, 11.8 Hz, 1H), 3.87 (dd, *J* = 3.9, 11.8 Hz, 1H), 3.42 (dd, *J* = 3.6, 8.0 Hz, 1H), 2.25 (dd, *J* = 3.2, 9.3 Hz, 1H), 1.52 (d, *J* = 6.7 Hz, 3H), 1.35 (s, 3H), 1.33 (s, 3H) ppm; ^13^C NMR (125 MHz, CDCl_3_): δ 162.6 (d, *J*_CF_ = 245.6 Hz), 145.7, 133.7 (d, *J*_CF_ = 3.4 Hz), 132.1 (q, *J*_CF_ = 33.3 Hz), 129.7 (d, *J*_CF_ = 8.2 Hz), 126.9 (q, *J*_CF_ = 3.3 Hz), 123.5 (q, *J*_CF_ = 272.8 Hz), 122.0 (sept, *J*_CF_ = 3.6 Hz), 115.3 (d, *J*_CF_ = 21.7 Hz), 109.7, 95.3, 76.3, 75.7, 74.7, 73.1, 71.5, 68.1, 62.9, 28.2, 26.6, 24.5 ppm. [α]_D_^20^: +105.5 (*c*1, chloroform). HRMS (EI): calcd for C_26_H_27_F_7_O_6_: [M]^+^ 568.1792, found 568.1790 (0.5 ppm).

#### (*R*)-{1-[3,5-Bis-(trifluoromethyl)phenyl]ethyl} 2-*O*-*p*-Fluorobenzyl-3,4-*O*-isopropylidene-β-d-galactopyranoside (**12β**)

To a solution of 0.70 g of **11β** (0.87 mmol) in THF (20 mL) at room temperature under an argon atmosphere, 4.34 mL (4.34 mmol) of 1 M tetrabutylammonium fluoride solution was added dropwise. After stirring for 1 h, the reaction mixture was diluted with ether and quenched with saturated NaCl aqueous solution. The aqueous phase was extracted with ethyl acetate (3 × 40 mL), and the combined organic phases were dried with anhydrous Na_2_SO_4_. The solvent was evaporated under reduced pressure and the reaction crude was purified by flash chromatography (EtOAc/hexane, 1:4) to obtain 0.37 g of **12β** (0.65 mmol, 75% yield) as a yellow syrup; *R*_f_ = 0.07 (EtOAc/hexane, 1:4); ^1^H NMR (500 MHz, CDCl_3_): δ 7.75 (bs, 2H), 7.70 (bs, 1H), 7.29–7.26 (m, 2H), 6.95–6.92 (m, 2H), 4.88 (q, *J* = 6.5 Hz, 1H), 4.72 (bs, 2H), 4.39 (d, *J* = 7.9 Hz, 1H), 4.09 (dd, *J* = 5.8, 6.7 Hz, 1H), 4.02–4.00 (m, 1H), 3.71–3.66 (m, 1H), 3.63–3.58, (m, 2H), 3.34 (dd, *J* = 7.0, 7.8 Hz, 1H), 1.59 (bs, 1H), 1.44 (d, *J* = 6.5 Hz, 3H), 1.28 (s, 3H), 1.22 (s, 3H) ppm; ^13^C NMR (125 MHz, CDCl_3_): δ 162.7 (d, *J*_CF_ = 245.7 Hz), 146.6, 134.1 (d, *J*_CF_ = 3.4 Hz), 131.8 (q, *J*_CF_ = 33.3 Hz), 130.0 (d, *J*_CF_ = 8.2 Hz), 126.6 (q, *J*_CF_ = 2.9 Hz), 123.5 (q, *J*_CF_ = 273.0 Hz), 121.6 (sept, *J*_CF_ = 3.6 Hz), 115.3 (d, *J*_CF_ = 21.3 Hz), 110.5, 101.6, 79.7, 79.4, 76.4, 74.0, 73.6, 73.1, 62.4, 27.9, 26.5, 22.9 ppm. [α]_D_^20^: +28.5 (*c*1, chloroform). HRMS (EI): calcd for C_26_H_27_F_7_O_6_: [M]^+^ 568.1792, found 568.1789 (0.5 ppm).

#### (*R*)-{1-[3,5-Bis-(trifluoromethyl)phenyl]ethyl} 2-*O*-*p*-Fluorobenzyl-α-d-galactopyranoside (**13α**)

To a solution of 0.50 g of **12α** (0.88 mmol) in methanol (20.00 mL) at room temperature, a catalytic amount of CSA was added. After stirring overnight, the solvent was evaporated under reduced pressure. The residue obtained was purified by flash chromatography (EtOAc) to obtain 0.46 g of **13α** (0.87 mmol, 99% yield) as a white solid; *R*_f_ = 0.51 (EtOAc); m.p.: 147–149 °C; ^1^H NMR (500 MHz, CDCl_3_): δ 7.89 (bs, 2H), 7.84 (bs, 1H), 7.17–7.14 (m, 2H), 6.98–6.94 (m, 2H), 4.92 (q, *J* = 6.5 Hz, 1H), 4.79 (d, *J* = 3.5 Hz, 1H), 4.53 (d, *J* = 11.9 Hz, 1H), 4.34 (d, *J* = 11.8 Hz, 1H), 4.16–4.10 (m, 2H), 4.02–3.88 (m, 3H), 3.69 (dd, *J* = 3.5, 9.8 Hz, 1H), 2.79 (bs, 1H), 2.34 (d, *J* = 2.6 Hz, 1H), 2.30 (dd, *J* = 3.6, 7.1 Hz, 1H), 3.07 (d, *J* = 6.7 Hz, 3H) ppm; ^13^C NMR (125 MHz, CDCl_3_): δ 162.8 (d, *J*_CF_ = 246.9 Hz), 145.8, 133.4 (d, *J*_CF_ = 3.0 Hz), 132.2 (q, *J*_CF_ = 33.4 Hz), 129.9 (d, *J*_CF_ = 8.2 Hz), 126.9 (q, *J*_CF_ = 3.8 Hz), 123.4 (q, *J*_CF_ = 271.8 Hz), 122.0 (sept, *J*_CF_ = 3.8 Hz), 115.7 (d, *J*_CF_ = 21.3 Hz), 95.1, 75.9, 73.4, 72.2, 70.9, 69.9, 69.1, 63.5, 24.5 ppm. [α]_D_^20^: +121.5 (*c*1, chloroform). HRMS: calcd for C_23_H_23_O_6_F_7_Na: [M + Na]^+^ 551.1281, found 551.1264 (−3.0 ppm).

#### (*R*)-{1-[3,5-Bis-(trifluoromethyl)phenyl]ethyl} 2-*O*-*p*-Fluorobenzyl-β-d-galactopyranoside (**13β**)

To a solution of 0.50 g of **12β** (0.88 mmol) in methanol (200.00 mL) at room temperature, a catalytic amount of CSA was added. After stirring overnight, the solvent was evaporated under reduced pressure. The residue obtained was purified by flash chromatography (EtOAc) to obtain 0.45 g of **13β** (0.85 mmol, 97% yield) as a white solid; *R*_f_ = 0.47 (EtOAc); m.p.: 143–145 °C; ^1^H NMR (500 MHz, CDCl_3_): δ 7.85 (bs, 2H), 7.80 (bs, 1H), 7.38–7.35 (m, 2H), 7.08–7.05 (m, 2H), 4.99 (q, *J* = 6.4 Hz, 1H), 4.97 (d, *J* = 11.5 Hz, 1H), 4.74 (d, *J* = 11.5 Hz, 1H), 4.56 (d, *J* = 7.3 Hz, 1H), 3.98 (dd, *J* = 3.6, 5.9 Hz, 1H), 3.79 (dd, *J* = 3.9, 12.6 Hz, 1H), 3.71 (dd, *J* = 3.4, 8.0 Hz, 2H), 3.63–3.56 (m, 2H), 3.44–3.42 (m, 1H), 1.57 (d, *J* = 6.5 Hz, 3H) ppm; ^13^C NMR (125 MHz, CDCl_3_): δ 162.8 (d, *J*_CF_ = 246.5 Hz), 146.5, 134.2 (d, *J*_CF_ = 3.6 Hz), 131.7 (q, *J*_CF_ = 33.4 Hz), 129.9 (d, *J*_CF_ = 8.2 Hz), 126.6 (q, *J*_CF_ = 3.0 Hz), 123.4 (q, *J*_CF_ = 272.7 Hz), 121.6 (sept, *J*_CF_ = 3.7 Hz), 115.7 (d, *J*_CF_ = 21.2 Hz), 102.7, 79.2, 76.8, 74.3, 73.4, 69.5, 62.7, 22.9 ppm. [α]_D_^20^: +25.3 (*c*1, chloroform). HRMS: calcd for C_23_H_23_F_7_O_6_: [M + Na]^+^ 551.1281, found 551.1262 (−3.0 ppm).

#### (*R*)-{1-[3,5-Bis-(trifluoromethyl)phenyl]ethyl} (*R*)-(4,6-*O*-Benzylidene)-2-*O*-*p*-fluorobenzyl-α-d-galactopyranoside (**14α**)

To a solution of 100.00 mg of **13α** (0.19 mmol) in DMF (15.00 mL) and 0.32 mL of dimethoxymethyl benzene (0.21 mmol) under an argon atmosphere, a catalytic amount of CSA was added. After stirring for 1 h in vacuo at 40 °C, the reaction mixture was quenched with a saturated NaHCO_3_ aqueous solution. The aqueous phase was extracted with dichloromethane (3 × 40 mL), and the combined organic phases were dried with anhydrous Na_2_SO_4_. The solvent was evaporated under reduced pressure and the reaction crude was purified by flash chromatography (EtOAc/hexane, 1:4) to obtain 100.00 mg of **14α** (0.18 mmol, 95% yield) as a white solid; *R*_f_ = 0.48 (EtOAc); m.p.: 158–159 °C. ^1^H NMR (500 MHz, CDCl_3_): δ 7.90 (bs, 2H), 7.84 (bs, 1H), 7.47–7.43 (m, 2H), 7.38–7.35 (m, 3H), 7.16–7.13 (m, 2H), 6.95–6.90 (m, 2H), 5.57 (s, 1H), 4.92 (q, *J* = 6.6 Hz, 1H), 4.81 (d, *J* = 3.5 Hz, 1H), 4.52 (d, *J* = 3.0 Hz, 2H), 4.35–4.34 (m, 1H), 4.32 (dd, *J* = 1.4, 12.6 Hz, 1H), 4.25 (dd, *J* = 3.7, 10.0 Hz, 1H), 4.15–4.12 (m, 1H), 3.84 (bs, 1H), 3.75 (dd, *J* = 3.6, 10.0 Hz, 1H), 1.53 (d, *J* = 6.6 Hz, 3H) ppm; ^13^C NMR (125 MHz, CDCl_3_): δ 162.6 (d, *J*_CF_ = 246.1 Hz), 145.6, 137.6, 133.7 (d, *J*_CF_ = 3.0 Hz), 132.1 (q, *J*_CF_ = 33.6 Hz), 129.7 (d, *J*_CF_ = 8.2 Hz), 129.5, 128.5, 127.0 (d, *J*_CF_ = 2.7 Hz), 126.4, 123.4 (q, *J*_CF_ = 272.1 Hz), 122.0 (sept, *J*_CF_ = 4.0 Hz), 115.4 (d, *J*_CF_ = 21.8 Hz), 101.5, 96.0, 76.3, 76.1, 73.3, 72.7, 69.5, 68.8, 63.3, 24.4 ppm. [α]_D_^20^: +94.6 (*c*1, chloroform). HRMS: calcd for C_30_H_27_O_6_F_7_Na: [M + Na]^+^ 639.1594, found 639.1565 (−4.5 ppm).

#### (*R*)-{1-[3,5-Bis-(trifluoromethyl)phenyl]ethyl} (*R*)-(4,6-*O*-Benzylidene)-2-*O*-*p*-fluorobenzyl-β-d-galactopyranoside (**14β**)

To a solution of 100.00 mg of **13β** (0.19 mmol) in DMF (15.00 mL) and 0.32 mL of dimethoxymethyl benzene (0.21 mmol) under an argon atmosphere, a catalytic amount of CSA was added. After stirring for 1 h in vacuo at 40 °C, the reaction mixture was quenched with a saturated NaHCO_3_ aqueous solution. The aqueous phase was extracted with dichloromethane (3 × 40 mL), and the combined organic phases were dried with anhydrous Na_2_SO_4_. The solvent was evaporated under reduced pressure and the reaction crude was purified by flash chromatography (EtOAc/hexane, 1:4) to obtain 100.00 mg of **14β** (0.18 mmol, 95% yield) as a white solid; *R*_f_ = 0.45 (EtOAc); m.p: 131 °C. ^1^H NMR (500 MHz, CDCl_3_): δ 7.90 (bs, 2H), 7.80 (bs, 1H), 7.49–7.47 (m, 2H), 7.39–7.36 (m, 5H), 7.05–7.01 (m, 2H), 5.52 (s, 1H), 5.03 (q, *J* = 6.6 Hz, 1H), 4.93 (d, *J* = 11.3 Hz, 1H), 4.79 (d, *J* = 11.2 Hz, 1H), 4.57 (d, *J* = 7.6 Hz, 1H), 4.19 (dd, *J* = 1.0, 3.9 Hz, 1H), 4.08 (dd, *J* = 1.4, 12.5 Hz, 1H), 3.98 (dd, *J* = 1.9, 12.5 Hz, 1H), 3.74 (td, *J* = 3.8, 8.9 Hz, 1H), 3.67–3.64 (m, 1H), 3.36–3.35 (m, 1H), 2.45 (d, *J* = 8.4 Hz, 1H), 1.56 (d, *J* = 6.6 Hz, 3H) ppm; ^13^C NMR (125 MHz, CDCl_3_) δ 162.6 (d, *J*_CF_ = 245.6 Hz), 146.6, 137.7, 134.5 (d, *J*_CF_ = 2.8 Hz), 131.6 (q, *J*_CF_ = 33.4 Hz), 129.7 (d, *J*_CF_ = 8.1 Hz), 129.5, 128.4, 126.7, 126.6 (d, *J*_CF_ = 2.8 Hz), 123.6 (q, *J*_CF_ = 272.7 Hz), 121.4 (sept, *J*_CF_ = 4.0 Hz), 115.4 (d, *J*_CF_ = 21.5 Hz), 102.0, 101.6, 79.8, 76.2, 75.6, 74.6, 73.0, 69.0, 66.8, 22.8 ppm. [α]_D_^20^: −2.9 (*c*1, chloroform). HRMS: calcd for C_30_H_27_O_6_NaF_7_: [M + Na]^+^ 639.1594, found 639.1563 (−4.3 ppm).

#### Phenyl 2,3,4-Tri-*O*-acetyl-1-thio-α-l-arabinopyranoside (**16α**)

To a solution of 5.24 g of β-l-arabinose tetraacetate (16.50 mmol) in dry dichloromethane (40 mL) at 0 °C under an argon atmosphere, 8.15 mL of boron trifluoride etherate (65.90 mmol) was added dropwise. After 15 min stirring at room temperature, 1.77 mL of thiophenol (17.30 mmol) was added. After stirring overnight, the starting material was consumed. The reaction mixture was quenched with a saturated NaHCO_3_ aqueous solution. The aqueous phase was extracted with dichloromethane (2 × 40 mL), and the combined organic phases were washed with saturated NaCl aqueous solution and dried with anhydrous Na_2_SO_4_. The solvent was evaporated under reduced pressure and the reaction crude was purified by flash chromatography (EtOAc/hexane, 1:7) to obtain 6.00 g of **16α** (16.30 mmol, quantitative yield) as an orange oil; *R*_f_ = 0.43 (EtOAc/hexane, 1:2); ^1^H NMR (500 MHz, CDCl_3_): δ 7.51–7.50 (m, 2H), 7.33–7.29 (m, 3H), 5.30–5.27 (m, 1H), 5.25 (t, *J* = 8.0 Hz, 1H), 5.11 (dd, *J* = 3.4, 8.5 Hz, 1H), 4.82 (d, *J* = 7.7 Hz, 1H), 4.17 (dd, *J* = 4.3, 12.6 Hz, 1H), 3.68 (dd, *J* = 2.1, 12.7 Hz, 1H), 2.10 (s, 3H), 2.09 (s, 3H), 2.06 (s, 3H) ppm; ^13^C NMR (125 MHz, CDCl_3_): δ 170.4, 170.1, 169.6, 133.5, 132.5, 129.2, 128.2, 87.0, 70.7, 68.8, 67.7, 65.4, 21.1, 21.0, 20.9 ppm. [α]_D_^20^: +2.5 (*c*1, chloroform). HRMS: calcd for C_17_H_20_O_7_NaS: [M + Na] ^+^ 391.0822, found 391.0818 (−1.03 ppm).

#### Phenyl 1-Thio-α-l-arabinopyranoside (**17α**)

To a solution of 6.00 g of **16α** (16.30 mmol) in methanol at 0 °C under an argon atmosphere, 10.00 mL of a 1 M sodium methoxide methanolic solution (10.00 mmol) was added dropwise. After stirring for 35 min, the starting material was consumed. The reaction mixture was neutralized with acid resin and filtered to obtain 3.91 g of **17α** (16.20 mmol, quantitative yield) as a yellow solid, which was used in the next reaction without further purification; *R*_f_ = 0.36 (EtOAc); m.p.: 114–115 °C; ^1^H NMR (500 MHz, CDCl_3_): δ 7.56–7.54 (m, 2H), 7.33–7.28 (m, 3H), 4.48 (d, *J* = 8.9 Hz, 1H), 4.11 (dd, *J* = 2.0, 12.8 Hz, 1H), 3.98 (bs, 1H), 3.71–3.59 (m, 3H), 2.46 (bs, 3H) ppm; ^13^C NMR (125 MHz, CDCl_3_): δ 132.8, 132.5, 129.3, 128.3, 89.4, 74.4, 70.4, 70.0, 68.8 ppm. [α]_D_^20^: −54 (*c*1, chloroform). HRMS: calcd for C_11_H_14_0_4_NaS: [M + Na] ^+^ 265.0505, found 265.0508 (1.1 ppm).

#### Phenyl 3,4-*O*-Isopropylidene-1-thio-α-l-arabinopyranoside (**18α**)

To a suspension of 3.91 g of **17α** (16.20 mmol) in 120.00 mL of 2,2-dimethoxypropane (2,2-DMP) at room temperature under an argon atmosphere, 7.00 mg of 10-camforsulfonic acid (CSA) (0.03 mmol) was added. After stirring for 30 min, the reaction mixture was neutralized with triethylamine and filtered to remove the ammonium salts formed. The solvent was evaporated under reduced pressure, and the residue obtained was dissolved in the minimum possible amount of toluene and evaporated to dryness. This process was repeated twice to obtain the mixed acetal with a small amount of the desired diol. Then, the crude obtained was dissolved in the minimum possible amount of methanol and a catalytic amount of CSA at 0 °C was added. After 5 min of stirring at room temperature, the reaction mixture was neutralized with triethylamine, the ammonium salts were filtered, and the solvent was evaporated under reduced pressure. The residue obtained was dissolved in toluene and evaporated to dryness. This process was repeated twice, and the reaction crude was purified by flash chromatography (EtOAc/hexane, 1:3) to obtain 4.08 g of **18α** (14.41 mmol, 89% yield) as a white solid; *R*_f_ = 0.60 (EtOAc/hexane, 1:1); m.p.: 92–93 °C; ^1^H NMR (500 MHz, CDCl_3_): δ 7.56–7.53 (m, 2H), 7.33–7.28 (m, 3H), 4.53 (d, *J* = 9.2 Hz, 1H), 4.28–4.24 (m, 2H), 4.13–4.09 (m, 1H), 3.82–3.78 (m, 1H), 3.67–3.36 (m, 1H), 2.59 (d, *J* = 2.9 Hz, 1H), 1.46 (s, 3H), 1.36 (s, 3H) ppm; ^13^C NMR (125 MHz, CDCl_3_): δ 132.9, 132.4, 129.2, 128.2, 110.3, 88.4, 78.4, 73.1, 71.7, 65.9, 28.0, 26.3 ppm. [α]_D_^20^: +17.6 (*c*1, chloroform). HRMS: calcd for C_14_H_18_O_4_NaS: [M + Na]^+^ 305.0818, found 305.0818 (−0.05 ppm).

#### Phenyl 2-*O*-(*p*-Fluorobenzyl)-3,4-*O*-isopropylidene-1-thio-α-l-arabinopyranoside (**19α**)

To a solution of 3.85 g of **18α** (13.63 mmol) in THF (100.00 mL) at room temperature under an argon atmosphere, a solution of 1.64 g of sodium hydride (40.89 mmol) in THF (20.00 mL) was added. After 1 h, 2.00 g of IN(Bu)_4_ (5.45 mmol) was added and the reaction mixture was stirred for 30 min. Then, a solution of 2.47 mL of *p*-fluorobenzyl chloride **6** (20.45 mmol) in THF (10.00 mL) was added. After 24 h, the reaction mixture was quenched with a saturated NH_4_Cl aqueous solution. The aqueous phase was extracted with ethyl acetate (3 × 40 mL), and the combined organic phases were washed with a saturated NaCl aqueous solution and dried with anhydrous Na_2_SO_4_. The solvent was evaporated under reduced pressure and the reaction crude was purified by flash chromatography (EtOAc/hexane, 1:10) to obtain 5.05 g of **19α** (13.62 mmol, quantitative yield) as a yellow oil; *R*_f_ = 0.50 (EtOAc/hexane, 1:3); ^1^H NMR (500 MHz, CDCl_3_): δ 7.52–7.50 (m, 2H), 7.39–7.36 (m, 2H), 7.31–7.24 (m, 3H), 7.05–7.00 (m, 2H), 4.79 (d, *J* = 8.2 Hz, 1H), 4.78 (d, *J* = 11.4 Hz, 1H), 4.65 (d, *J* = 11.3 Hz, 1H), 4.31–4.28 (m, 1H), 4.23 (t, *J* = 6.1 Hz, 1H), 4.20 (dd, *J* = 3.8, 13.2 Hz, 1H), 3.77 (dd, *J* = 3.8, 13.0 Hz, 1H), 3.59 (dd, *J* = 6.1, 8.0 Hz, 1H), 1.47 (s, 3H), 1.37 (s, 3H); ^13^C NMR (125 MHz, CDCl_3_): δ 162.6 (d, *J*_CF_ = 245.7 Hz), 134.1, 133.7 (d, *J*_CF_ = 3.1 Hz), 132.0, 130.1 (d, *J*_CF_ = 8.1 Hz), 129.0, 127.6, 115.3, (d, *J*_CF_ = 21.4 Hz), 110.1, 86.5, 78.4, 72.8, 72.7, 64.9, 27.9, 26.3 ppm. [α]_D_^20^: −10 (*c*1, chloroform). HRMS: calcd for C_21_H_23_O_4_FNaS: [M + Na]^+^ 413.1193, found 413.1188 (−1.3 ppm).

#### 2-*O*-(*p*-Fluorobenzyl)-3,4-*O*-isopropylidene-α,β-l-arabinopyranoside (**20**(α,β))

To a solution of 4.60 g of **19α** (12.40 mmol) in acetone/water 99:1 (130.00 mL) in the darkness at −15 °C, 2.80 g of NBS (15.62 mmol) was added. After 15 min, the reaction mixture was quenched with a saturated NaHCO_3_ aqueous solution. The aqueous phase was extracted with dichloromethane (3 × 40.00 mL), and the combined organic phases were dried with anhydrous Na_2_SO_4_. The solvent was evaporated under reduced pressure and the reaction crude was purified by flash chromatography (EtOAc/hexane, 1:5) to obtain 2.94 g of a mixture of both anomers **20α:20β** in a 1:2 ratio (9.93 mmol, 80% yield) as a white solid; *R*_f_ = 0.49 (EtOAc/hexane, 1:1).

#### 2-*O*-(*p*-Fluorobenzyl)-3,4-*O*-isopropylidene-α-l-arabinopyranoside (**20α**)

^1^H NMR (500 MHz, CDCl_3_): δ 7.38–7.32 (m, 2H), 7.05–7.01 (m, 2H), 4.76–4.74 (m, 2H), 4.73 (d, *J* = 6.0 Hz, 1H), 4.26–4.21 (m, 2H), 4.08 (dd, *J* = 2.6 and 13.1 Hz, 1H), 3.82 (dd, *J* = 3.2 and 13.1 Hz, 1H), 3.46 (t, *J* = 5.9 Hz, 1H), 1.47 (s, 3H), 1.36 (s, 3H) ppm; ^13^C NMR (125 MHz, CDCl_3_): δ 162.6 (d, *J* = 245.4 Hz), 133.6 (d, *J* = 2.8 Hz), 129.9 (d, *J* = 8.1 Hz), 115.4 (d, *J* = 21.2 Hz), 109.4, 91.1, 77.4, 74.6, 72.8, 72.1, 60.0, 27.8, 25.9 ppm. HRMS: calcd for C_15_H_19_O_5_FNa: [M + Na]^+^ 321.1109, found 321.1106 (−0.9 ppm).

#### 2-*O*-(*p*-Fluorobenzyl)-3,4-*O*-isopropylidene-β-l-arabinopyranoside (**20β**)

^1^H NMR (500 MHz, CDCl_3_): δ 7.38–7.32 (m, 2H), 7.05–7.01 (m, 2H), 5.17 (d, *J* = 3.4 Hz, 1H), 4.77 (d, *J* = 12.0 Hz, 1H), 4.66 (d, *J* = 11.9 Hz, 1H), 4.37 (t, *J* = 6.1 Hz, 1H), 4.26–4.21 (m, 1H), 4.15 (dd, *J* = 3.0 and 13.1 Hz, 1H), 3.87 (dd, *J* = 1.8 and 13.1 Hz, 1H), 3.58 (dd, *J* = 3.4 and 6.2 Hz, 1H), 1.45 (s, 3H), 1.36 (s, 3H) ppm; ^13^C NMR (125 MHz, CDCl_3_): δ 162.7 (d, *J* = 246.3 Hz,), 133.9 (d, *J* = 3.7 Hz), 130.0 (d, *J* = 8.0 Hz), 115.5 (d, *J* = 21.5 Hz), 110.1, 95.8, 79.5, 76.4, 73.0, 72.5, 62.8, 27.9, 26.1 ppm. HRMS: calcd for C_15_H_19_O_5_FNa: [M + Na]^+^ 321.1109, found 321.1106 (−0.9 ppm).

#### (*R*)-{1-[35-Bis-(trifluoromethyl)phenyl]ethyl} 2-*O*-*p*-Fluorobenzyl-3,4-*O*-isopropylidene-α,β-l-arabinopyranoside, (**22α**) and (**22β**)

##### Method A

To a solution of 1.41 g of **20(α,β)** (4.74 mmol) in 35 mL of dichloromethane at room temperature under an argon atmosphere, a catalytic amount of 98% DBU (0.70 mL, 4.74 mmol) and 2.40 mL of a 98% 2,2,2-trichloroacetonitrile solution (23.68 mmol) was added dropwise. After stirring overnight, the reaction mixture was quenched with water, and the organic phase was washed with a saturated NaCl aqueous solution and dried with anhydrous Na_2_SO_4_. The solvent was evaporated under reduced pressure to give 2.08 g of trichloroacetimidate 2-*O*-(*p*-fluorobenzyl)-3,4-*O*-isopropylidene-6-*O*-α,β-l-arabinopyranoside, **21(α,β)** (4.70 mmol, quantitative yield), as a mixture of both anomers α:β in a 1:2 ratio, as a black syrup, which was used directly in the next reaction without further purification (*R*_f_ = compound unstable in silica gel).

#### 2-*O*-(*p*-Fluorobenzyl)-3,4-*O*-isopropylidene-α-l-arabinopyranosyl Trichloroacetimidate (21α)

^1^H NMR (300 MHz, CDCl_3_): δ 7.37–7.30 (m, 2H); 7.03–6.98 (m, 2H); 5.90 (d, *J* = 6.4 Hz, 1H); 4.77 (s, 2H); 4.45–4.40 (m, 1H); 4.06 (dd, *J* = 1.5 and 13.3 Hz, 1H); 3.99–3.96 (m, 2H); 3.80 (t, *J* = 6.8 Hz, 1H); 1.45 (s, 3H); 1.37 (s, 3H) ppm.

#### 2-*O*-(*p*-Fluorobenzyl)-3,4-*O*-isopropylidene-β-l-arabinopyranoside Trichloroacetimidate (21β)

^1^H NMR (300 MHz, CDCl_3_): δ 7.37–7.30 (m, 2H); 7.03–6.98 (m, 2H); 6.36 (d, *J* = 3.2 Hz, 1H); 4.71 (s, 2H); 4.45–4.40 (m, 1H); 4.34–4.31 (m, 1H); 4.25 (t, *J* = 6.9 Hz, 1H); 4.17 (dd, *J* = 2.9 and 13.2 Hz, 1H); 3.72 (dd, *J* = 3.2 and 7.3 Hz, 1H); 1.44 (s, 3H); 1.37 (s, 3H) ppm.

To a solution of 2.80 g of the obtained tricloroacetimidate **21(α,β)** (4.70 mmol) and 1.21 g of (1*R*)-1-[3,5-bis(trifluoromethyl)phenyl]ethanol (4.70 mmol) with 200.00 mg of a molecular sieve (4 Å) in 95.00 mL of ether at 0 °C under an argon atmosphere, 0.11 mL of trimethylsilyl trifluoromethanesulfonate (0.01 mmol) was added dropwise. After stirring for 24 h at room temperature, the reaction mixture was quenched with a saturated NaHCO_3_ aqueous solution. The resulting suspension was filtered through a pad of Celite. The aqueous phase was extracted with dichloromethane (3 × 40 mL), and the combined organic phases were dried with anhydrous Na_2_SO_4_. The solvent was evaporated under reduced pressure to obtain 1.68 g (3.12 mmol, 66% yield) of a mixture of both anomers **22α**:**22β** in a 1:2 ratio, as a yellow syrup. After purification by flash chromatography (EtOAc/hexane, 1:20; and acetone/hexane, 1:50), 420.00 mg of **22α** (0.75 mmol, 16% yield) and 1.26 mg of **22β** (2.31 mmol, 50% yield) were obtained.

##### Method B

To a solution of 4.85 g of thioglycoside **19α** (12.42 mmol) and 9.62 g of (1*R*)-1-[3,5-bis(trifluoromethyl)phenyl]ethanol (37.26 mmol) with 300.00 mg of a molecular sieve (4 Å) in 150.00 mL of dichloromethane at 0 °C under an argon atmosphere, 14.00 g of NIS (62.10 mmol) was added. After stirring for 6 h at room temperature, the reaction mixture was quenched with a saturated NaHCO_3_ aqueous solution. The resulting suspension was filtered through a pad of Celite. The aqueous phase was extracted with dichloromethane (3 × 40 mL), and the combined organic phases were dried with anhydrous Na_2_SO_4_. The solvent was evaporated under reduced pressure to give 4.41 g (8.20 mmol, 66% yield) of the mixture of both anomers **22α:22β** in a 1:1 ratio, as a yellow syrup. After purification by flash chromatography (EtOAc/hexane, 1:4; and acetone/hexane, 1:50), 2.21 g of **22α** (4.10 mmol, 33% yield), *R*_f_ = 0.64 (EtOAc/hexane, 1:2), and 2.21 g of **22β** (4.10 mmol, 33% yield), *R*_f_ = 0.72 (EtOAc/hexane, 1:2), were obtained.

#### (*R*)-{1-[35-Bis-(trifluoromethyl)phenyl]ethyl} 2-*O*-*p*-Fluorobenzyl-3,4-*O*-isopropylidene-α-l-arabinopyranoside (**22α**)

^1^H NMR (500 MHz, CDCl_3_): δ 7.80 (bs, 2H), 7.78 (bs, 1H), 7.40–7.36 (m, 2H), 7.04 (tt, *J* = 3.0, 8.7 Hz, 2H), 4.94 (q, *J* = 6.5 Hz, 1H), 4.82 (d, *J* = 11.8 Hz, 1H), 4.79 (d, *J* = 11.8 Hz, 1H), 4.59 (d, *J* = 6.8 Hz, 1H), 4.25 (dt, *J* = 4.4, 6.3 Hz, 1H), 4.16 (t, *J* = 6.8 Hz, 1H), 3.87 (dd, *J* = 4.5, 12.8 Hz, 1H), 3.63 (dd, *J* = 4.3, 12.8 Hz, 1H), 3.52 (t, *J* = 7.0 Hz, 1H), 1.52 (d, *J* = 6.5 Hz, 3H), 1.41 (s, 3H), 1.35 (s, 3H) ppm; ^13^C NMR (125 MHz, CDCl_3_): δ 162.6, (d, *J*_CF_ = 245.7 Hz), 146.6, 134.0 (d, *J*_CF_ = 2.9 HZ), 131.7 (q, *J*_CF_ = 33.3 Hz), 129.9 (d, *J*_CF_ = 8.0 Hz), 126.4 (q, *J*_CF_ = 3.0 Hz), 123.5 (q, *J*_CF_ = 272.8 Hz), 121.4 (sept, *J*_CF_ = 3.9 Hz), 115.3 (d, *J*_CF_ = 21.3 Hz), 110.3, 100.8, 79.7, 78.0, 75.0, 72.9, 72.7, 62.5, 27.8, 26.0, 22.6 ppm. [α]_D_^20^: +11 (*c*1, chloroform). HRMS: calcd for C_25_H_25_O_5_F_7_Na: [M + Na]^+^ 561.1482, found 561.1477 (−1.04 ppm).

#### (*R*)-{1-[3,5-Bis-(trifluoromethyl)phenyl]ethyl} 2-*O*-*p*-Fluorobenzyl-3,4-*O*-isopropylidene-β-l-arabinopyranoside (**22β**)

^1^H NMR (500 MHz, CDCl_3_): δ 7.90 (bs, 2H), 7.82 (bs, 1H), 7.22–7.17 (m, 2H), 6.95 (t, *J* = 8.7 Hz, 2H), 4.91 (q, *J* = 6.6 Hz, 1H), 4.64–4.60 (m, 2H), 4.59 (d, *J* = 3.4 Hz, 1H), 4.43 (dd, *J* = 5.7, 7.7 Hz, 1H), 4.31–4.26 (m, 1H), 4.03 (dd, *J* = 2.8, 13.2 Hz, 1H), 4.00 (t, *J* = 13.1 Hz, 1H), 3.44 (dd, *J* = 3.4, 7.8 Hz, 1H), 1.52 (d, *J* = 6.6 Hz, 3H), 1.37 (s, 6H) ppm; ^13^C NMR (125 MHz, CDCl_3_): δ 162.5, (d, *J*_CF_ = 245.5 Hz), 145.8, 133.8 (d, *J*_CF_ = 2.9 Hz), 132.0 (q, *J*_CF_ = 33.2 Hz), 129.7 (d, *J*_CF_ = 8.2 Hz), 126.8 (q, *J*_CF_ = 2.9 Hz), 123.4 (q, *J*_CF_ = 272.6 Hz), 121.8 (sept, *J*_CF_ = 3.8 Hz), 115.2 (d, *J*_CF_ = 21.6 Hz), 109.1, 95.1, 76.0, 75.7, 73.6, 72.9, 71.4, 59.3, 28.2, 26.4, 24.5 ppm. [α]_D_^20^: +57 (*c*1, chloroform). HRMS: calcd for C_25_H_25_O_5_F_7_Na: [M + Na]^+^ 561.1482, found 561.1483 (−0.02 ppm).

#### (*R*)-{1-[3,5-Bis-(trifluoromethyl)phenyl]ethyl} 2-*O*-*p*-Fluorobenzyl-α-l-arabinopyranoside (**23α**)

To a solution of 3.55 g of **22α** (6.60 mmol) in methanol (160.00 mL) at room temperature, a catalytic amount of CSA was added. After stirring overnight, the solvent was evaporated under reduced pressure. The residue obtained was purified by flash chromatography (EtOAc/hexane, 1:1) to obtain 3.36 g of **23α** (6.55 mmol, quantitative yield) as a white solid; *R*_f_ = 0.19 (EtOAc/hexane, 1:1); m.p.: 62–65 °C; ^1^H NMR (500 MHz, CDCl_3_): δ 7.81 (bs, 2H), 7.79 (bs, 1H), 7.36–7.32 (m, 2H), 7.06–7.02 (m, 2H), 4.98 (q, *J* = 6.5 Hz, 1H), 4.88 (d, *J* = 11.4 Hz, 1H), 4.71 (d, *J* = 11.6 Hz, 1H), 4.58 (d, *J* = 6.3 Hz, 1H), 3.89 (dd, *J* = 3.5, 5.8 Hz, 1H), 3.77 (dd, *J* = 3.9, 12.6 Hz, 1H), 3.71 (dd, *J* = 3.5, 8.1 Hz, 1H), 3.59–3.56 (m, 1H), 3.47–3.43 (m, 2H), 2.84–2.80 (m, 1H), 1.53 (d, *J* = 6.5 Hz, 3H) ppm; ^13^C NMR (125 MHz, CDCl_3_): δ 162.7 (d, *J*_CF_ = 246.4 Hz), 146.0, 133.9 (d, *J*_CF_ = 3.3 Hz), 131.8 (q, *J*_CF_ = 33.3 Hz), 129.8, (d, *J*_CF_ = 8.9 Hz), 126.4 (q, *J*_CF_ = 2.8 Hz), 123.4 (q, *J*_CF_ = 272.7 Hz), 121.6 (sept, *J*_CF_ = 3.7 Hz), 115.6 (d, *J*_CF_ = 21.6 Hz), 101.0, 78.6, 75.2, 73.7, 72.1, 67.4, 64.7, 22.1 ppm. [α]_D_^20^: +8.1 (*c*1, chloroform). HRMS: calcd for C_22_H_21_O_5_F_7_Na: [M + Na]^+^ 521.1169, found 521.1167 (−0.6 ppm).

#### (*R*)-{1-[3,5-Bis-(trifluoromethyl)phenyl]ethyl} 2-*O*-*p*-Fluorobenzyl-β-l-arabinopyranoside (**23β**)

To a solution of 1.47 g of **22β** (2.75 mmol) in methanol (60.00 mL) at room temperature, a catalytic amount of CSA was added. After stirring overnight, the solvent was evaporated under reduced pressure. The residue obtained was purified by flash chromatography (EtOAc) to obtain 1.35 g of **23β** (2.72 mmol, quantitative yield) as a white solid; *R*_f_ = 0.19 (EtOAc/hexane, 1:1); m.p.: 91–93 °C; ^1^ H NMR (500 MHz, CDCl_3_): δ 7.89 (bs, 2H), 7.83 (bs, 1H), 7.18–7.15 (m, 2H), 6.99–6.95 (m, 2H), 4.92 (q, *J* = 6.6 Hz, 1H), 4.73 (d, *J* = 3.4 Hz, 1H), 4.53 (d, *J* = 11.9 Hz, 1H), 4.32 (d, *J* = 11.8 Hz, 1H), 4.12 (dd, *J* = 3.5, 9.7 Hz, 1H), 4.06–4.05 (m, 1H), 3.93 (dd, *J* = 1.4, 12.6 Hz, 1H), 3.79 (dd, *J* = 1.8, 12.5 Hz, 1H), 3.67 (dd, *J* = 3.4, 9.7 Hz, 1H), 1.82 (bs, 2H), 1.54 (d, *J* = 6.7 Hz, 3H) ppm; ^13^C NMR (125 MHz, CDCl_3_): δ 162.7 (d, *J*_CF_ = 246.5 Hz), 145.9, 133.4 (d, *J*_CF_ = 3.3 Hz), 132.1 (q, *J*_CF_ = 33.3 Hz), 129.8 (d, *J*_CF_ = 8.2 Hz), 126.8 (q, *J*_CF_ = 3.0 Hz), 123.4 (q, *J*_CF_ = 272.6 Hz), 121.9 (sept, *J*_CF_ = 3.8 Hz), 115.5 (d, *J*_CF_ = 21.8 Hz), 95.1, 76.1, 73.0, 72.0, 69.2, 68.7, 62.6, 24.3 ppm. [α]_D_^20^: +12.02 (*c*1, chloroform). HRMS: calcd for C_22_H_21_O_5_F_7_Na: [M + Na]^+^ 521.1169, found 521.1164 (−1.1 ppm).

### Biological Evaluation

#### Cell Culture and Transfection

Cell lines were obtained from the American Type Culture Collection (Manassas, VA). Cell culture media, fetal bovine serum (FBS), and additives were provided by Invitrogen. CHO cells were grown in Dulbecco’s modified Eagle’s medium (DMEM) supplemented with 10% FBS, 100 U/mL penicillin/streptomycin, and 2 mM l-glutamine, at 37 °C in a humidified atmosphere of 95% air and 5% CO_2_. Nonessential amino acids (Invitrogen) were also added to the media.

Transient transfection of the cell lines was performed using electroporation in a 300 μL volume with a total of 10 μg of DNA (pRK5 Neo-NK1 wild type) plasmid up to 500 ng plus pRK5 as carrier DNA to reach 10 μg containing 107 cells in an electroporation buffer (50 mM K_2_HPO_4_, 20 mM CH_3_COOK, 20 mM KOH, and 26 mM MgSO_4_, pH 7.4). After electroporation (280 V, 1 mF, Gene Zapper 450/2500; IBI, New Haven, CT), cells were suspended in a complete medium and seeded into 96-well culture plates at a density of 105 cells per well. First, 96-well culture plates were coated with polyornithine diluted in phosphate-buffered saline (PBS), incubated at 37 °C for 30 min, and then rinsed with PBS before seeding.

#### Enzyme-Linked Immunosorbent Assay (ELISA)

To measure the expression of the transfected receptors, cells were transfected with pRK5-NK1-6His. After 24 h of electroporation, cells were fixed with 4% paraformaldehyde in PBS for 5 min and rinsed three times with PBS. A blocking step of 30 min with PBS + 1% decomplemented FBS was performed before incubation with an anti-6 His primary antibody (0.5 μg/mL) for 30 min. The cells were then rinsed four times for 5 min in PBS + 1% FBS and incubated for 30 min with an antimouse antibody conjugated with horseradish peroxidase (1/1000; Amersham, Orsay, France). The cells were rinsed three times with PBS + 1% FBS and three times with PBS. Afterward, 60 μL of PBS and 20 μL of Supersignal ELISA Femto (Perbio-Pierce, Brebières, France) were added to the wells. The luminescence was read using a Wallac Victor2 (PerkinElmer Life and Analytical Sciences, Courtaboeuf, France)

#### Second Messenger (IP1) Accumulation

Activation/inhibition of the IP pathway by NK1R agonists or antagonists, respectively, was determined using the IP-One dynamic kit (Cisbio Bioassays, Bagnols-sur-Cèze, France). In brief, after transfection, 105 cells were distributed in 100 μL of complete medium into a 96-well assay plate (Greiner Bio-One, Courtaboeuf, France). Twenty-four hours later, the medium was removed and replaced with 40 μL of incubation medium containing the agonist and/or antagonist at the appropriate concentrations. The IP-One assay is based on the accumulation of IP_1_, a downstream metabolite of the IP pathway that is produced by phospholipase C activated by the Gq/11 protein; IP_1_ is stable in the presence of LiCl. The homogeneous time-resolved fluorescence–fluorescence resonance energy transfer (HTRF–FRET) assay was performed as described previously. This assay involves the transfer of energy from a Lumi4TM-Terbium cryptate donor fluorophore to a d_2_ acceptor fluorophore. The assay is an immunoassay that measures competition between native IP1 produced by the cells and IP1 labeled with the d_2_ acceptor, as revealed by a monoclonal antibody against IP_1_ labeled with Lumi4TM-Terbium cryptate. Fifteen microliters of antibody and 15 μL of competitor diluted in lysis buffer provided in the kits were added to the wells after a 30 min incubation at 37 °C with the agonist. As a negative control, some wells only received the donor fluorophore-labeled antibody. After 1 h of incubation at room temperature, fluorescence emissions were measured at both 620 and 665 nm on a RubyStar fluorometer (BMG Labtechnologies, Offenburg, Germany) equipped with a nitrogen laser as the excitation source (337 nm). A 400 μs reading was recorded after a 50 μs delay to eliminate the short-lived fluorescence background from the acceptor fluorophore-labeled antibody. The fluorescence intensities measured at 620 and 665 nm correspond to the total europium cryptate emission and to the FRET signal, respectively. The specific FRET signal was calculated using the following equation
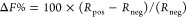
with *R*_pos_ being the fluorescence ratio (665/620 nm) calculated in wells incubated with both donor- and acceptor-labeled antibodies and *R*_neg_ being the same ratio for the negative control incubated only with the donor fluorophore-labeled antibody. The FRET signal (Δ*F*%), which is inversely proportional to the concentration of IP_1_ in the cells, was then transformed into IP_1_ accumulation using a calibration curve prepared on the same plate. It is worth noting that all comparisons of agonist or antagonist effects were done on the same day, on the same culture and plate, and were made against the SP effect. The experiments were repeated at least three times on different cultures. Values corresponding to the low basal activities, determined in unstimulated cells, were first subtracted. Activation/inhibition curves were plotted to the log of agonist or antagonist concentrations and fitted to the Hill equation to extract the EC_50_, the Hill coefficient, and minimal/maximal values.

The inhibitory effect of the specific nonpeptidic NK1 antagonist on IP_1_ accumulations induced by SP was studied according to Arunlakshana and Schild.^44^ Preincubation for 10 min with the antagonist was followed by a 30 min incubation with the antagonist and SP. IP_1_ accumulation was then measured as described above.

#### Cell Lines

MRC-5 (human fetal lung fibroblastic cells) and A549 (human nonsmall cell lung cancer cells) were purchased from the European Collection of Cell Cultures. HaCaT cells (human keratinocytes) were kindly provided by Dr. Motilva (originally Cell Line Service; L#300493-4212). MDA-MB-231 (human breast cancer cells) was purchased from the American Type Culture Collection (ATCC). UACC-62 (human melanoma cells) was obtained from the National Cancer Institute. VH10 (human foreskin fibroblast cells), HepG2 (human hepatocellular carcinoma cells), PC-3 (human prostate cancer cells), and HT29 (human colorectal cancer cells) were generously provided by Dr. Helleday (Karolinska Institute, Sweden). GAMG cells (human glioblastoma cells) were provided by Dr. Ayala (University of Seville, Spain). HNO97 (human tongue cancer cells), A64-CLS (human submaxillary gland adenoma cells), AN3Ca (human endometrial adenocarcinoma cells), Sk-OV-3 (human ovarian cancer cells), KATO III (gastric cancer), Sk-Br-3 (HER2-positive breast cancer), T24 (bladder cancer), and MeWo (Melanoma; BRAF WT) were purchased from Cell Lines Service (CLS). MCF7 (human breast adenocarcinoma cells) and MCF 10 (human mammary epithelial cells) cell lines were a gift from Dr. D. Ruano and Dr. P. Daza (University of Seville, Spain).

To study the possible DNA damage response induced by the tested compound, VC8 (V79 Chinese hamster lung cells mutated in BRCA2, homologous recombination (HR) deficient) and VC8B2 (VC8 cells complemented with human BRCA2 (HR proficient)) were used. These DNA repair-deficient cell lines were kindly provided by Dr. Thomas Helleday.

Cells were maintained in the recommended medium and propagated according to standard protocols. MRC-5, VH10, A549, MCF7, HaCaT, MDA-MB-231, HT29, GAMG, Sk-Br-3, MeWo, HNO97, A64-CLS, SK-OV-3, HepG2, VC8B2, and VC8 were maintained in Dulbecco’s modified Eagle’s medium (DMEM) high-glucose medium. PC-3 and T24 were grown in DMEM-F12. UACC-62 was maintained in RPMI 1640. Except for MCF 10, all media were supplemented with 10% fetal bovine serum, 100 U/mL penicillin, and 100 μg/mL streptomycin. The MCF 10 cell line was maintained in a 1:1 mixture of Dulbecco’s modified Eagle’s medium and Ham’s F12 medium supplemented with a 20 ng/mL epidermal growth factor, 100 ng/mL cholera toxin, 10 μg/mL insulin, and 500 ng/mL hydrocortisone (95%) and horse serum (5%). All cells were cultured in a humidified atmosphere of 95% air and 5% CO_2_ at 37 °C. Cell culture reagents were obtained from Biowest.

#### Binding Assay

Binding assay was carried out by Eurofins Cerep France. The hNK1 binding affinity for compound **14α** was determined by measuring their ability to displace [^125^I]SP (0.05 nM) from U-373MG cells. To define the nonspecific binding, [Sar9,Met(O2)11]-SP (1 μM) was used and the incubation time was extended to 30 min.

The specific ligand binding to the receptors is defined as the difference between the total binding and the nonspecific binding determined in the presence of an excess of unlabeled ligand. All data were averaged from five independent experiments, and the results are expressed as a percent of control specific binding [(measured specific binding/control specific binding) × 100] and as a percent inhibition of control specific binding {100 – [(measured specific binding/control specific binding) × 100]} obtained in the presence of the test compounds.

The IC_50_ value (concentration causing a half-maximal inhibition of control specific binding) and the Hill coefficient (nH) were determined by nonlinear regression analysis of the competition curve generated with mean replicate values using the Hill equation curve fitting (Y = D + [(A – D)/(1 + (C/C_50_)^nH^)], where Y is the specific binding, D is the minimum specific binding, A is the maximum specific binding, C is the compound concentration, C_50_ is IC_50_, and nH is the slope factor). This analysis was performed using a software developed at Cerep (Hill software) and validated by comparison with data generated by commercial software SigmaPlot 4.0 for Windows (©1997 by SPSS Inc.).

The inhibition constant (*K*_i_) was calculated using the Cheng Prusoff equation (*K*_i_ = IC_50_/(1 + (*L*/*K*_D_)), where *L* is the concentration of the radioligand in the assay and *K*_D_ is the affinity of the radioligand for the receptor). A scatchard plot is used to determine the *K*_D_.

#### Cell Viability Assay

Cell viability was estimated with the 3-(4,5-dimethylthiazol-2-yl)-2,5-diphenyltetrazolium bromide (MTT) assay or the resazurin assay. Both assays are redox-based colorimetric techniques based on the capability of viable cells to reduce the yellow product MTT or the blue reagent resazurin into a purple formazan dye or a pink-colored product, respectively. The number of live cells is directly proportional to the amount of the final product created. Exponentially growing cells were seeded in 96-well plates and were allowed to grow during 24 h. The cells were then treated with several concentrations of the tested compounds for 48–96 h (the incubation periods are specified in the figures and table legends) before measuring the cell viability using the MTT assay or the resazurin assay.

For the MTT assay, after the incubation period, the growth medium was removed and 125 μL of MTT solution (1 mg/mL in medium) was added to each well for 3–4 h. Then, 80 μL of 20% sodium dodecyl sulfate (SDS) in 20 mM HCl was added to dissolve the insoluble purple formazan product and plates were incubated for 15 h at 37 °C. The optical density (OD) of each well was measured at 540 nm with a multiwell plate spectrophotometer reader to quantify cell survivals.

For the resazurin assay, after treatment, the medium was removed and 150 μL of resazurin solution (20 μg/mL in medium) was added to each well for 3–6 h. The OD of each well was measured at 540 and 620 nm on a multiwell plate spectrophotometer reader.

In both assays, results were expressed as the percentage of cell viability in relation to untreated cells (controls). All data were averaged from two to five independent experiments and were expressed as the means ± standard error of the mean (SEM). For statistical analysis, the *t*-test (paired, two-tailed) was used. A *p* value >0.05 is not considered statistically significant and is not represented by any symbol, a *p* value <0.05 is considered statistically significant and is indicated with an asterisk (*), a *p* value <0.01 is indicated with a double asterisk (**), and a *p* value <0.001 is indicated with a triple asterisk (***).

Selectivity indices (SIs) are useful to evaluate the anticancer potential in vitro.^45^ SIs were calculated as the mean of the IC_50_ value in the normal cell line divided by the IC_50_ in the cancer cell line obtained in each independent experiment.

Glycolysis inhibition was assessed by measuring concentrations of glucose (initial product of glycolysis) and lactate (final product of glycolysis) in control and treated cells. Briefly, 4 × 10^5^ cells were seeded into 24-well plates. After 10 h, the medium of cells was renewed and drugs were added. Cells were exposed to the tested compounds for 8 h, and glucose and lactate concentrations were determined in cell supernatants using the Accutrend Plus analyzer together with Accutrend glucose strips and BM-Lactate strips (Roche Diagnostics). After calibrating the instrument with glucose and lactate calibration strips, test strips were used to determine glucose and lactate levels via colorimetric-oxidase mediator reactions according to the manufacturer’s instructions.^46^ Results are expressed as a percentage of lactate production and percentage of glucose consumption in relation to untreated cells and are shown as the means ± SEM of two independent experiments.

#### Molecular Modeling

To validate the docking method used with AutoDock Vina, we redocked three cocrystallized ligands existing with the NKR1 protein—CP-99,994 (PDB ID: 6HLL), Aprepitant (PDB ID: 6HLO), and Netupitant (PDB ID: 6HLP)—and then compared the obtained Cartesian coordinates of the docked ligand atoms with those of the native ones, using root-mean-square deviation (RMSD) values. All of the predicted docking poses presented RMSD values lower than 1.5 Å (0.893 Å for CP-99,994, 1.242 Å for Aprepitant, and 1.075 Å for Netupitant) when compared to the experimentally cocrystallized binding pose (see Figure S1). These results indicate that the used molecular docking protocol using AutoDock Vina is satisfactory for inferring the correct binding modes and the interactions of such ligands with NKR1.

Molecular structures of the ligands were optimized in the ground state at the DFT level with the B3LYP^47^ and the 6-31G (d,p) basis set^48^ implemented in the Gaussian 09 Rev.D.O1 package programs.^49^ Molecular docking calculations were performed by AutoDock Vina^50^ and AutodockTools software.^51^ The structure of NKR1 was retrieved from the Protein Data Bank (PDB ID: 6HLO), and all water molecules and cocrystallized ligand were removed from crystallographic structures to prepare the docking receptor. The best docking poses and interactions involved in the binding mode were visualized with Discovery Studio Visualizer (Accelrys Software Inc.).^52^ log *P* (octanol/water partition coefficient) values of the ligands were calculated from the Molinspiration server (http://www.molinspiration.com/) by providing the SMILES code of the fragments of the ligands as input.

## Supporting Information Available

Analytical data concerning characterization of products (NMR), docking validation figure concerning NK1 receptor antagonists, biological evaluation figures, and HPLC traces for lead compounds (PDF)
Molecular formula strings of the prepared compounds (CSV)
Structural data for Aprepitant (PDB)
Structural data for Netupitant (PDB)
Structural data for CP99,994 (PDB)
Structural data for compound **13α** (PDB)
Structural data for compound **13β** (PDB)
Structural data for compound **14α** (PDB)
Structural data for compound **14β** (PDB)

## Supplementary Material

jm1c00793_si_002.csv

jm1c00793_si_003.pdb

jm1c00793_si_004.pdb

jm1c00793_si_005.pdb

jm1c00793_si_006.pdb

jm1c00793_si_007.pdb

jm1c00793_si_008.pdb

jm1c00793_si_009.pdb

## Abbreviations

NK1R: neurokinin 1 receptor
SP: substance P
CarbNK1RAnt: carbohydrate-based NK1R antagonists
GalNK1RAnt: galactose-derived NK1R antagonist
AraNK1RAnt: arabinose-derived NK1R antagonist

## References

[ref1] aSeverini C.; Improta G.; Falconieri-Erspamer G.; Salvadori S.; Erspamer V. The tachykinin peptide family. Pharmacol. Rev. 2002, 54, 285–322. 10.1124/pr.54.2.285. 12037144

[ref2] Santos R.; Ursu O.; Gaulton A.; Bento A. P.; Donadi R. S.; Bologa C. G.; Karlsson A.; Al-Lazikani B.; Hersey A.; Oprea T. I.; Overington J. P. A comprehensive map of molecular drug targets. Nat. Rev. Drug Discovery 2017, 16, 19–34. 10.1038/nrd.2016.230. 27910877 PMC6314433

[ref3] aHökfelt T.; Pernow B.; Wahren J. Substance P: a pioneer amongst neuropeptides. J. Intern. Med. 2001, 249, 27–40. 10.1046/j.0954-6820.2000.00773.x. 11168782

[ref4] Steinhoff M. S.; Mentzer B.; Geppetti P.; Pothoulakis C. H.; Bunnett N. W. Tachykinins and their receptors: contributions to physiological control and the mechanisms of disease. Physiol. Rev. 2014, 94, 265–301. 10.1152/physrev.00031.2013. 24382888 PMC3929113

[ref5] Tattersall F. D.; Rycroft W.; Francis B.; Pearce D.; Merchant K.; MacLeod A. M.; Ladduwahetty T.; Keown L.; C Swain C.; Baker R.; Cascieri M.; Ber E.; Metzger J.; MacIntyre D. E.; Hill R. G.; Hargreaves R. J. Tachykinin NK1 receptor antagonists act centrally to inhibit emesis induced by the chemotherapeutic agent cisplatin in ferrets. Neuropharmacology 1996, 35, 1121–1129. 10.1016/S0028-3908(96)00020-2. 9121615

[ref6] Cao Y. Q.; Mantyh P. W.; Carlson E. J.; Gillespie A. M.; Epstein C. J.; Basbaum A. I. Primary afferent tachykinins are required to experience moderate to intense pain. Nature 1998, 392, 390–394. 10.1038/32897. 9537322

[ref7] Pintér E.; Pozsgai G.; Hajna Z.; Helyes Z.; Szolcsányi J. Neuropeptide receptors as potential drug targets in the treatment of inflammatory conditions. Br. J. Clin. Pharmacol. 2014, 77, 5–20. 10.1111/bcp.12097. 23432438 PMC3895342

[ref8] Kramer M. S. Distinct mechanism for antidepressant activity by blockade of central substance P receptors. Science 1998, 281, 1640–1645. 10.1126/science.281.5383.1640. 9733503

[ref9] Muñoz M.; Coveñas R.; Esteban F.; Redondo M. The substance P/NK-1 receptor system: NK-1 receptor antagonists as anti-cancer drugs. J. Biosci. 2015, 40, 441–463. 10.1007/s12038-015-9530-8. 25963269

[ref10] Muñoz M.; Rosso M.; Robles-Frias M. J.; Salinas-Martin M. V.; Rosso R.; Gonzalez-Ortega A.; Coveñas R. The NK-1 receptor is expressed in human melanoma and is involved in the antitumor action of the NK-1 receptor antagonist aprepitant on melanoma cell lines. Lab. Invest. 2010, 90, 1259–1269. 10.1038/labinvest.2010.92. 20458280

[ref11] Fowler C. J.; Brannstrom G. Substance P enhances forskol in stimulated cyclic AMP production in human UC11MG astrocytoma cells. Methods Find. Exp. Clin. Pharmacol. 1994, 16, 21–28. 7513037

[ref12] Friess H.; Zhu Z.; Liard V.; Shi X.; Shrikhande S. V.; Wang L.; Lieb K.; Korc M.; Palma C.; Zimmermann A.; Reubi J. C.; Buchler M. W. Neurokinin-1 receptor expression and its potential effects on tumor growth in human pancreatic cancer. Lab. Invest. 2003, 83, 731–742. 10.1097/01.LAB.0000067499.57309.F6. 12746482

[ref13] Singh D.; Joshi D. D.; Hameed M.; Qian J.; Gascon P.; Maloof P. B.; Mosenthal A.; Rameshwar P. Increased expression of preprotachykinin-I and neurokinin receptors cells: Implications for bone marrow metastasis. Proc. Natl. Acad. Sci. U.S.A. 2000, 97, 388–393. 10.1073/pnas.97.1.388. 10618428 PMC26673

[ref14] Feng F.; Yang J.; Tong L.; Yuan S.; Tian Y.; Hong L.; Wang W.; Zhang H. Substance P immunoreactive nerve fibres are related to gastric cancer differentiation status and could promote proliferation and migration of gastric cancer cells. Cell Biol. Int. 2011, 35, 623–629. 10.1042/CBI20100229. 21091434

[ref15] aIftikhar H.; Nayyer Ali H.; Farooq S.; Naveed H.; Shahzad-ul-Hussan S. Identification of potential inhibitors of three key enzymes of SARS-CoV2 using computational approach. Comput. Biol. Med. 2020, 122, 103848 10.1016/j.compbiomed.2020.103848. 32658735 PMC7282781

[ref16] aHuang S.-C.; Korlipara V. L. Neurokinin-1 receptor antagonists: a comprehensive patent survey. Expert Opin. Ther. Pat. 2010, 20, 1019–1045. 10.1517/13543776.2010.495121. 20533894

[ref17] Snider R. M.; Constantine J. W.; Lowe J. A.; Longo K. P.; Lebel W. S.; Woody H. A.; Drozda S. E.; Desai M. C.; Vinick F. J.; Spencer R. W.; Hess H.-J. A potent nonpeptide antagonist of the substance P (NK1) receptor. Science 1991, 251, 435–437. 10.1126/science.1703323. 1703323

[ref18] Giardina G. A.; Gagliardi S.; Martinelli M. Antagonists at the neurokinin receptors - recent patent literature. IDrugs 2003, 6, 758–772. 12917772

[ref19] Hale J. J.; Mills S. G.; MacCoss M.; Finke P. E.; Cascieri M. A.; Sadowski S.; Ver E.; Chicchi G. G.; Kurtz M.; Metzger J.; Eirmann G.; Tsou N. N.; Tettersall F. D.; Rupniak N. M.; Williams A. R.; Rycroft W.; Hargraves R.; MacIntyre D. E. Structural optimization affording 2-(*R*)-(1-(*R*)-3,5-bis(trifluoromethyl)phenylethoxy)-3-(*S*)-(4-fluoro)phenyl-4- (3-oxo-1,2,4-triazol-5-yl)methylmorpholine, a potent, orally active, long-acting morpholine acetal human NK-1 receptor antagonist. J. Med. Chem. 1998, 41, 4607–4614. 10.1021/jm980299k. 9804700

[ref20] aDando T. M.; Perry C. M. Aprepitant: a review of its use in the prevention of chemotherapy-induced nausea and vomiting. Drugs 2004, 64, 777–794. 10.2165/00003495-200464070-00013. 15025555

[ref21] aSyed Y. Y. Rolapitant: first global approval. Drugs 2015, 75, 1941–1945. 10.1007/s40265-015-0485-8. 26467681

[ref22] aYin J.; Chapman K.; Clark L. D.; Shao Z.; Borek D.; Xu Q.; Wang J.; Rosenbaum D. M. Crystal structure of the human NK1 tachykinin receptor. Proc. Natl. Acad. Sci. U.S.A. 2018, 115, 13264–13269. 10.1073/pnas.1812717115. 30538204 PMC6310836

[ref23] Recio R.; Vengut-Climent E.; Mouillac B.; Orcel H.; López-Lázaro M.; Calderón-Montaño J. M.; Álvarez E.; Khiar N.; Fernández I. Design, synthesis and biological studies of a library of NK1-receptor ligands based on a 5-arylthiosubstituted 2-amino-4,6-diaryl-3-cyano-4*H*-pyran core: switch from antagonist to agonist effect by chemical modification. Eur. J. Med. Chem. 2017, 138, 644–660. 10.1016/j.ejmech.2017.06.056. 28710964

[ref24] Khiar N.; Fernández I.; Recio R.; López M.; Calderón J. M. Antagonistas de los receptores NK1 derivados de hidratos de carbono, método de obtención y uso médico. WIPO Patent WO2016189179A12016.

[ref25] aLowe J. A. III; Drozda S.; McLean S.; Crawford R.; Bryce D.; Bordner J. *N*-alkyl quinuclidinium substance P antagonists. Bioorg. Med. Chem. Lett. 1994, 4, 1153–1156. 10.1016/S0960-894X(01)80246-8.

[ref26] Takeuchi Y.; Shands E. F.; Beusen D. D.; Marshall G. R. Derivation of a three-dimensional pharmacophore model of substance P antagonists bound to the neurokinin-1 receptor. J. Med. Chem. 1998, 41, 3609–3623. 10.1021/jm9700171. 9733486

[ref27] Yamamoto T.; Nair P.; Jacobsen N. E.; Kulkarni V.; Davis P.; Ma S.; Navratilova E.; Yamamura H. I.; Vanderah T. W.; Porreca F.; Lai J.; Hruby V. J. Biological and conformational evaluation of bifunctional compounds for opioid receptor agonists and neurokinin1 receptor antagonists possessing two penicillamines. J. Med. Chem. 2010, 53, 5491–5501. 10.1021/jm100157m. 20617791 PMC2943425

[ref28] aSteinborn D.; Junicke H. Carbohydrate complexes of platinum-group metals. Chem. Rev. 2000, 100, 4283–4317. 10.1021/cr9903050. 11749349

[ref29] aKhiar N.; Suárez B.; Valdivia V.; Fernández I. Phosphinite thioglygosides as useful ligands for palladium catalyzed asymmetric substitution: synthesis of both enantiomers using natural sugars as catalyst precursors. Synlett 2005, 2005, 2963–2967. 10.1055/s-2005-918963.

[ref30] Ghiviriga I. Selective excitation 1D-NMR experiments for the assignement of the absolute configuration of secondary alcohols. J. Org. Chem. 2012, 77, 3978–3985. 10.1021/jo3003375. 22475048

[ref31] aRosso M.; Muñoz M.; Berger M. The role of neurokinin-1 receptor in the microenvironment of inflammation and cancer ancer. Sci. World J. 2012, 2012, 381434 10.1100/2012/381434. PMC3322385 22545017

[ref32] aChen X.-S.; Li L.-Y.; Guan Y.; Yang J.-M.; Cheng Y. Anticancer strategies based on the metabolic profile of tumor cells: therapeutic targeting of the Warburg effect. Acta Pharmacol. Sin. 2016, 37, 1013–1019. 10.1038/aps.2016.47. 27374491 PMC4973382

[ref33] aMartin-Cordero C.; Leon-Gonzalez A. J.; Calderon-Montano J. M.; Burgos-Moron E.; Lopez-Lazaro M. Pro-oxidant natural products as anticancer agents. Curr. Drug Targets 2012, 13, 1006–1028. 10.2174/138945012802009044. 22594470

[ref34] Linley J. E.; Ooi L.; Pettinger L.; Kirton H.; Boyle J. P.; Peers C.; Gamper N. Reactive oxygen species are second messengers of neurokinin signaling in peripheral sensory neurons. Proc. Natl. Acad. Sci. U.S.A. 2012, 109, E1578–E1586. 10.1073/pnas.1201544109. 22586118 PMC3386117

[ref35] Arnaudeau C.; Tenorio Miranda E.; Jenssen D.; Helleday T. Inhibition of DNA synthesis is a potent mechanism by which cytostatic drugs induce homologous recombination in mammalian cells. Mutat. Res. 2000, 461, 221–228. 10.1016/S0921-8777(00)00052-5. 11056293

[ref36] aLord C. J.; Ashworth A. The DNA damage response and cancer therapy. Nature 2012, 481, 287–294. 10.1038/nature10760. 22258607

[ref37] García-Recio S.; Gascón P. Biological and pharmacological aspects of the NK1-receptor. Biomed. Res. Int. 2015, 2015, 495704 10.1155/2015/495704. 26421291 PMC4573218

[ref38] Rizzi A.; Campia B.; Camarda V.; Molinaria S.; Cantoreggi S.; Regolia D.; Pietra C.; Calo G. In vitro and in vivo pharmacological characterization of the novel NK1 receptor selective antagonist netupitant. Peptides 2012, 37, 86–97. 10.1016/j.peptides.2012.06.010. 22732666

[ref39] aGarnier A.; Vykoukal J.; Hubertus J.; Alt E.; von Schweinitz D.; Kappler R.; Berger M.; Ilmer M. Targeting the neurokinin-1 receptor inhibits growth of human colon cancer cells. Int. J. Oncol. 2015, 47, 151–160. 10.3892/ijo.2015.3016. 25998227

[ref40] aHeuillet E.; Ménager J.; Fardin V.; Flamand O.; Bock M.; Garret C.; Crespo A.; Fallourd A. M.; Doble A. Characterization of a human NK1 tachykinin receptor in the astrocytoma cell line U 373 MG. J. Neurochem. 1993, 60, 868–876. 10.1111/j.1471-4159.1993.tb03231.x. 7679727

[ref41] Guedes I. A.; de Magalhães C. S.; Dardenne L. E. Receptor-ligand molecular docking. Biophys. Rev. 2014, 6, 75–87. 10.1007/s12551-013-0130-2. 28509958 PMC5425711

[ref42] aKujawski J.; Popielarska H.; Myka A.; Drabińska B.; Bernard M. K. The Log P parameter as a molecular descriptor in the computer-aided drug design–an overview. Comput. Methods Sci. Technol. 2012, 18, 81–88. 10.12921/cmst.2012.18.02.81-88.

[ref43] aRendine S.; Pieraccini S.; Forni A.; Sironi M. Halogen bonding in ligand–receptor systems in the framework of classical force fields. Phys. Chem. Chem. Phys. 2011, 13, 19508–19516. 10.1039/c1cp22436k. 21964576

[ref44] Arunlakshana O.; Schild H. O. Some quantitative uses of drug antagonists. Br. J. Pharmacol. Chemother. 1959, 14, 48–58. 10.1111/j.1476-5381.1959.tb00928.x. 13651579 PMC1481829

[ref45] aLópez-Lázaro M. How many times should we screen a chemical library to discover an anticancer drug?. Drug Discovery Today 2015, 20, 167–169. 10.1016/j.drudis.2014.12.006. 25523188

[ref46] Cao X.; Bloomston M.; Zhang T.; Frankel W. L.; Jia G.; Wang B.; Hall N. C.; Koch R. M.; Cheng H.; Knopp M. V.; Sun D. Synergistic antipancreatic tumor effect by simultaneously targeting hypoxic cancer cells with HSP90 inhibitor and glycolysis inhibitor. Clin. Cancer Res. 2008, 14, 1831–1839. 10.1158/1078-0432.CCR-07-1607. 18347186

[ref47] aBecke A. D. Density-functional thermochemistry III. The role of exact exchange. J. Chem. Phys. 1993, 98, 5648–5652. 10.1063/1.464913.

[ref48] aHehre W. J.; Ditchfield R.; Pople J. A. Self—consistent molecular orbital methods XII. Further extensions of gaussian—type basis sets for use in molecular orbital studies of organic molecules. J. Chem. Phys. 1972, 56, 2257–2261. 10.1063/1.1677527.

[ref49] Frisch M. J.; Trucks G. W.; Schlegel H. B.; Scuseria G. E.; Robb M. A.; Cheeseman J. R.; Scalmani G.; Barone V.; Petersson G. A.; Nakatsuji H.; Li X.; Caricato M.; Marenich A.; Bloino J.; Janesko B. G.; Gomperts R.; Mennuci B.; Hratchian H. P.; Ortiz J. V.; Izmaylov A. F.; Sonnenberg J. L.; Williams-Young D.; Ding F.; Lipparini F.; Egidi F.; Goings J.; Peng B.; Petrone A.; Henderson T.; Ranasinghe D.; Zakrzewski V. G.; Gao J.; Rega N.; Zheng G.; Liang W.; Hada M.; Ehara M.; Toyota K.; Fukuda R.; Hasegawa J.; Ishida M.; Nakajima T.; Honda Y.; Kitao O.; Nakai H.; Vreven T.; Throssell K.; Montgomery J. A. Jr.; Peralta J. E.; Ogliaro F.; Bearpark M.; Heyd J. J.; Brothers E.; Kudin K. N.; Staroverov V. N.; Keith T.; Kobayashi R.; Normand J.; Raghavachari K.; Rendell A.; Burant J. C.; Iyengar S. S.; Tomasi J.; Cossi M.; Milam J. M.; Klene M.; Adamo C.; Cammi R.; Ochterski J. W.; Martin R. L.; Morokuma K.; Farkas O.; Foresman J. B.; Fox D. J. Gaussian 09, revision A.02; Gaussian Inc.: Wallingford, CT, 2016.

[ref50] Trott O.; Olson A. J. AutoDock vina: improving the speed and accuracy of docking with a new scoring function, efficient optimization, and multithreading. J. Comput. Chem. 2010, 31, 455–461. 10.1002/jcc.21334. 19499576 PMC3041641

[ref51] Morris G. M.; Huey R.; Lindstrom W.; Sanner M. F.; Belew R. K.; Goodsell D. S.; Olson A. J. AutoDock4 and AutoDockTools4: automated docking with sSelective receptor flexibility. J. Comput. Chem. 2009, 30, 2785–2791. 10.1002/jcc.21256. 19399780 PMC2760638

[ref52] Discovery studio visualizer, version 4.0; software for viewing, sharing, and analyzing protein and modeling data; BIOVIA: San Diego, 2012.

