# Supplementary material for: Carbohydrate-Based NK1R Antagonists with Broad-Spectrum Anticancer Activity
Source: J Med Chem. 2021 Jul 8;64(14):10350–70. doi: 10.1021/acs.jmedchem.1c00793 (PMC8529873; doi:10.1021/acs.jmedchem.1c00793)

Supporting Information

Carbohydrate-based NK1R antagonists with broad-spectrum anticancer activity

Rocío Recio,[#,†] Patricia Lerena, [#,†] Esther Pozo,[†] José Manuel Calderón-Montaño,[‡] Estefanía Burgos-Morón,[‡] Miguel López-Lázaro,[‡] Victoria Valdivia,[†] Manuel Pernia Leal,[†] Bernard Mouillac,[ʃ] Juan Ángel Organero,[¥] Noureddine Khiar,*[£] Inmaculada Fernández.*[†]

[†] Departamento de Química Orgánica y Farmacéutica. Facultad de Farmacia. Universidad de Sevilla. C/ Profesor García González, 2, 41012, Sevilla, Spain.
[‡] Departamento de Farmacología, Facultad de Farmacia. Universidad de Sevilla. C/ Profesor García González, 2, 41012, Sevilla, Spain.
[ʃ]Institut de Génomique Fonctionnelle (IGF), CNRS, INSERM, Univ. Montpellier, F-34094 Montpellier, France.
[¥] Departamento de Química Física. Facultad de Ciencias Ambientales y Bioquímicas and INAMOL, Universidad de Castilla-La Mancha. Avda. Carlos III, s.n., 45071, Toledo, Spain.
[£] Instituto de Investigaciones Químicas, C.S.I.C-Universidad de Sevilla. C/Américo Vespucio, 49, Isla de la Cartuja, 41092, Sevilla, Spain.

inmaff@us.es
khiar@iiq.csic.es

Table of contents

^{1}H-NMR, ^{13}C-NMR of selected compounds S2

NOE studies of 14α and 14β S48

Molecular modelling S50

Biological evaluation S51

Competition Experiments of Cytotoxic effect of 14α in presence of SP S53

HPLC traces for lead compounds S54

Phenyl 2,3,4,6-tetra-*O*-acetyl-1-thio-β-D-galactopyranoside, (**2β**)

^{1}H-NMR (500 MHz, $CDCl_3$)

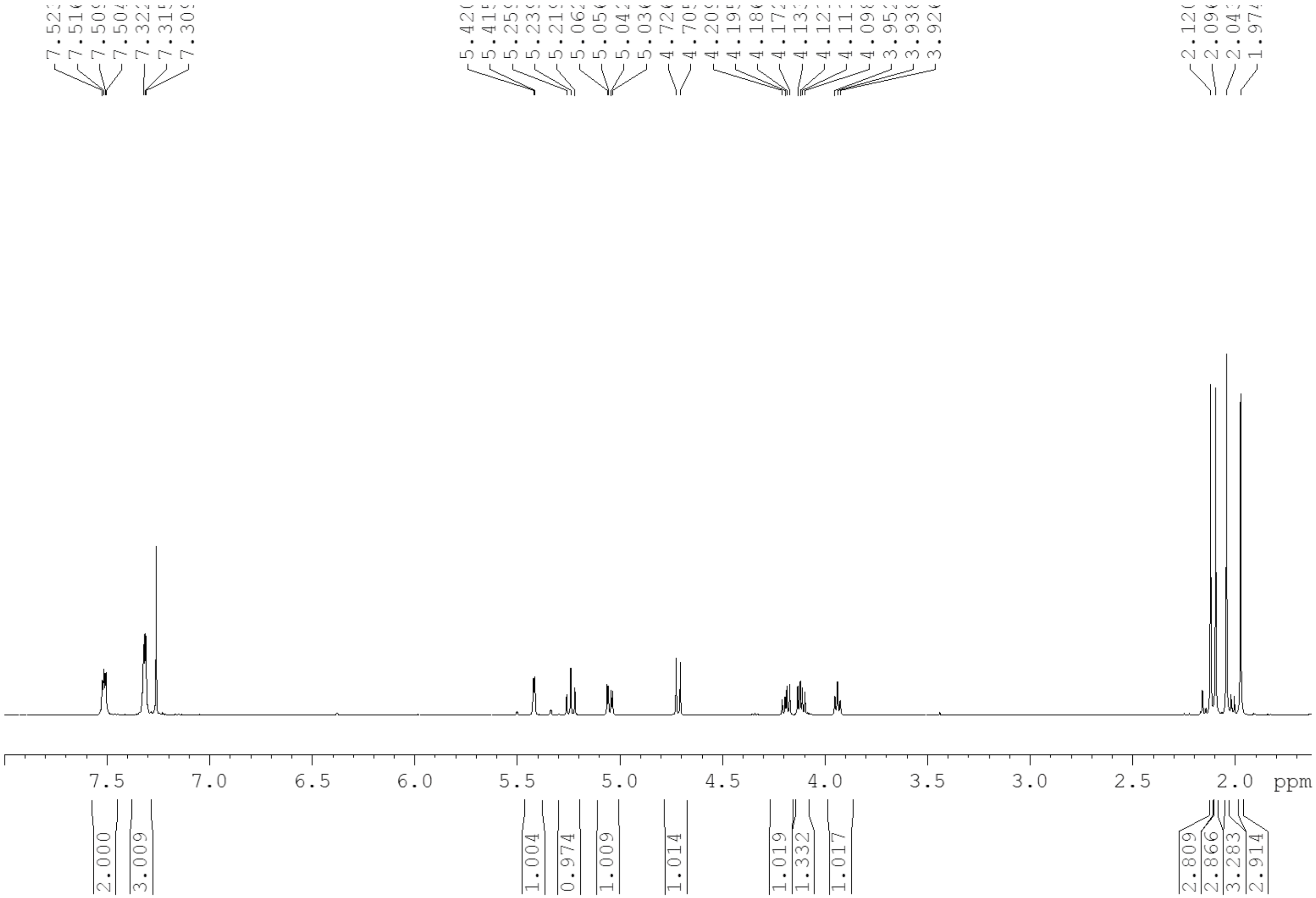

Phenyl 2,3,4,6-tetra-*O*-acetyl-1-thio-β-D-galactopyranoside, (**2β**)

^{13}C-NMR (125 MHz, $CDCl_3$)

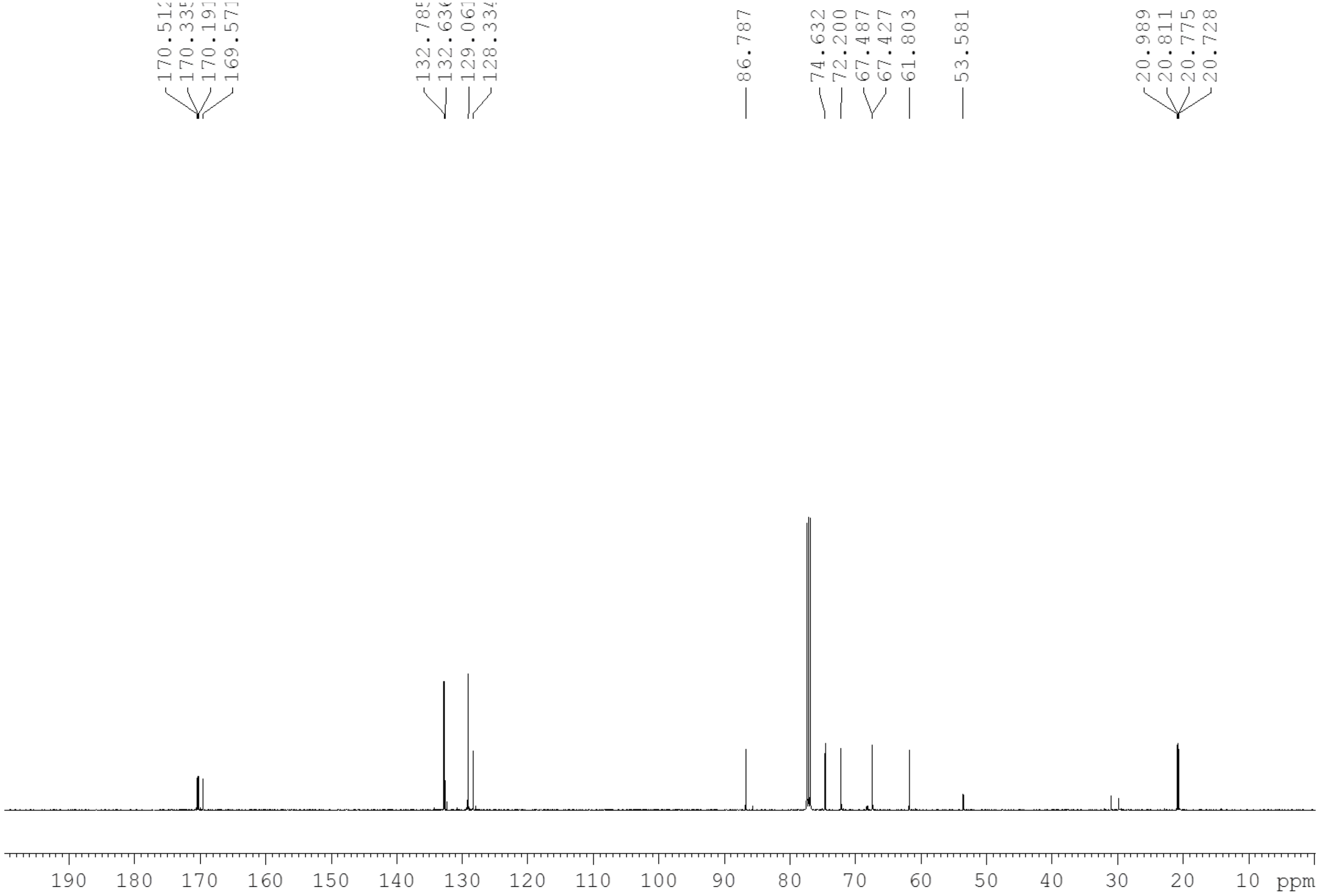

Phenyl 1-thio-β-D-galactopyranoside, (**3β**)

^{1}H-NMR (500 MHz, MeOD)

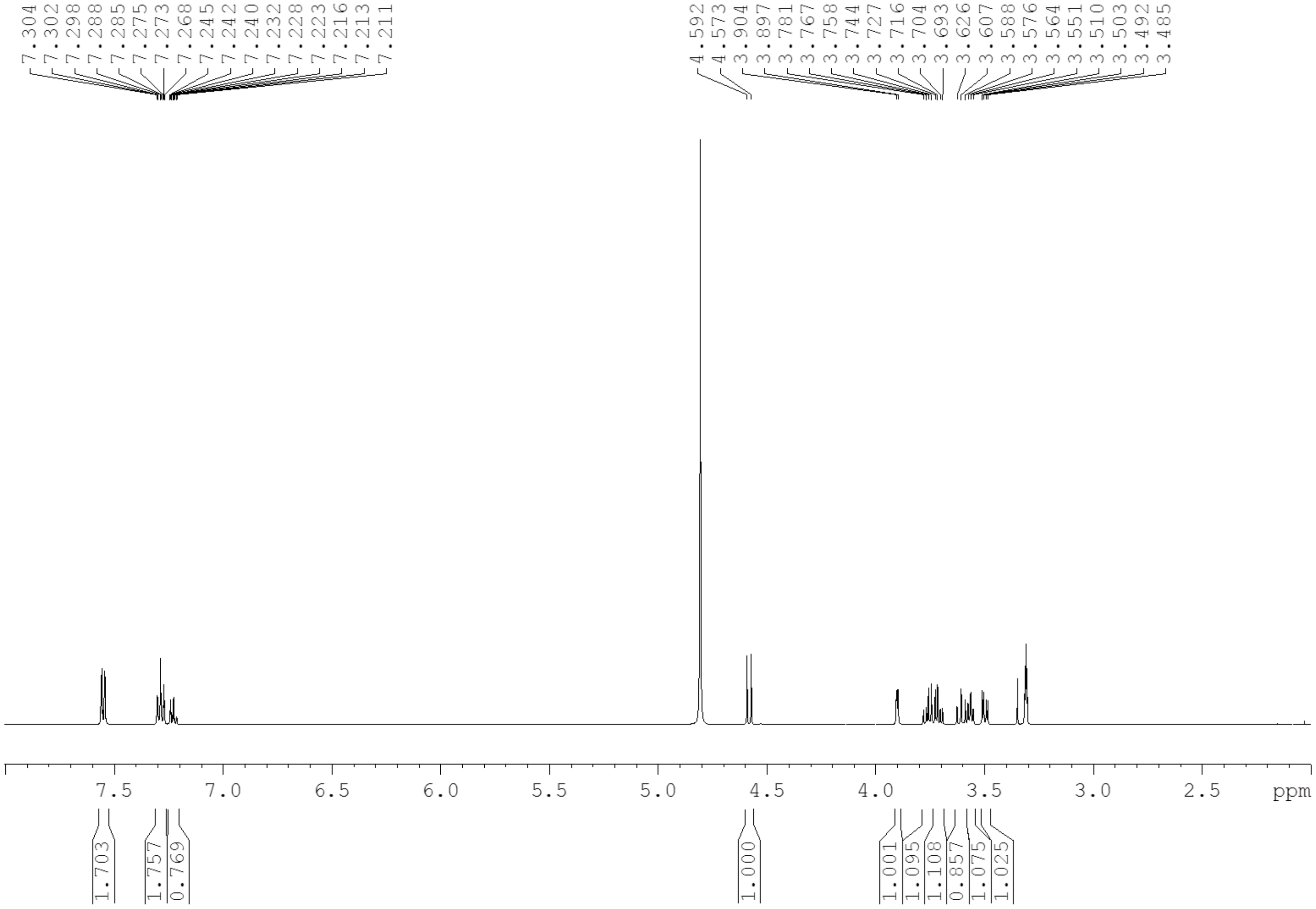

Phenyl 1-thio-β-D-galactopyranoside, (**3β**)
^{13}C-NMR (125 MHz, MeOD)

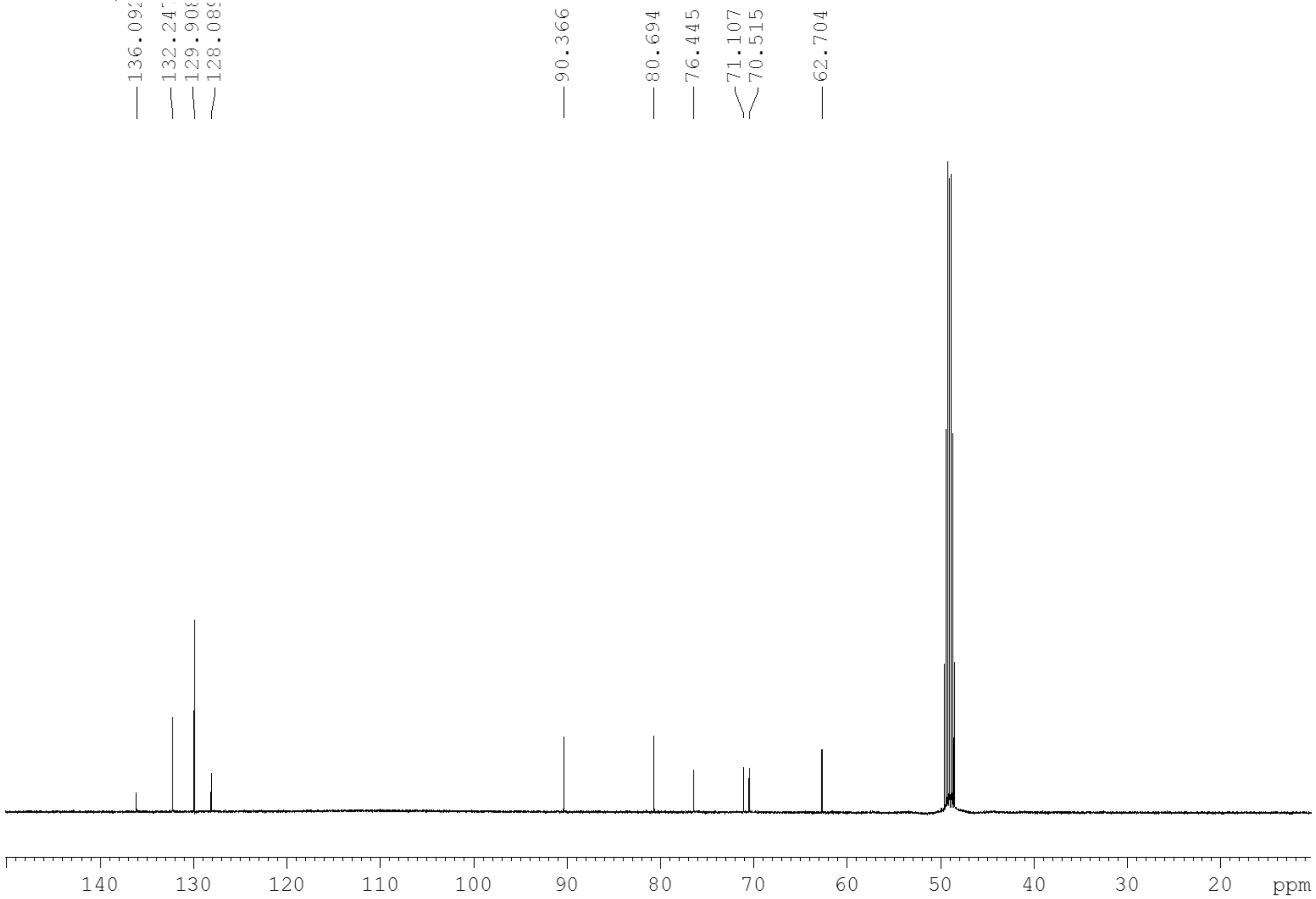

Phenyl 3,4-*O*-isopropylidene-1-thio-β-D-galactopyranoside, **(4β)**

^{1}H-NMR (500 MHz, $CDCl_3$)

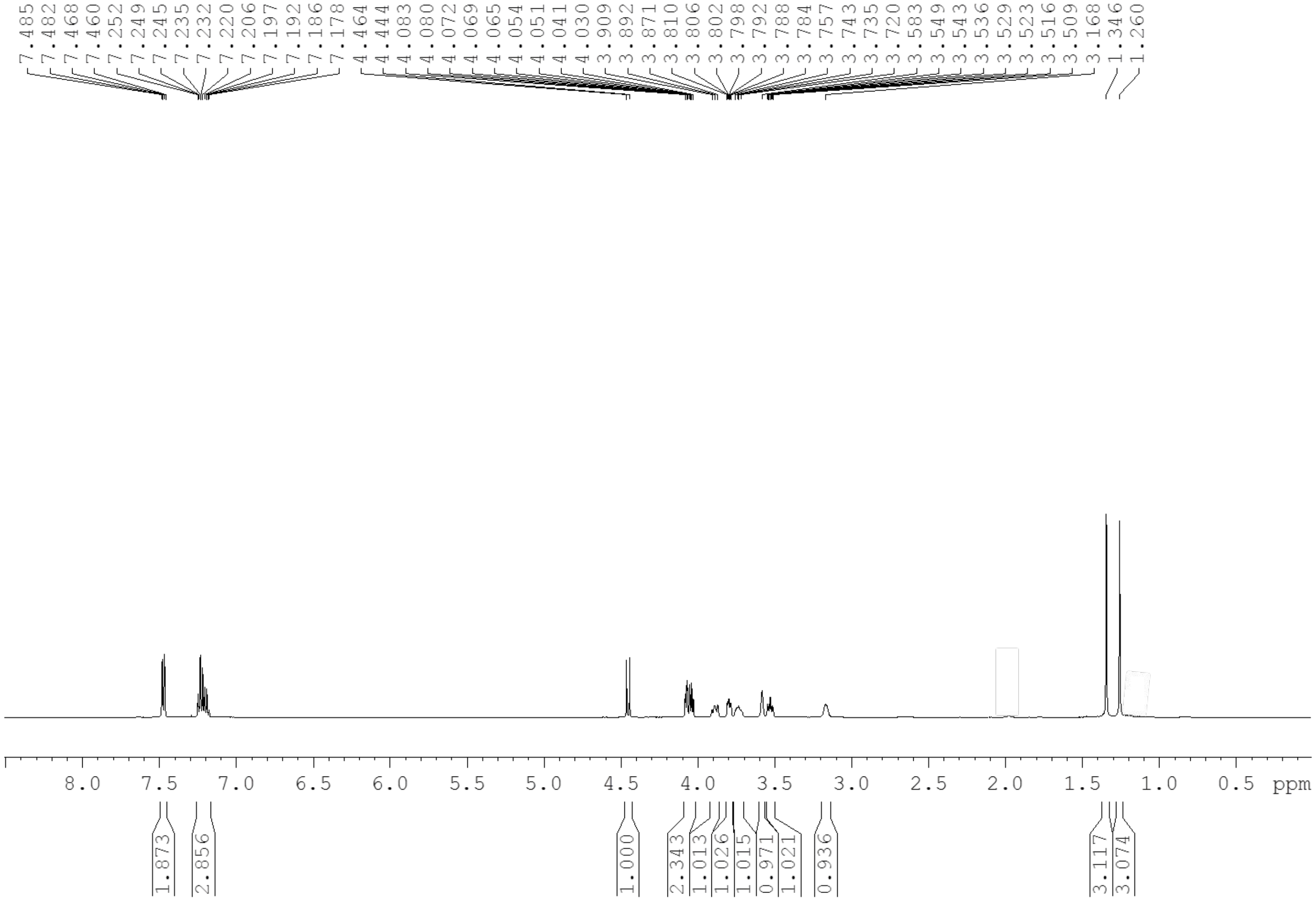

Phenyl 3,4-*O*-isopropylidene-1-thio-β-D-galactopyranoside, (**4β**)
^{13}C-NMR (125 MHz, $CDCl_3$)

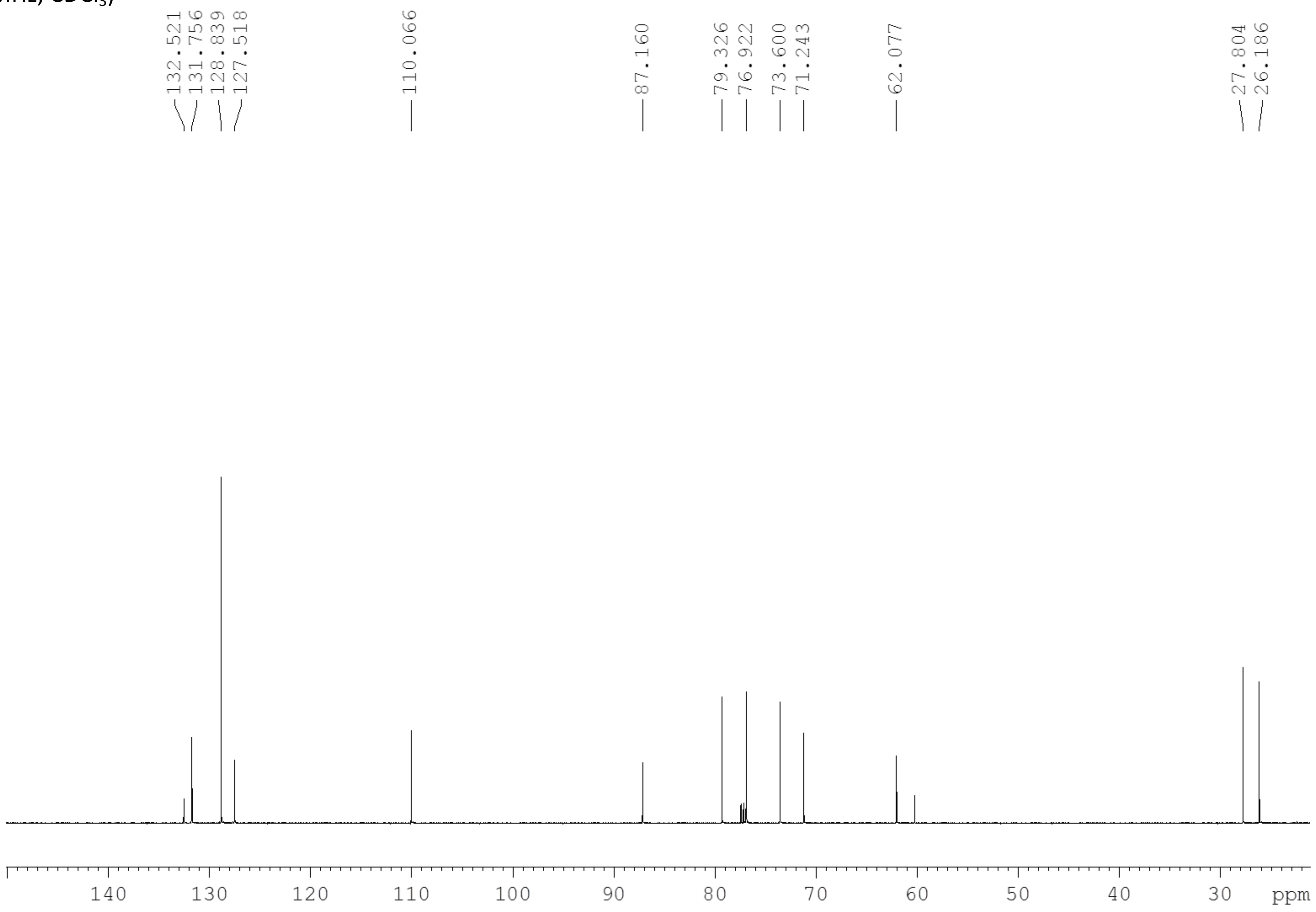

Phenyl 6-*O*-*tert*-butyldiphenylsilyl-3,4-*O*-isopropylidene-1-thio-β-D-galactopyranoside, (**5β**)

^{1}H-NMR (500 MHz, $CDCl_3$)

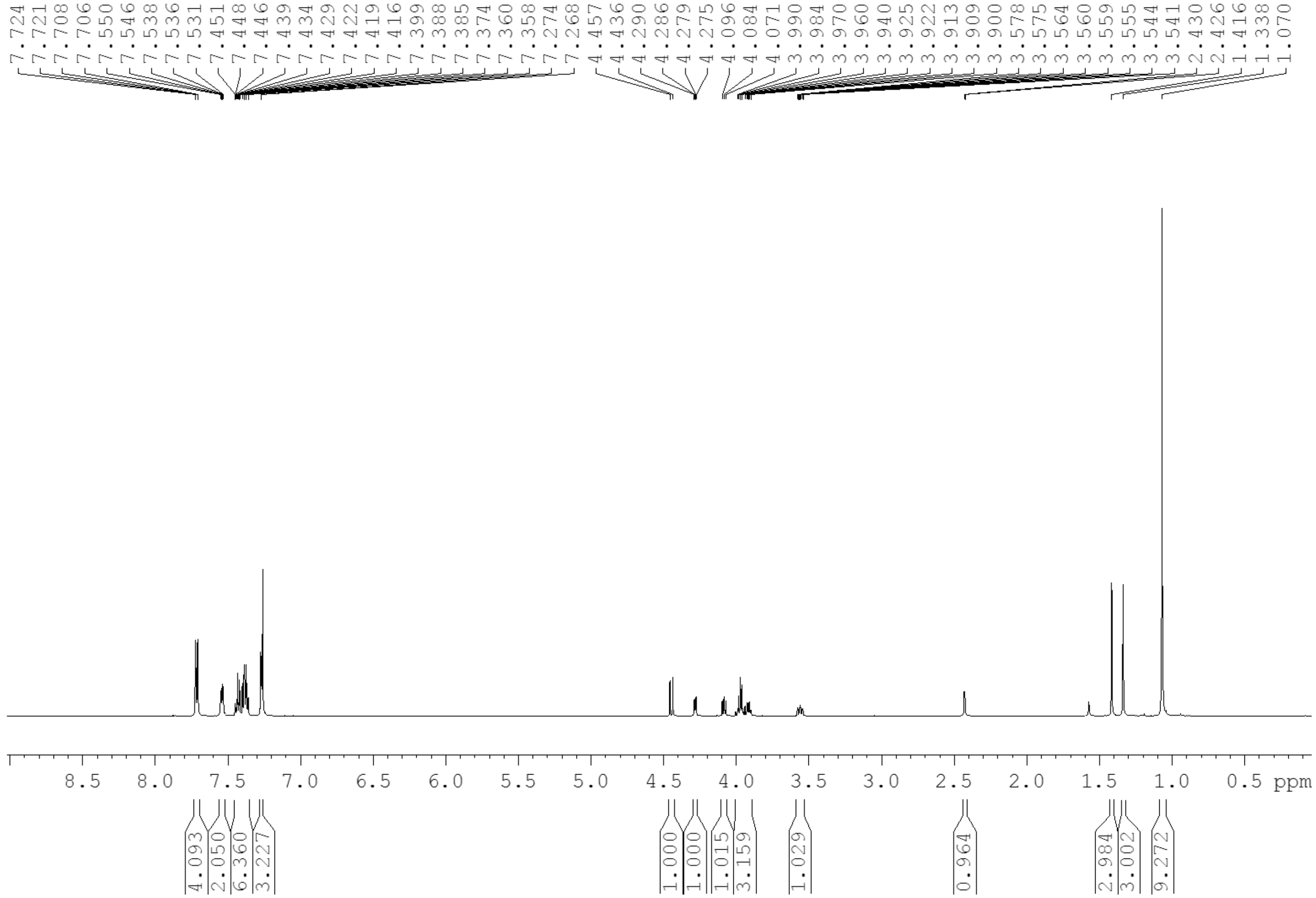

Phenyl 6-*O*-*tert*-butyldiphenylsilyl-3,4-*O*-isopropylidene-1-thio-β-D-galactopyranoside, (**5β**)

^{13}C-NMR (125 MHz, $CDCl_3$)

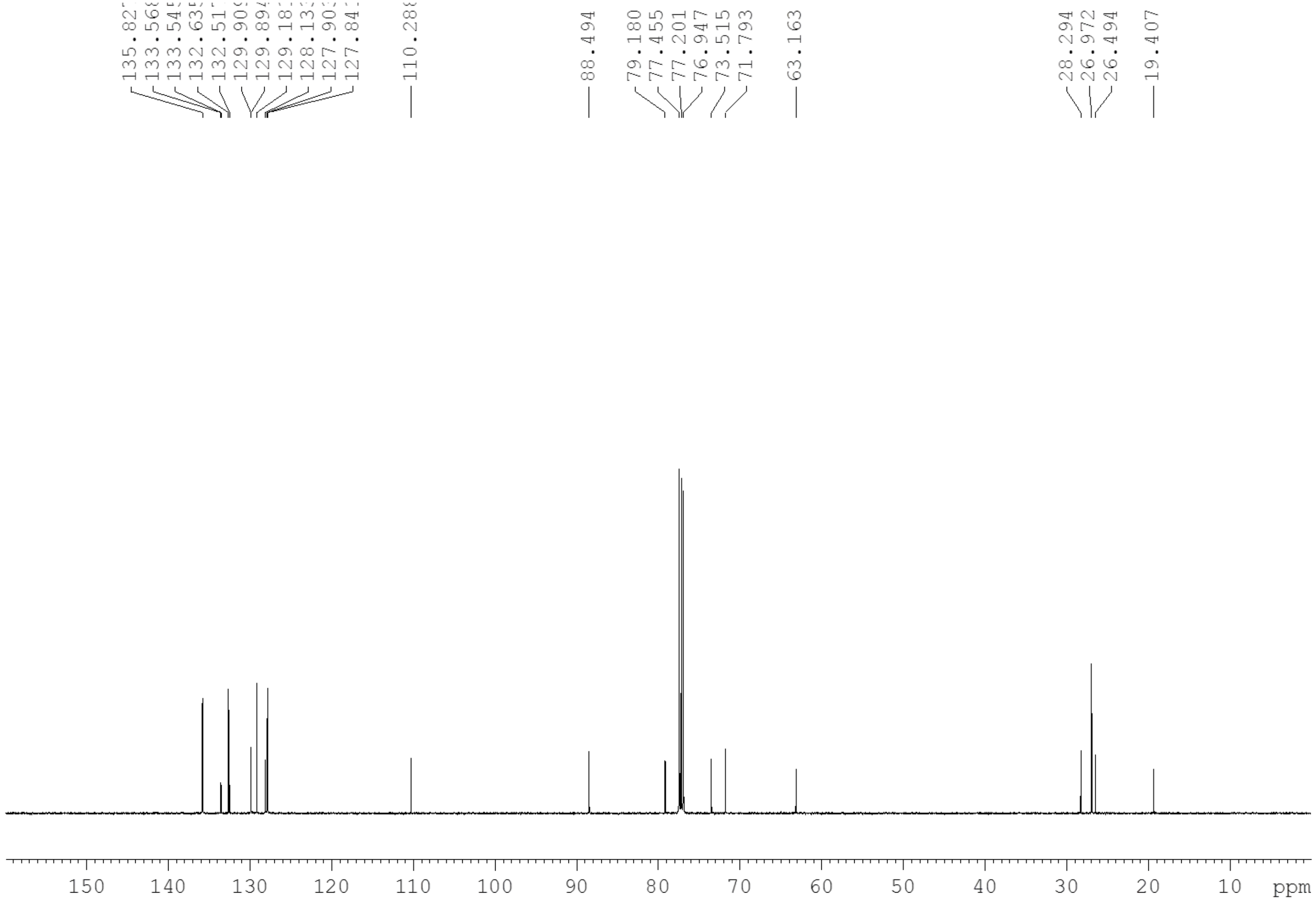

Phenyl 6-*O*-*tert*-butyldiphenylsilyl-2-*O*-(*p*-fluorobenzyl)-3,4-*O*-isopropylidene-1-thio-β-D-galactopyranoside, (**7β**)

^{1}H-NMR (500 MHz, $CDCl_3$)

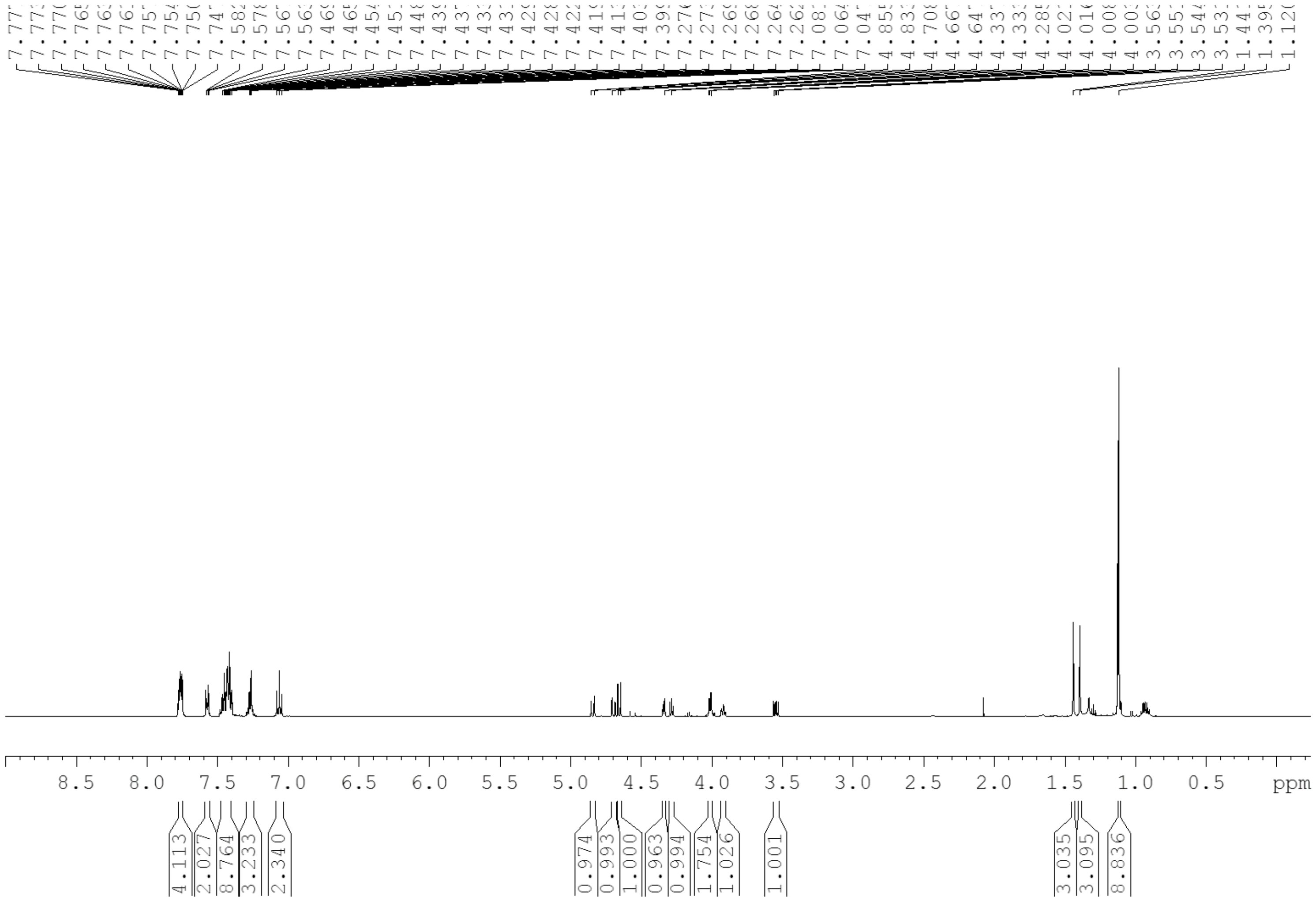

Phenyl 6-*O*-*tert*-butyldiphenylsilyl-2-*O*-(*p*-fluorobenzyl)-3,4-*O*-isopropylidene-1-thio-β-D-galactopyranoside, (**7β**)

^{13}C-NMR (125 MHz, $CDCl_3$)

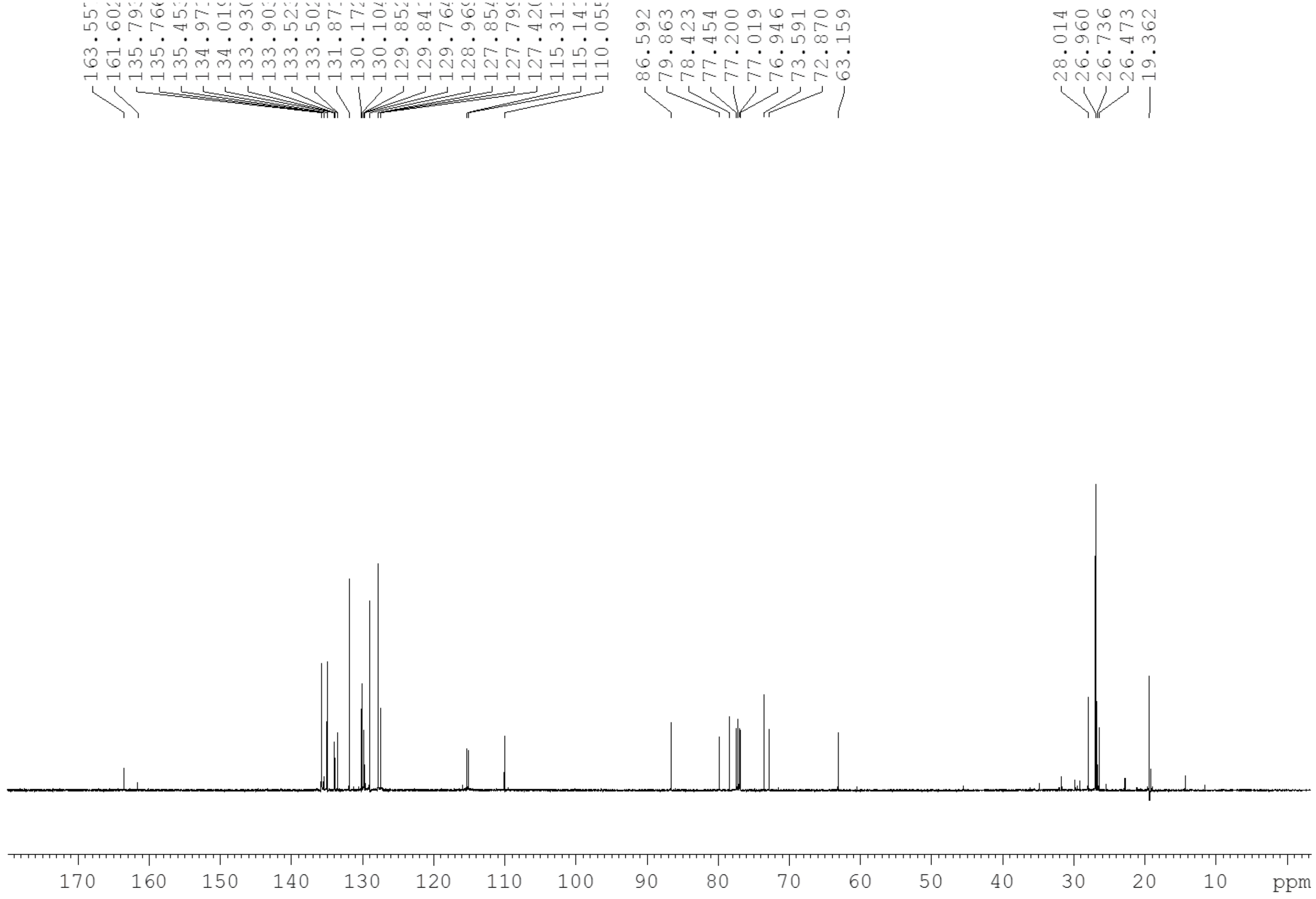

6-*O*-*tert*-Butyldiphenylsilyl-2-*O*-(*p*-fluorobenzyl)-3,4-*O*-isopropylidene-α,β-D-galactopyranoside, (**8**(α,β))

^{1}H-NMR (500 MHz, $CDCl_3$)

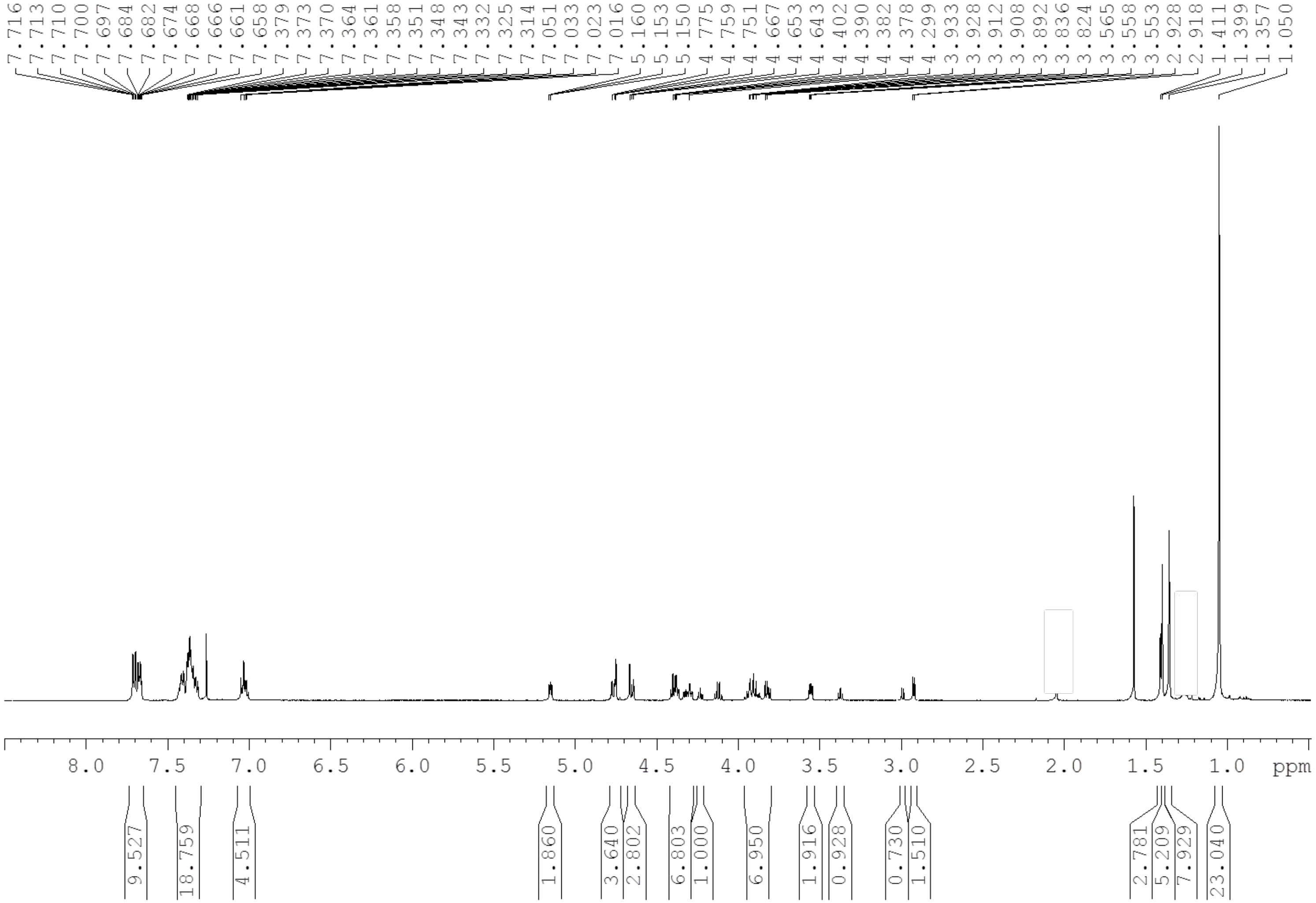

6-*O*-*tert*-Butyldiphenylsilyl-2-*O*-(*p*-fluorobenzyl)-3,4-*O*-isopropylidene-α,β-D-galactopyranoside, (**8(α,β)**)

^{13}C-NMR (125 MHz, $CDCl_3$)

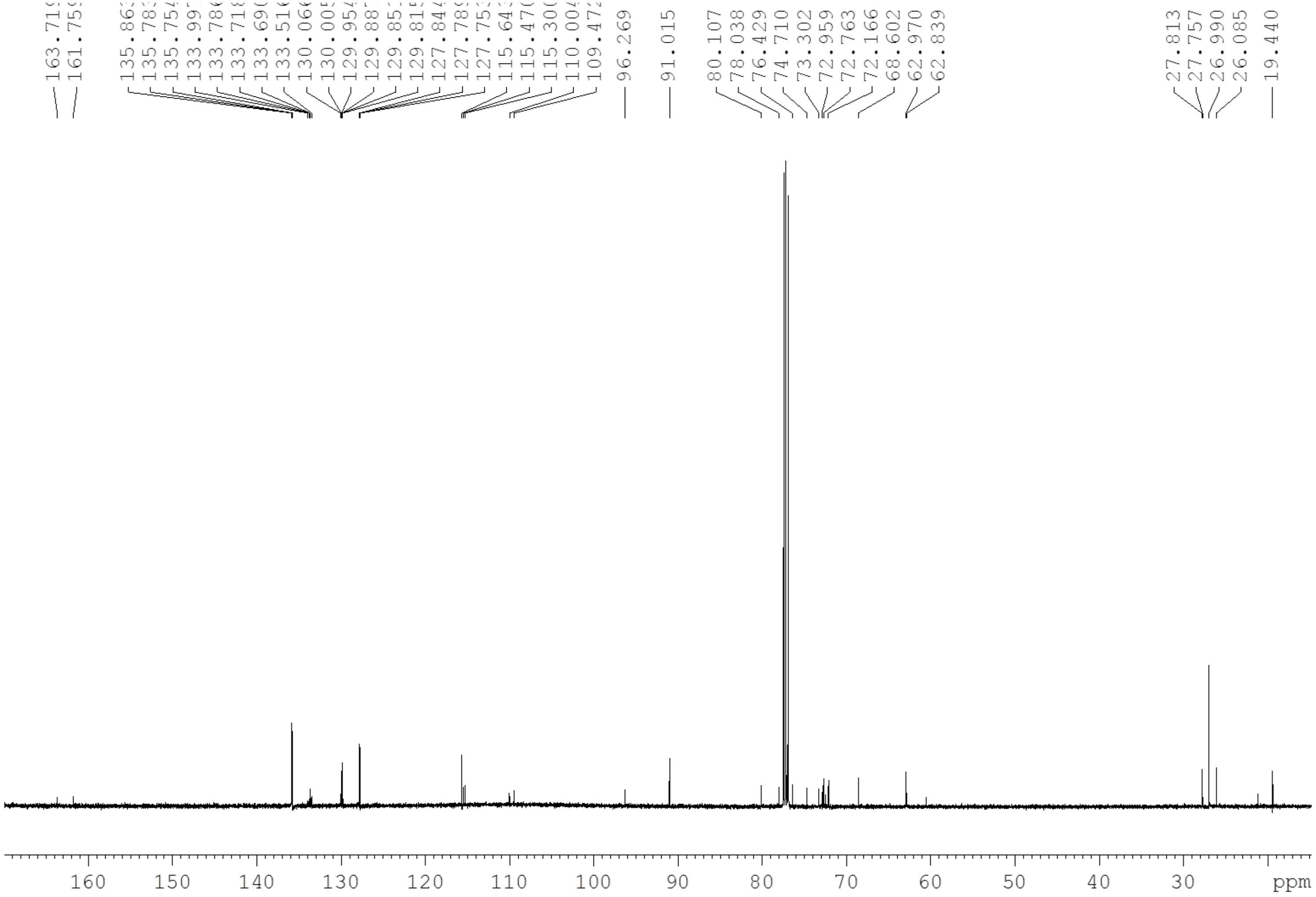

(*R*)-{1-[3,5-bis-(trifluoromethyl)phenyl]ethyl} 6-*O*-*tert*-butyldiphenylsilyl-2-*O*-*p*-fluorobenzyl-3,4-*O*-isopropylidene-α-D-galactopyranoside, (11α)

^{1}H-NMR (500 MHz, $CDCl_3$)

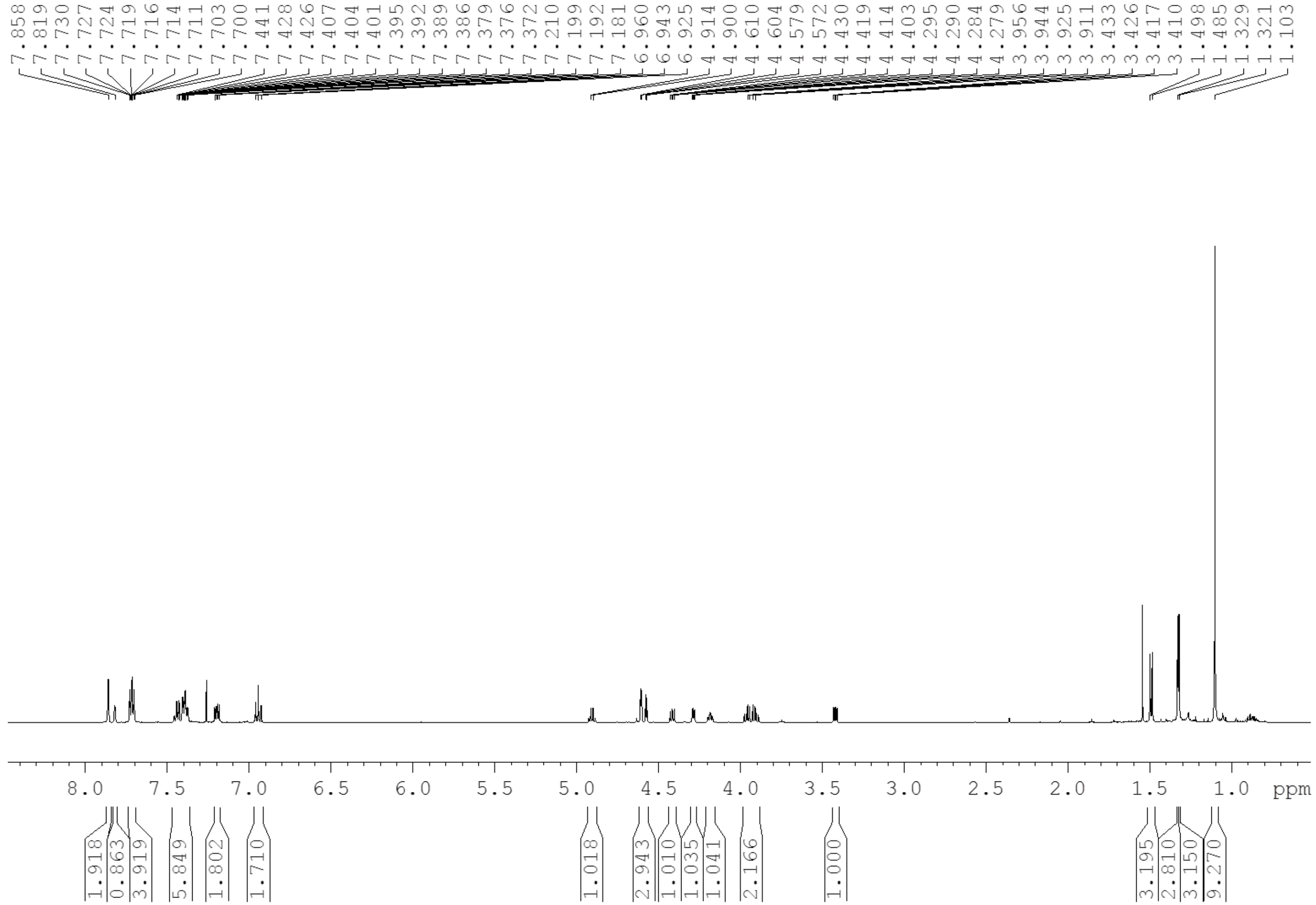

(*R*)-{1-[3,5-bis-(trifluoromethyl)phenyl]ethyl} 6-*O*-*tert*-butyldiphenylsilyl-2-*O*-*p*-fluorobenzyl-3,4-*O*-isopropylidene-α-D-galactopyranoside, (11α)

^{13}C-NMR (125 MHz, $CDCl_3$)

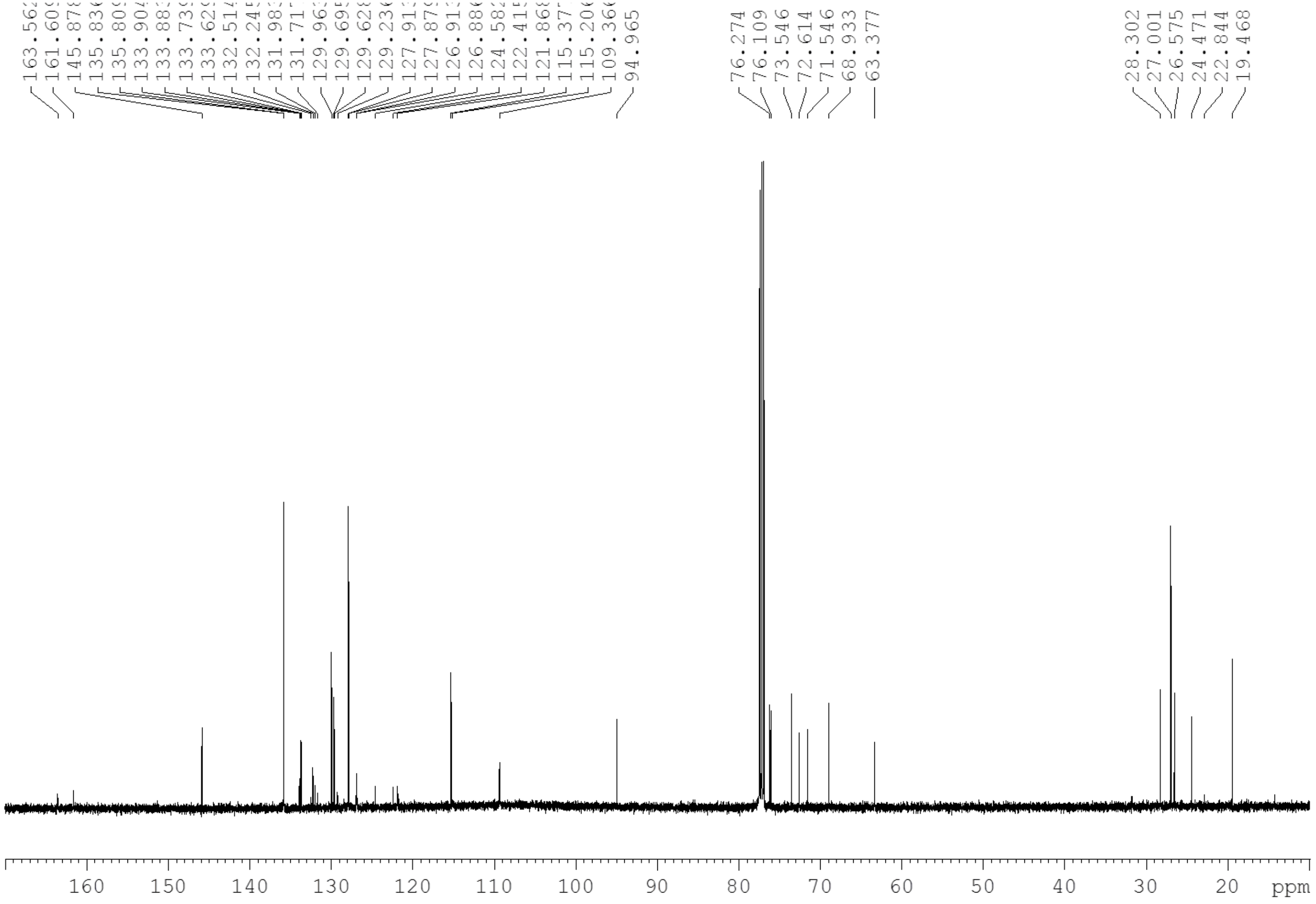

(*R*)-{1-[3,5-bis-(trifluoromethyl)phenyl]ethyl} 6-*O*-*tert*-butyldiphenylsilyl-2-*O*-*p*-fluorobenzyl-3,4-*O*-isopropylidene-β-D-galactopyranoside, (11β)

^{1}H-NMR (500 MHz, $CDCl_3$)

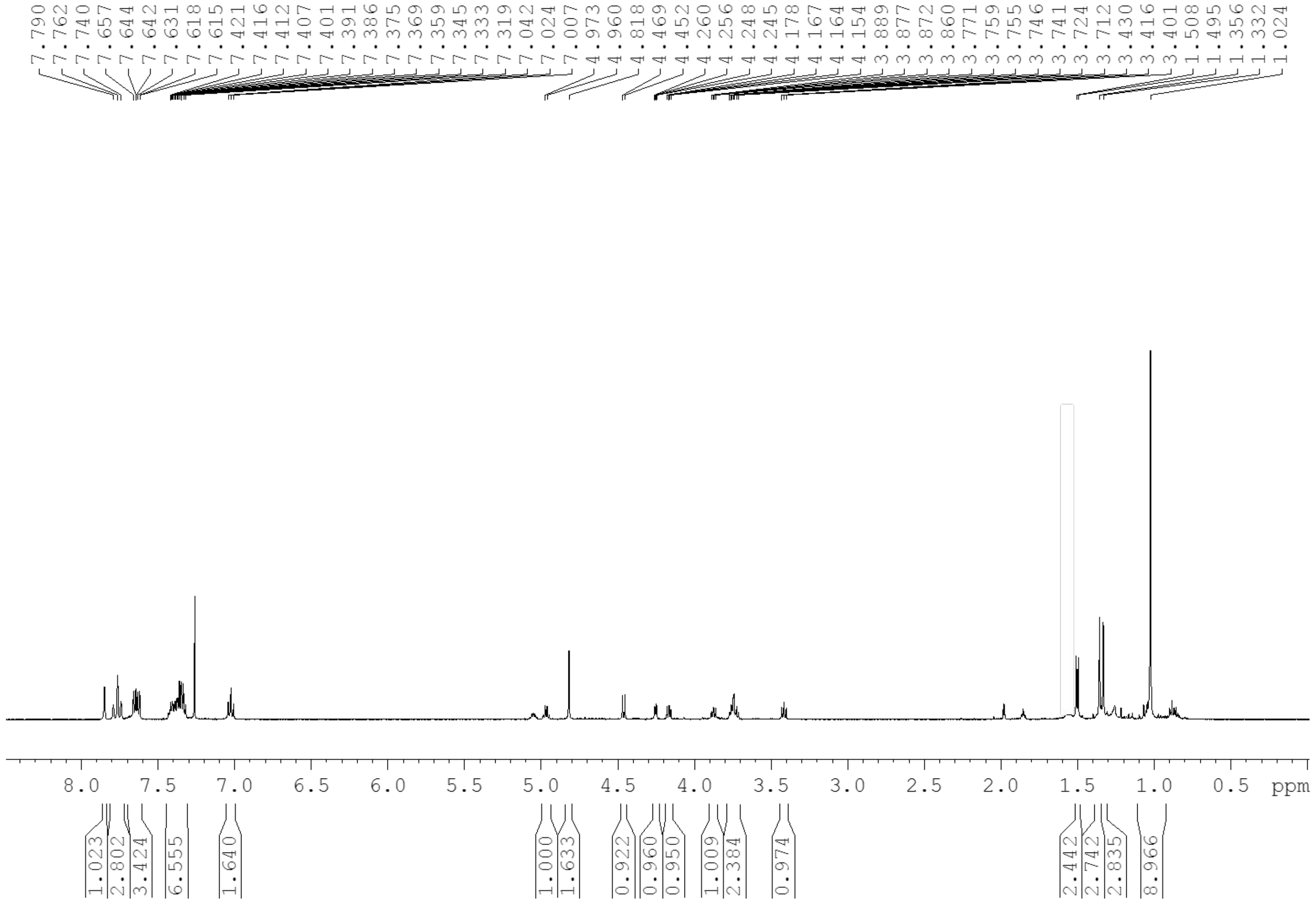

(*R*)-{1-[3,5-bis-(trifluoromethyl)phenyl]ethyl} 6-*O*-*tert*-butyldiphenylsilyl-2-*O*-*p*-fluorobenzyl-3,4-*O*-isopropylidene-β-D-galactopyranoside, (11β)

^{13}C-NMR (125 MHz, $CDCl_3$)

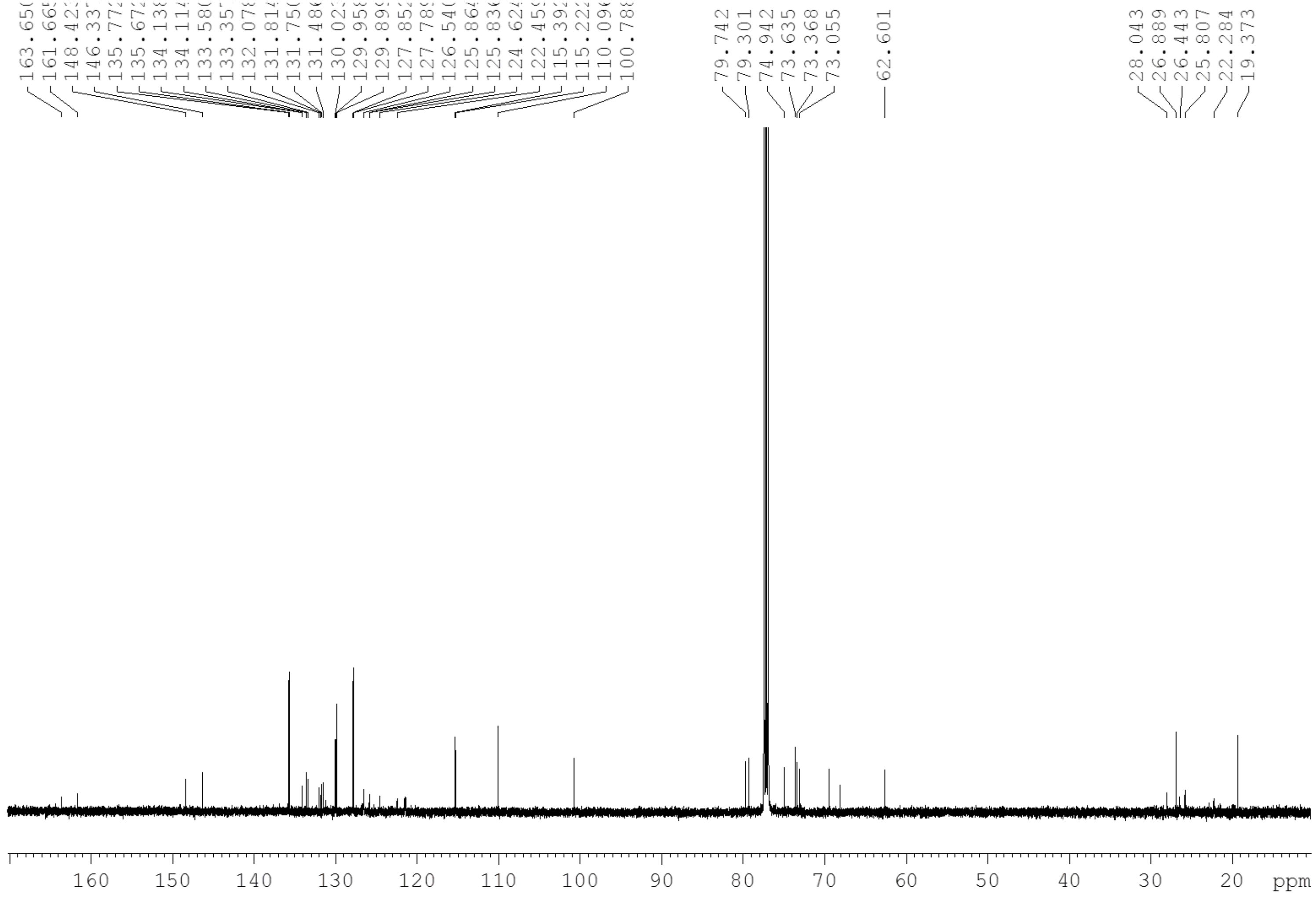

(*R*)-{1-[3,5-bis-(trifluoromethyl)phenyl]ethyl} 2-*O*-*p*-fluorobenzyl-3,4-*O*-isopropylidene-α-D-galactopyranoside, (**12α**)

^{1}H-NMR (500 MHz, $CDCl_3$)

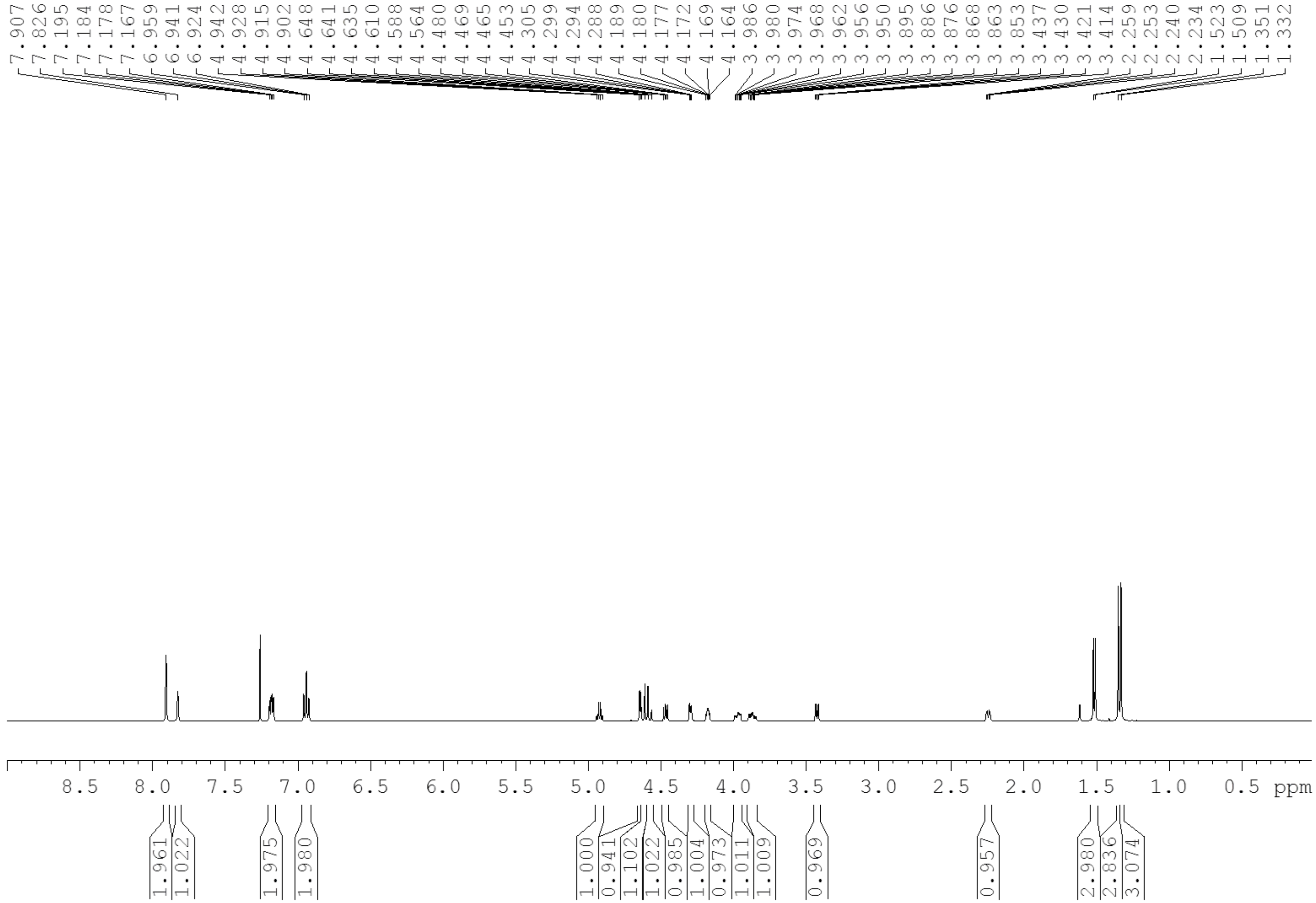

(*R*)-{1-[3,5-bis-(trifluoromethyl)phenyl]ethyl} 2-*O*-*p*-fluorobenzyl-3,4-*O*-isopropylidene-α-D-galactopyranoside, (**12α**)

^{13}C-NMR (125 MHz, CDCl$_3$)

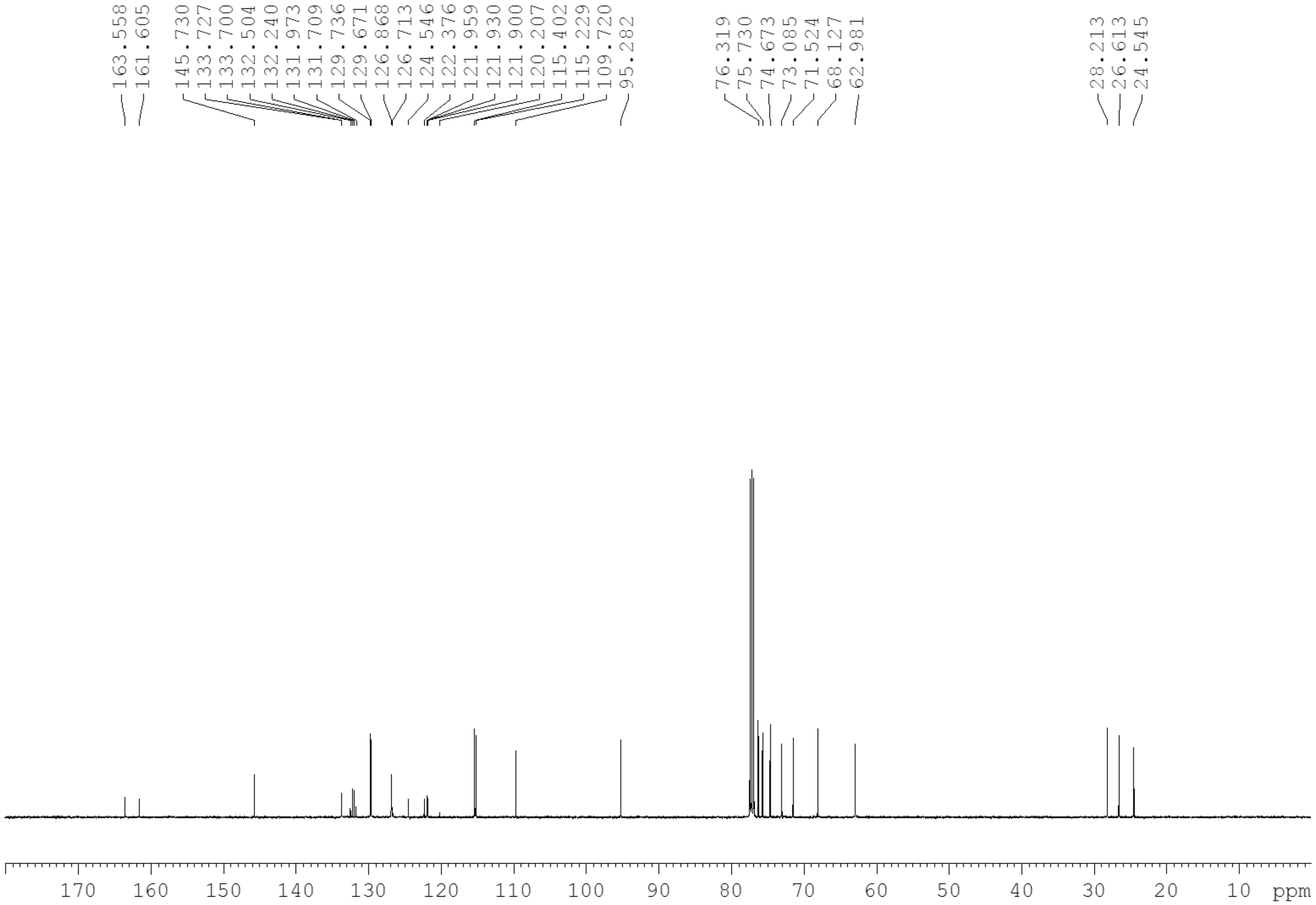

(*R*)-{1-[3,5-bis-(trifluoromethyl)phenyl]ethyl} 2-*O*-*p*-fluorobenzyl-3,4-*O*-isopropylidene-β-D-galactopyranoside, (**12β**)

^{1}H-NMR (500 MHz, $CDCl_3$)

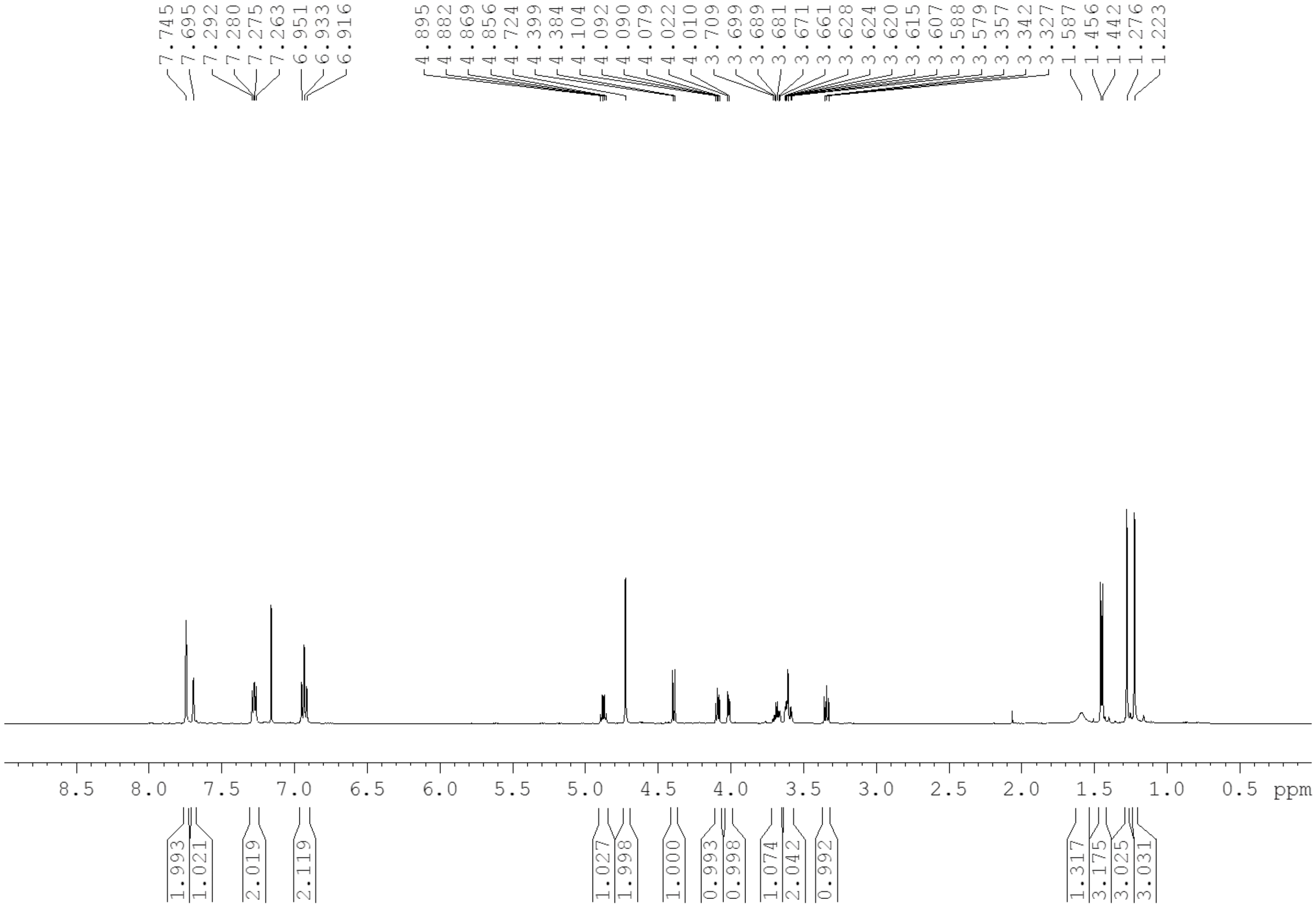

(*R*)-{1-[3,5-bis-(trifluoromethyl)phenyl]ethyl} 2-*O*-*p*-fluorobenzyl-3,4-*O*-isopropylidene-β-D-galactopyranoside, (**12β**)

^{13}C-NMR (125 MHz, CDCl$_3$)

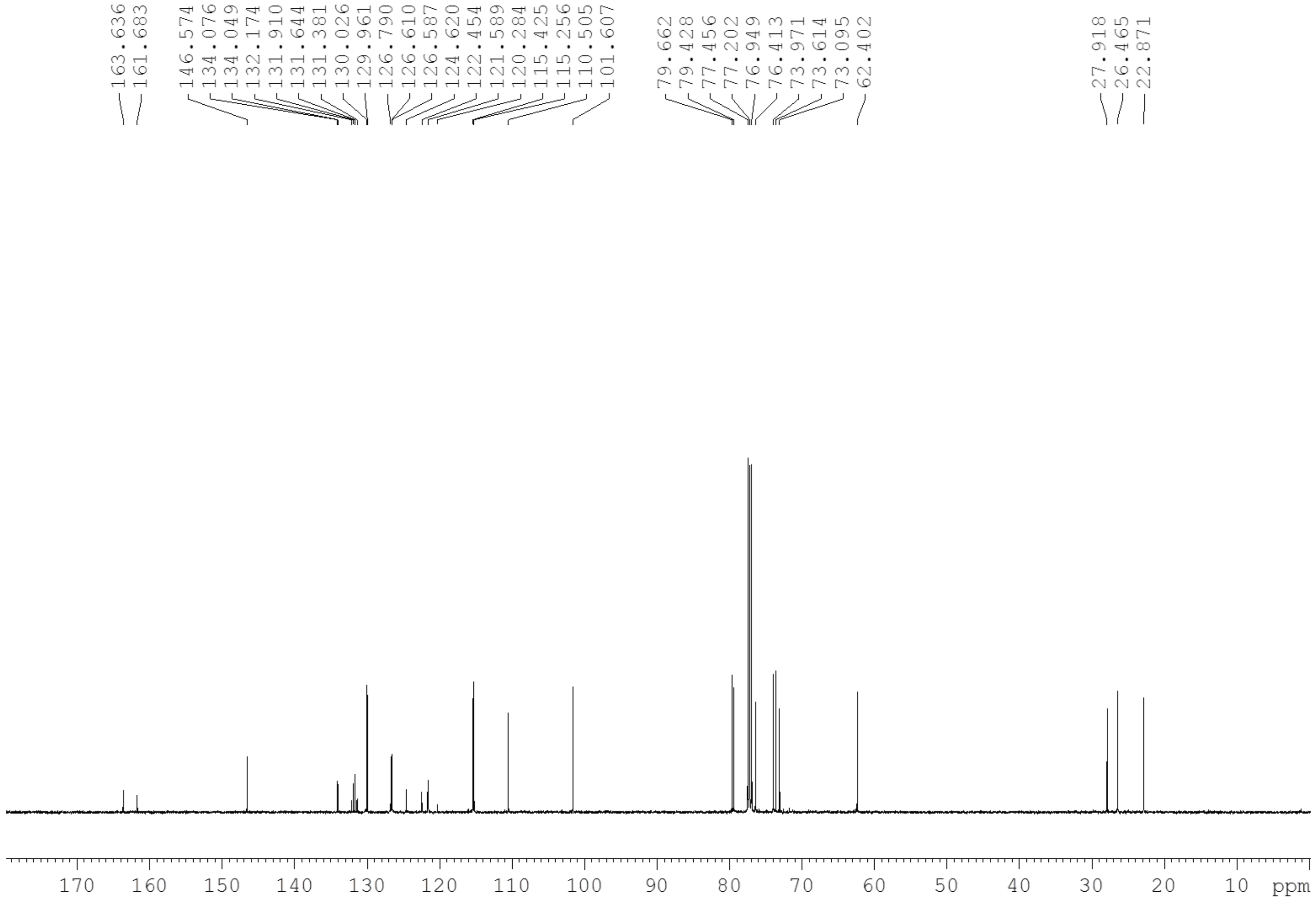

(*R*)-{1-[3,5-bis-(trifluoromethyl)phenyl]ethyl} 2-*O*-*p*-fluorobenzyl-α-D-galactopyranoside, (**13α**)

^{1}H-NMR (500 MHz, $CDCl_3$)

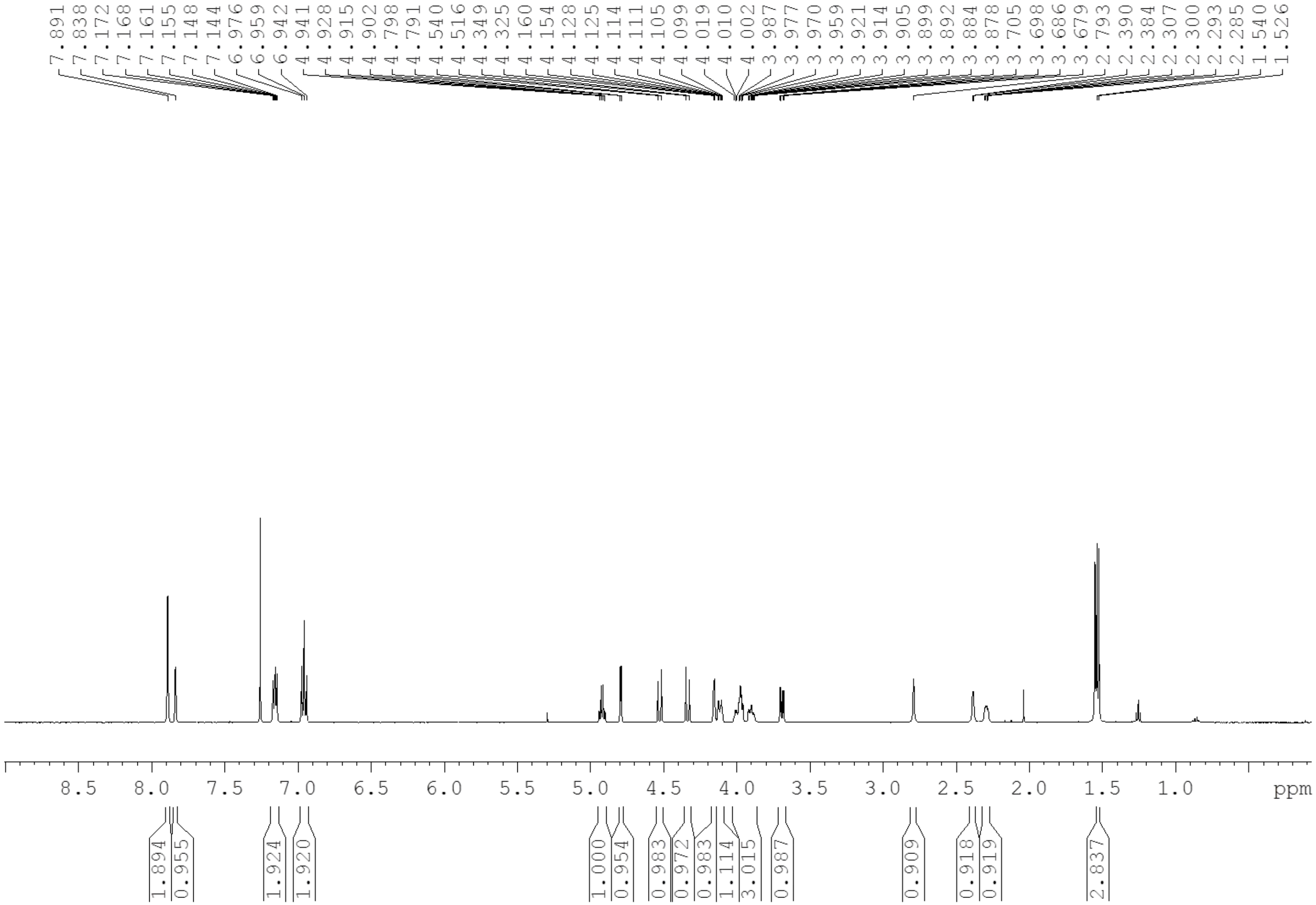

(*R*)-{1-[3,5-bis-(trifluoromethyl)phenyl]ethyl} 2-*O*-*p*-fluorobenzyl-α-D-galactopyranoside, (**13α**)

^{13}C-NMR (125 MHz, CDCl$_3$)

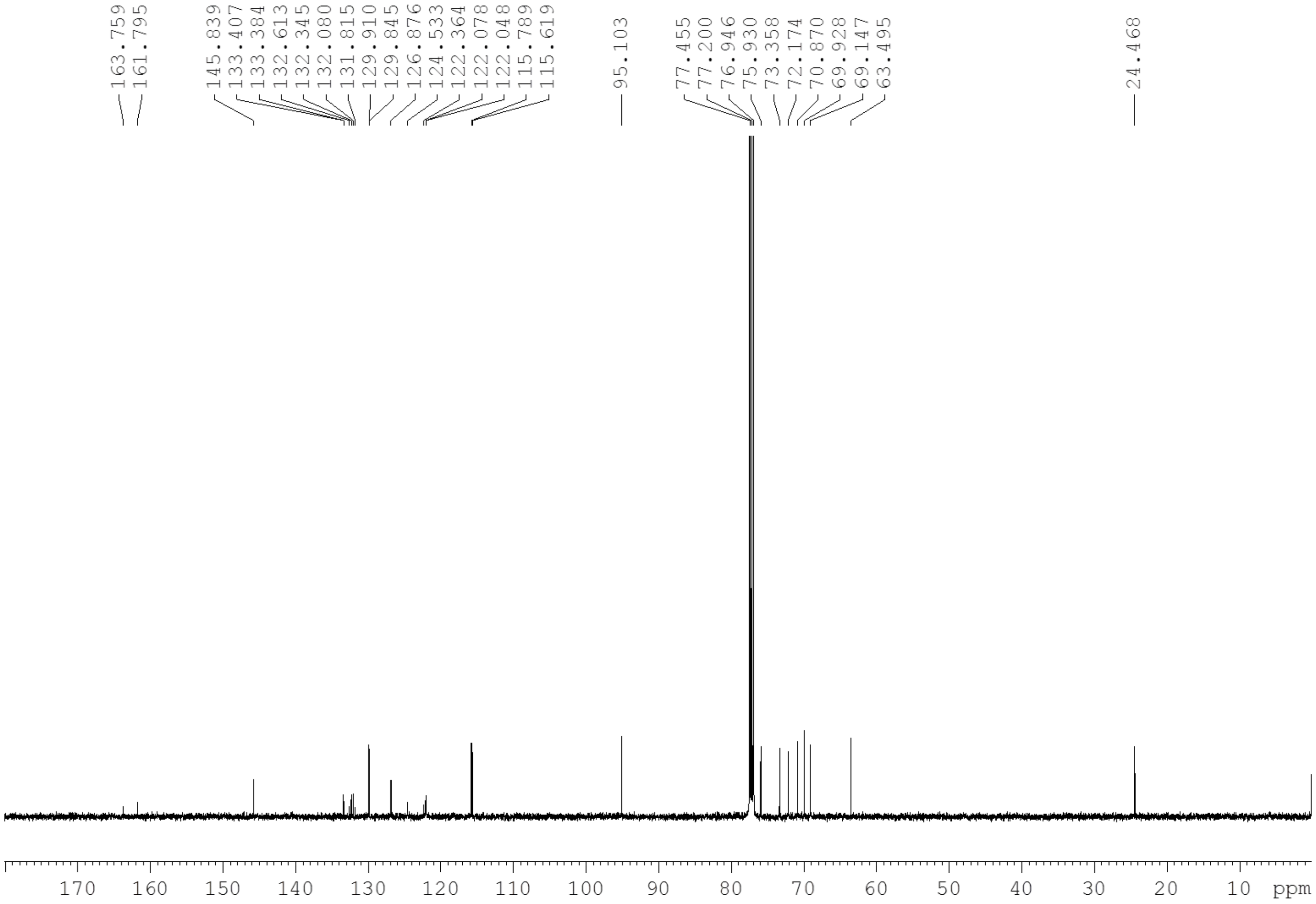

(*R*)-{1-[3,5-bis-(trifluoromethyl)phenyl]ethyl} 2-*O*-*p*-fluorobenzyl-β-D-galactopyranoside, (13β)

^{1}H-NMR (500 MHz, $CDCl_3$)

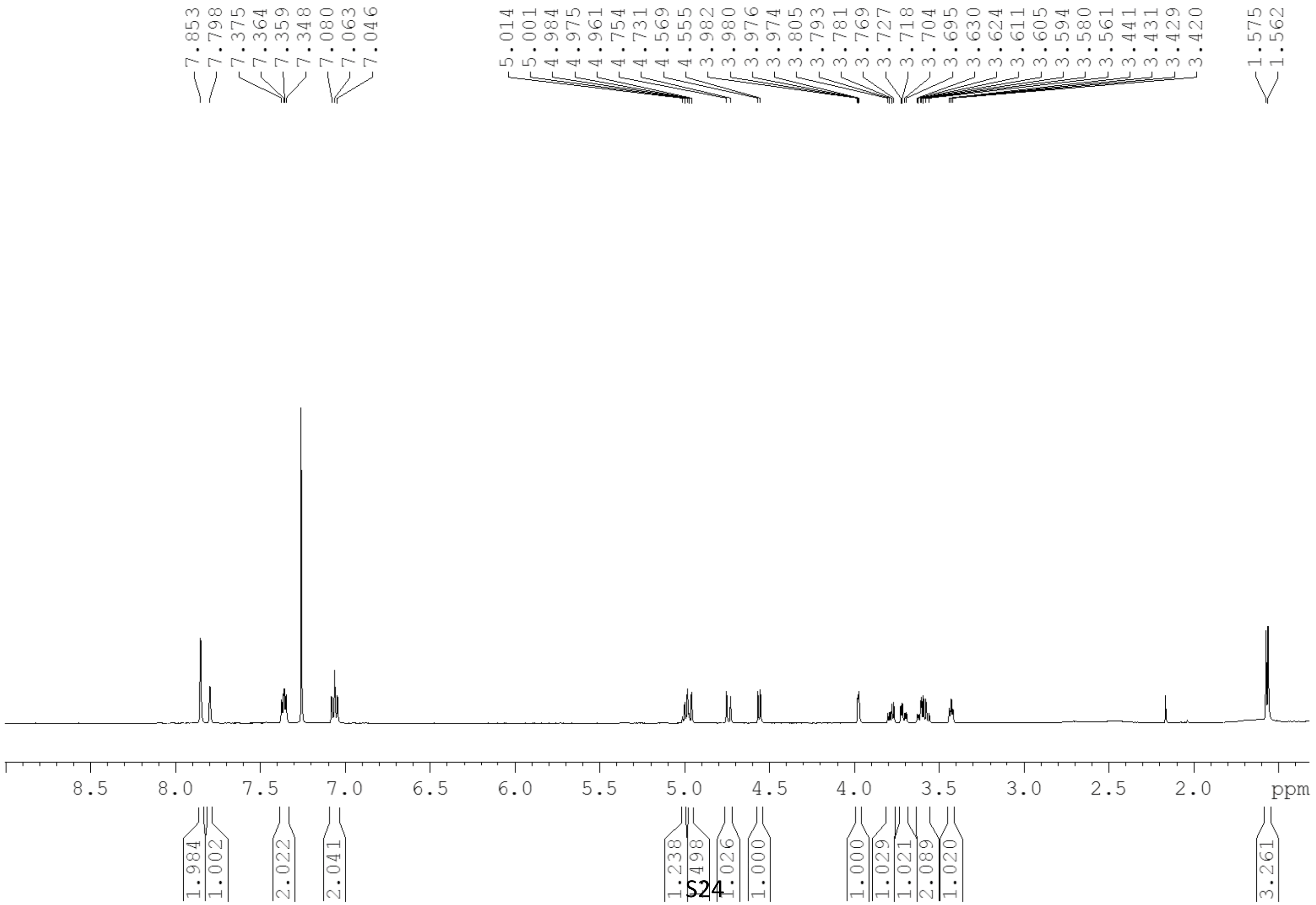

(*R*)-{1-[3,5-bis-(trifluoromethyl)phenyl]ethyl} 2-*O*-*p*-fluorobenzyl-β-D-galactopyranoside, (**13β**)

^{13}C-NMR (125 MHz, $CDCl_3$)

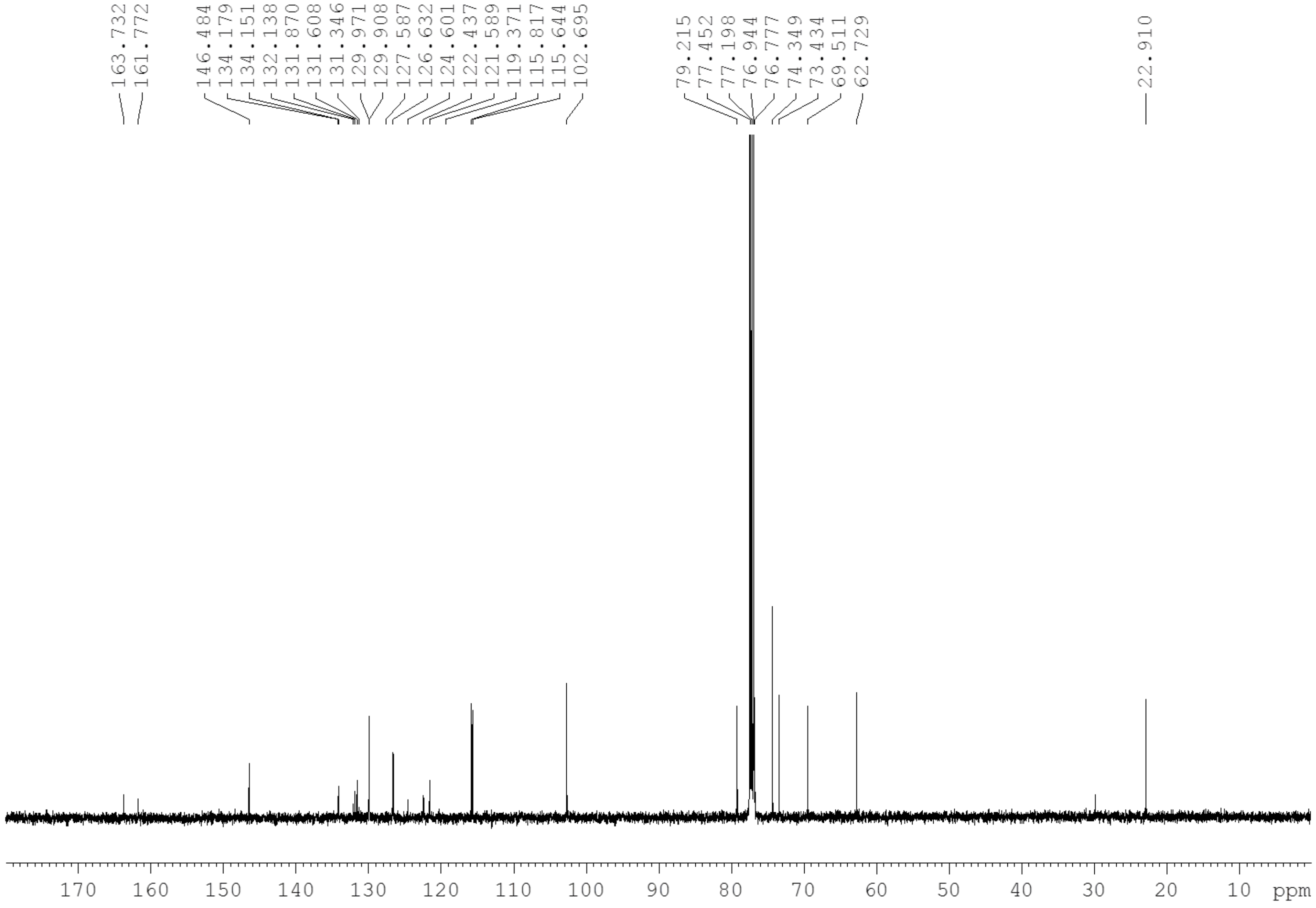

(*R*)-{1-[3,5-bis-(trifluoromethyl)phenyl]ethyl} (*R*)-(4,6-*O*-benzylidene)-2-*O*-*p*-fluorobenzyl-α-D-galactopyranoside, (14α)

^{1}H-NMR (500 MHz, $CDCl_3$)

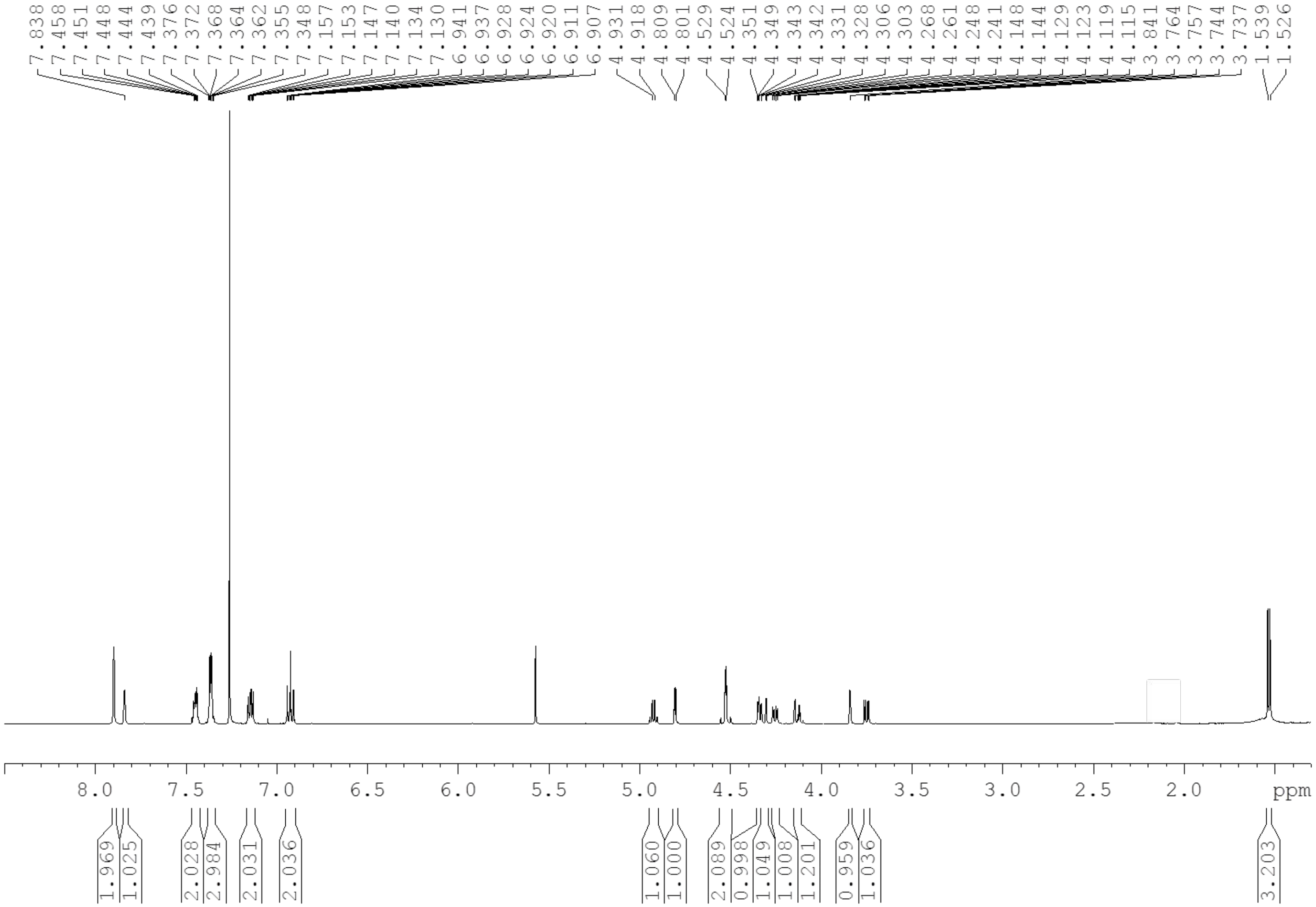

(*R*)-{1-[3,5-bis-(trifluoromethyl)phenyl]ethyl} (*R*)-(4,6-*O*-benzylidene)-2-*O*-*p*-fluorobenzyl-α-D-galactopyranoside, (14α)

^{13}C-NMR (125 MHz, $CDCl_3$)

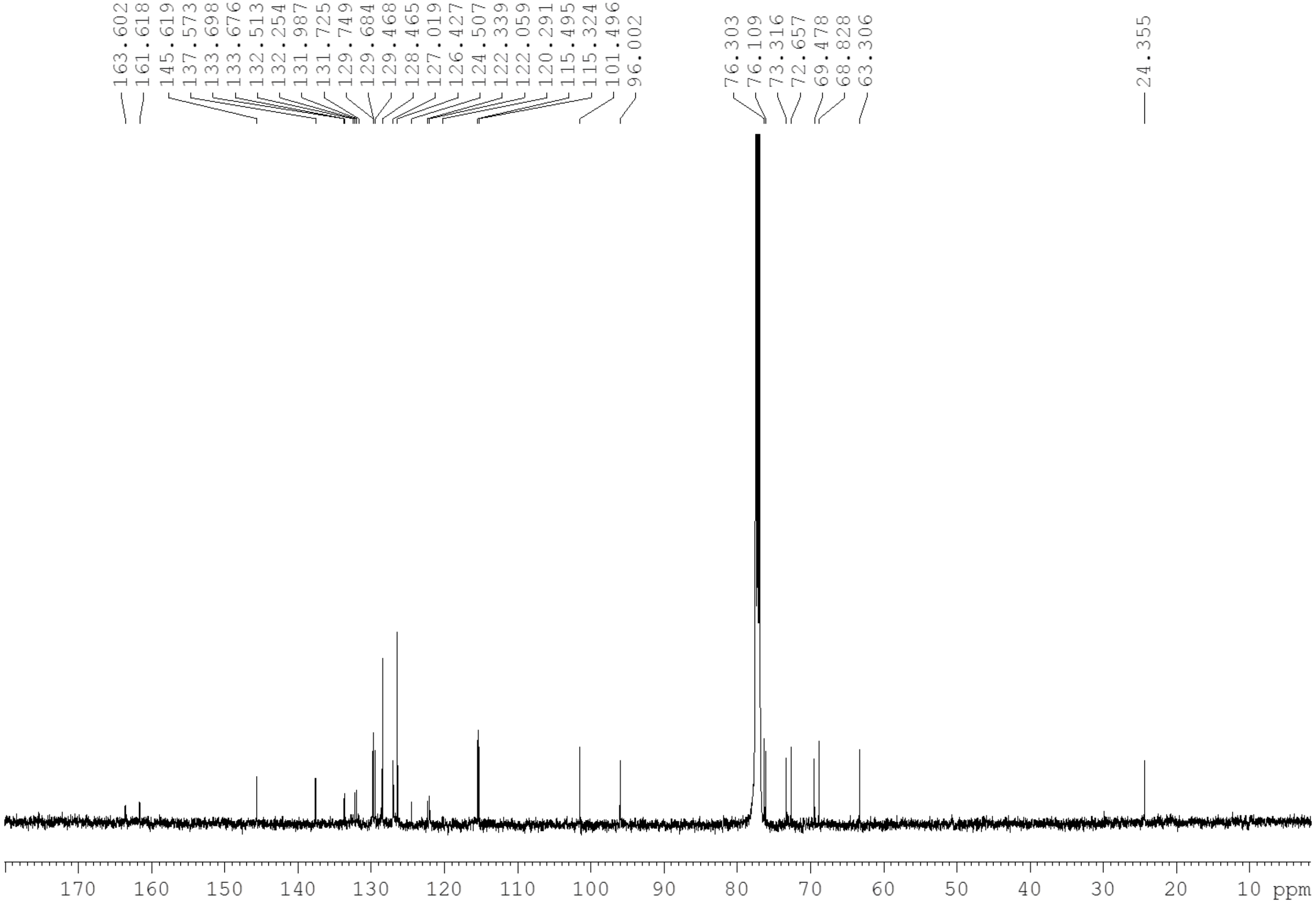

(*R*)-{1-[3,5-bis-(trifluoromethyl)phenyl]ethyl} (*R*)-(4,6-*O*-benzylidene)- 2-*O*-*p*-fluorobenzyl-β-D-galactopyranoside, (14β)

^{1}H-NMR (500 MHz, $CDCl_3$)

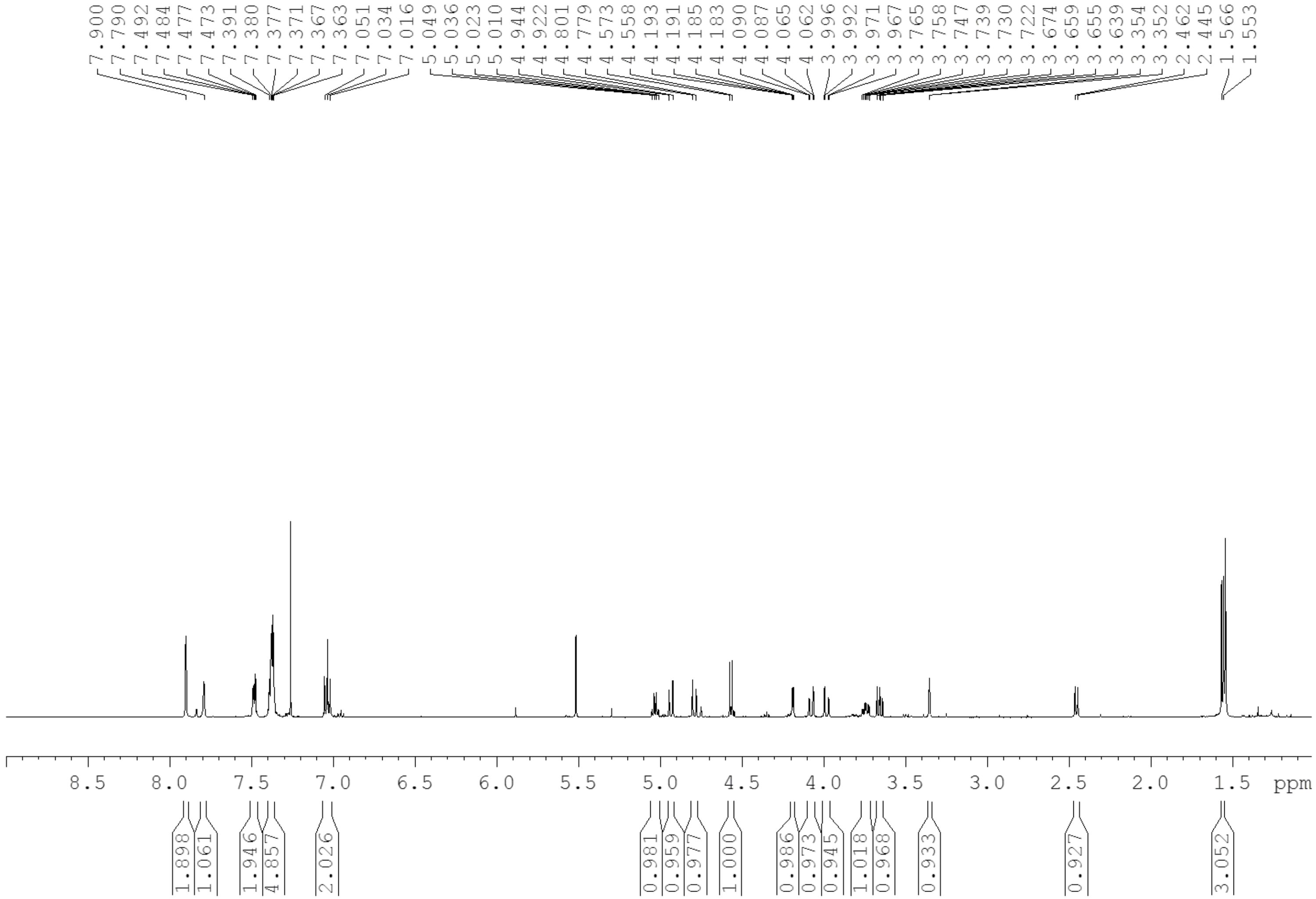

(*R*)-{1-[3,5-bis-(trifluoromethyl)phenyl]ethyl} (*R*)-(4,6-*O*-benzylidene)- 2-*O*-*p*-fluorobenzyl-β-D-galactopyranoside, (14β)

^{13}C-NMR (125 MHz, $CDCl_3$)

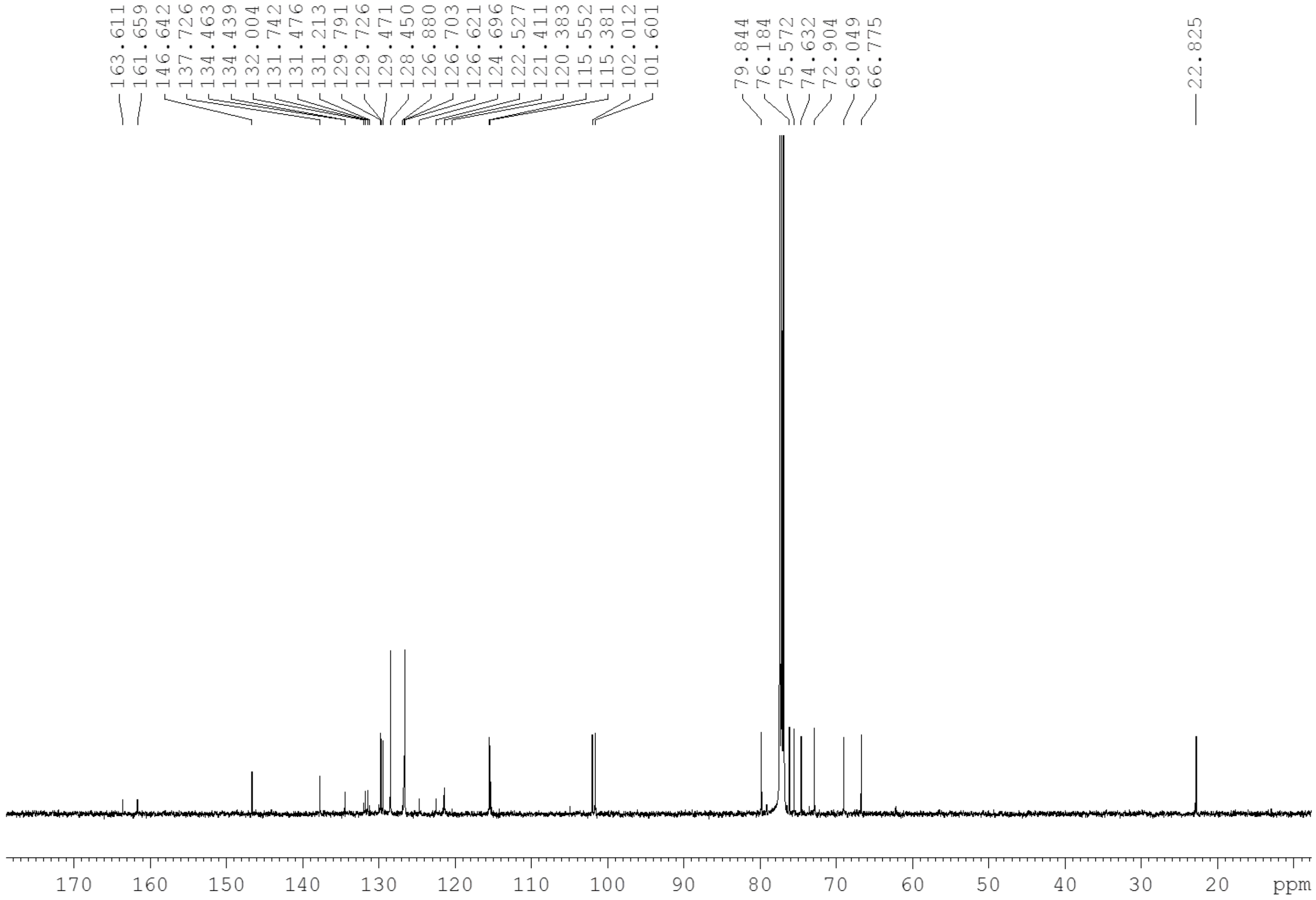

Phenyl 2,3,4-tri-*O*-acetyl-1-thio-α-L-arabinopyranoside, (**16α**)

^{1}H-NMR (500 MHz, $CDCl_3$)

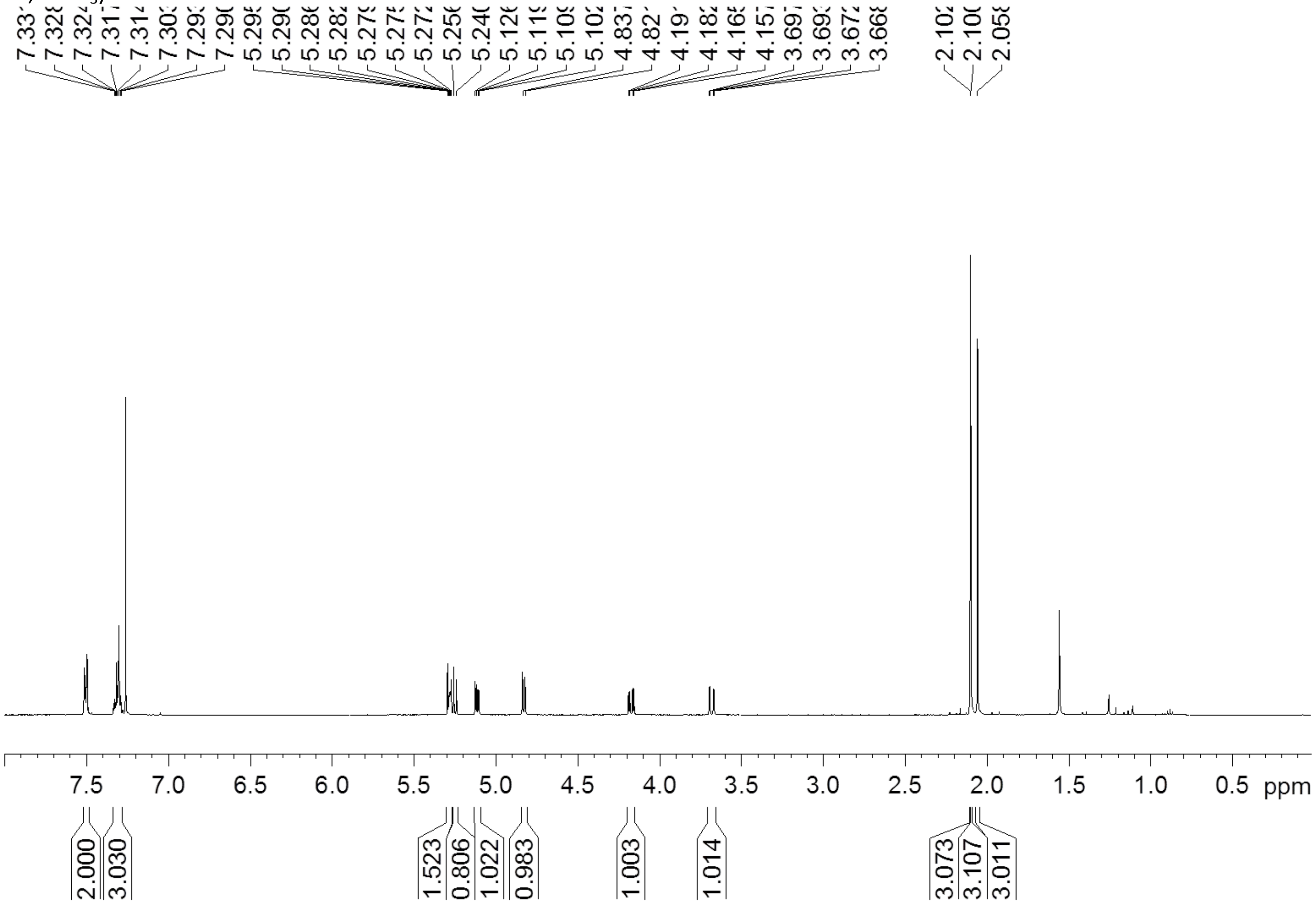

Phenyl 2,3,4-tri-*O*-acetyl-1-thio-α-L-arabinopyranoside, (**16α**)

^{13}C-NMR (125 MHz, $CDCl_3$)

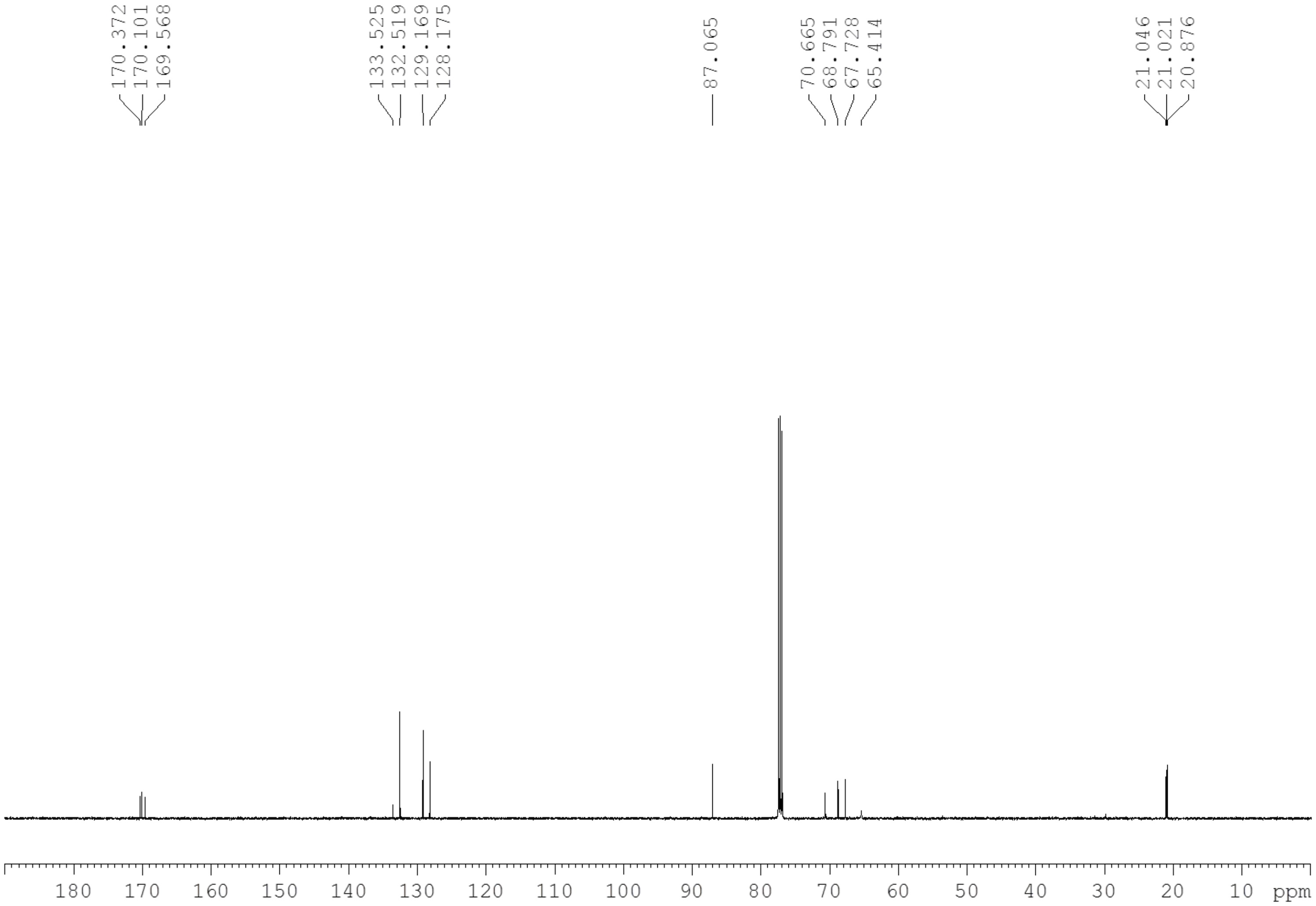

Phenyl 1-thio-α-L-arabinopyranoside, (**17α**)

^{1}H-NMR (500 MHz, $CDCl_3$)

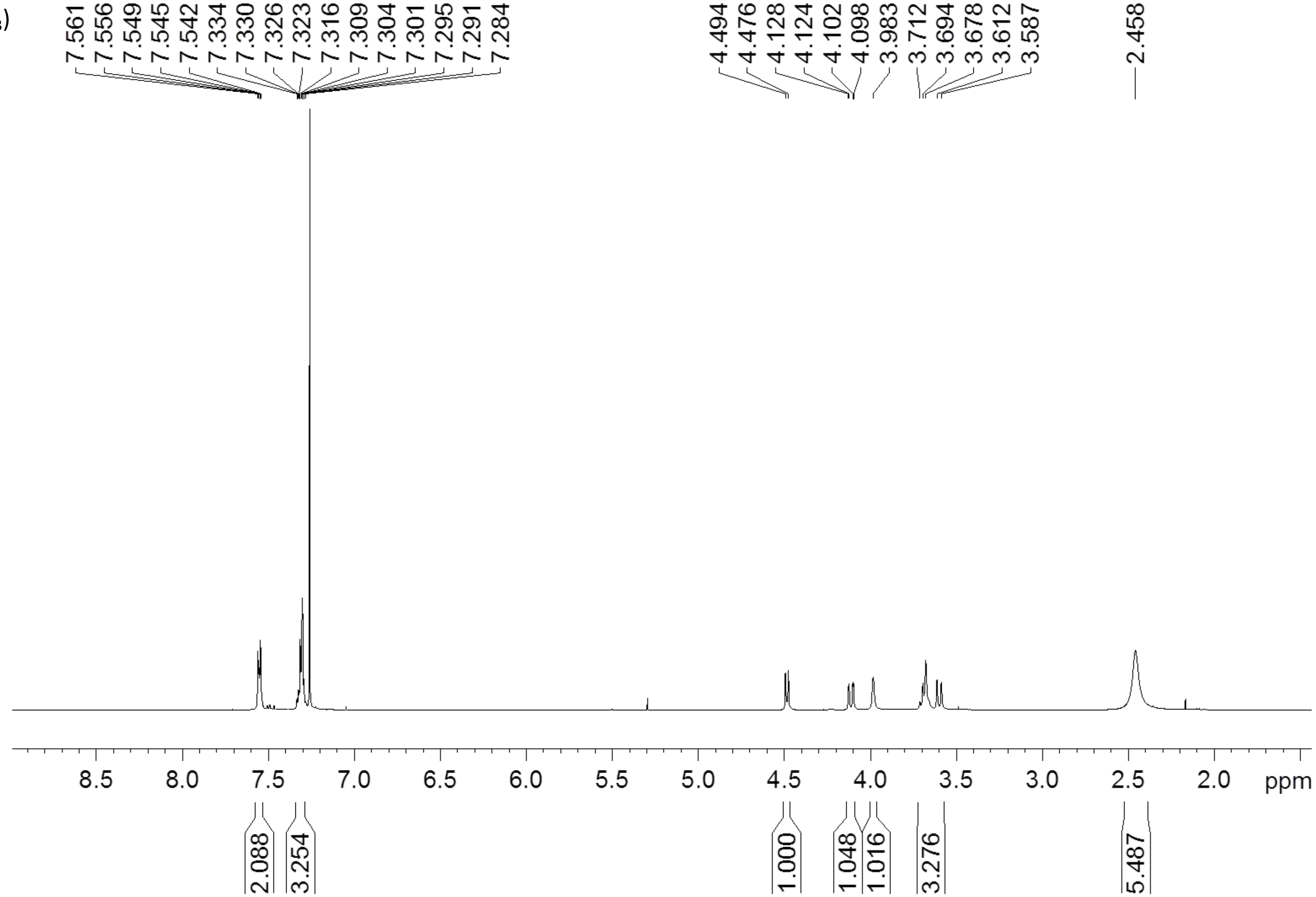

Phenyl 1-thio-α-L-arabinopyranoside, (**17α**)

^{13}C-NMR (125 MHz, $CDCl_3$)

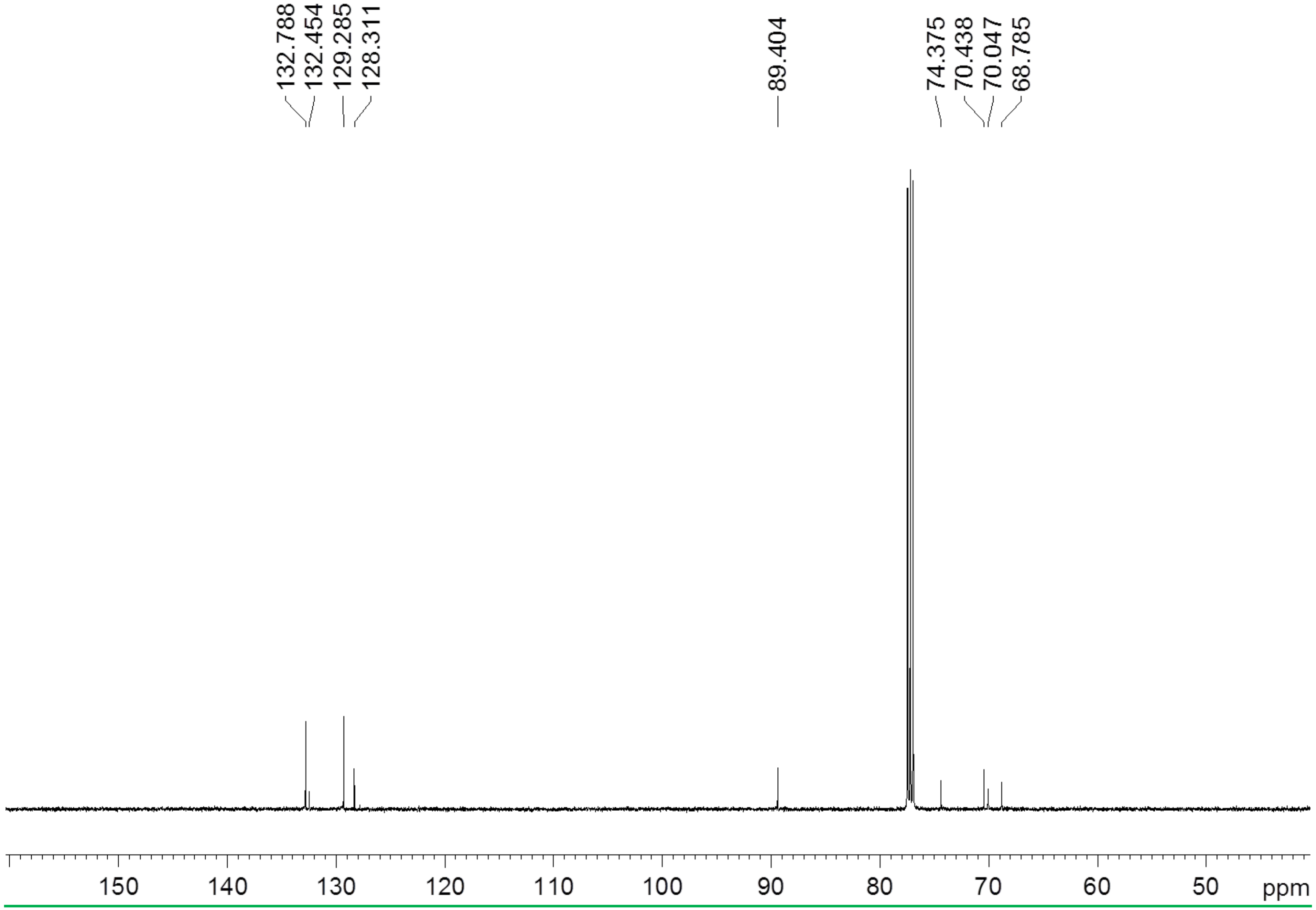

Phenyl 3,4-*O*-isopropylidene-1-thio-α-L-arabinopyranoside, (**18α**)

^{1}H-NMR (500 MHz, $CDCl_3$)

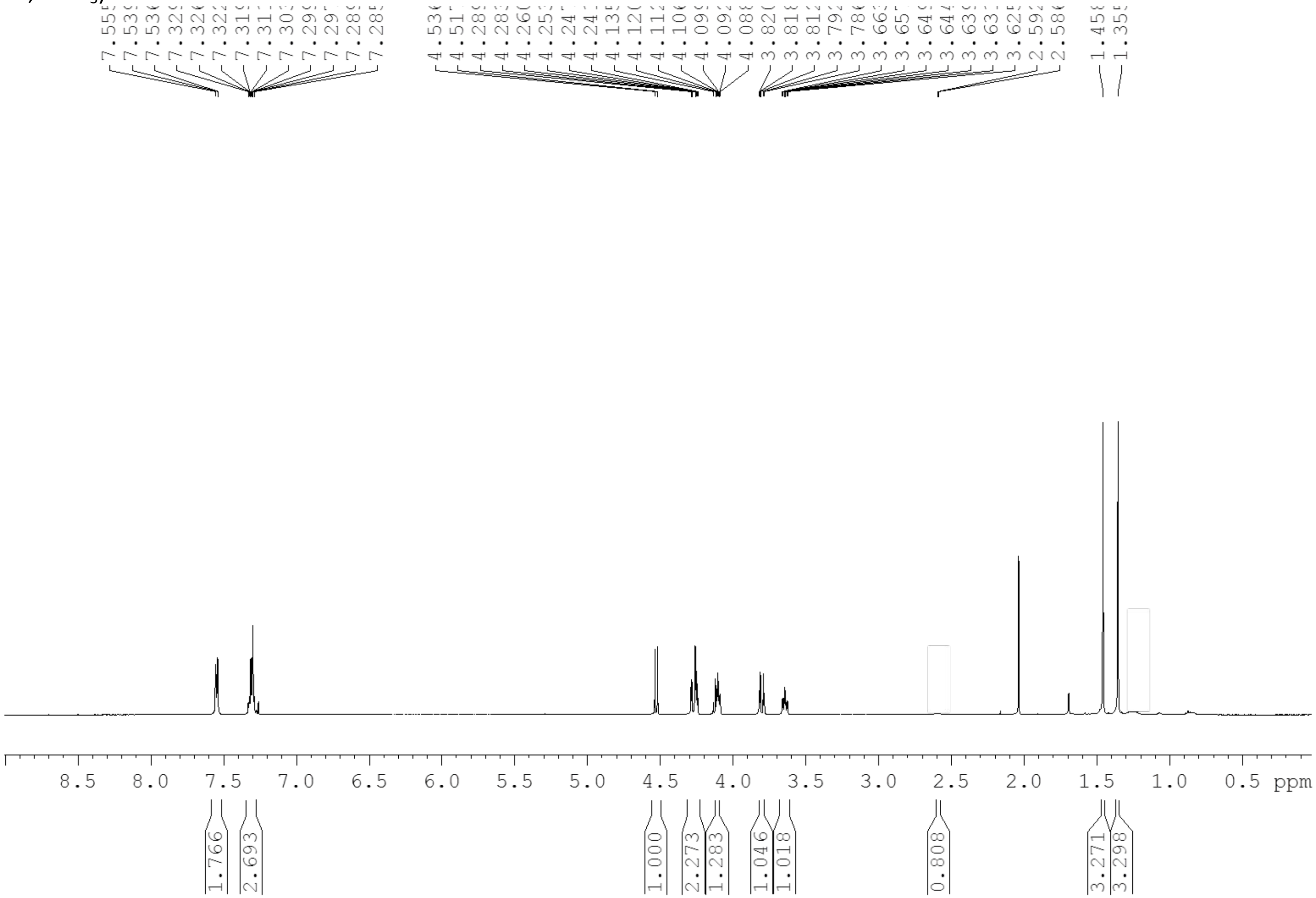

Phenyl 3,4-*O*-isopropylidene-1-thio-α-L-arabinopyranoside, (**18α**)

^{13}C-NMR (125 MHz, $CDCl_3$)

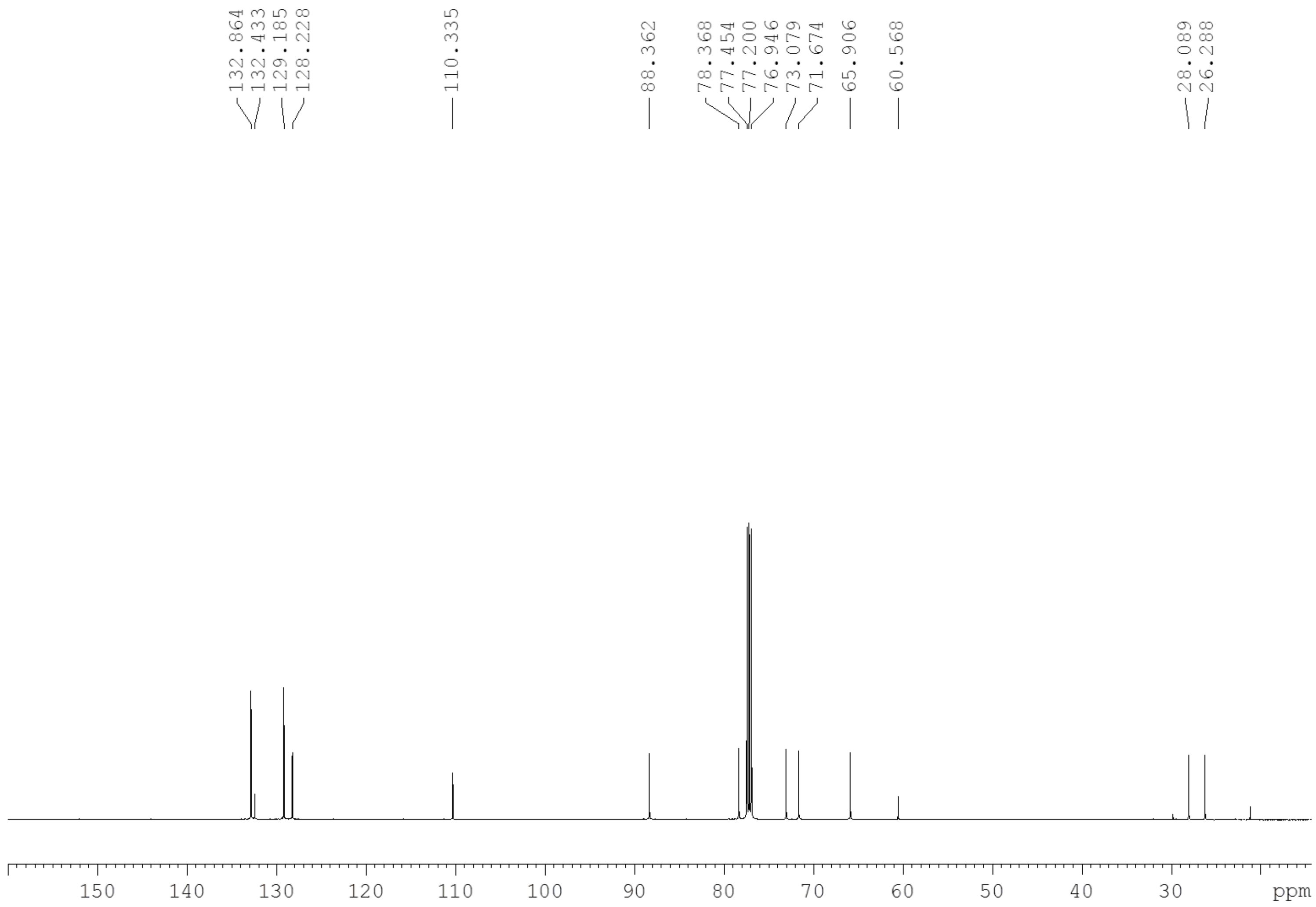

Phenyl 2-*O*-(*p*-fluorobenzyl)-3,4-*O*-isopropylidene-1-thio-α-L-arabinopyranoside, **(19α)**

^{1}H-NMR (500 MHz, $CDCl_3$)

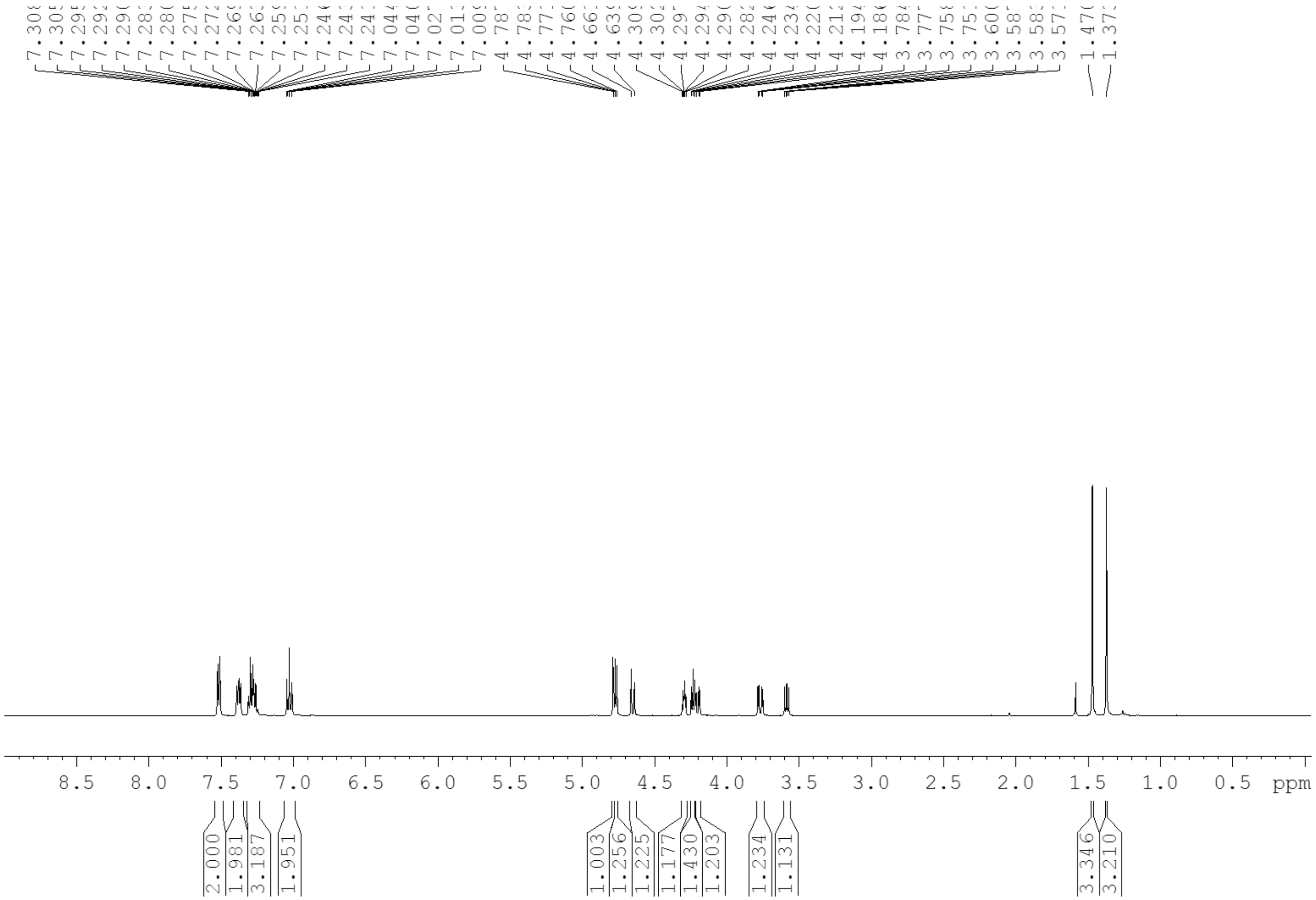

Phenyl 2-*O*-(*p*-fluorobenzyl)-3,4-*O*-isopropylidene-1-thio-α-L-arabinopyranoside, (**19α**)

^{13}C-NMR (125 MHz, $CDCl_3$)

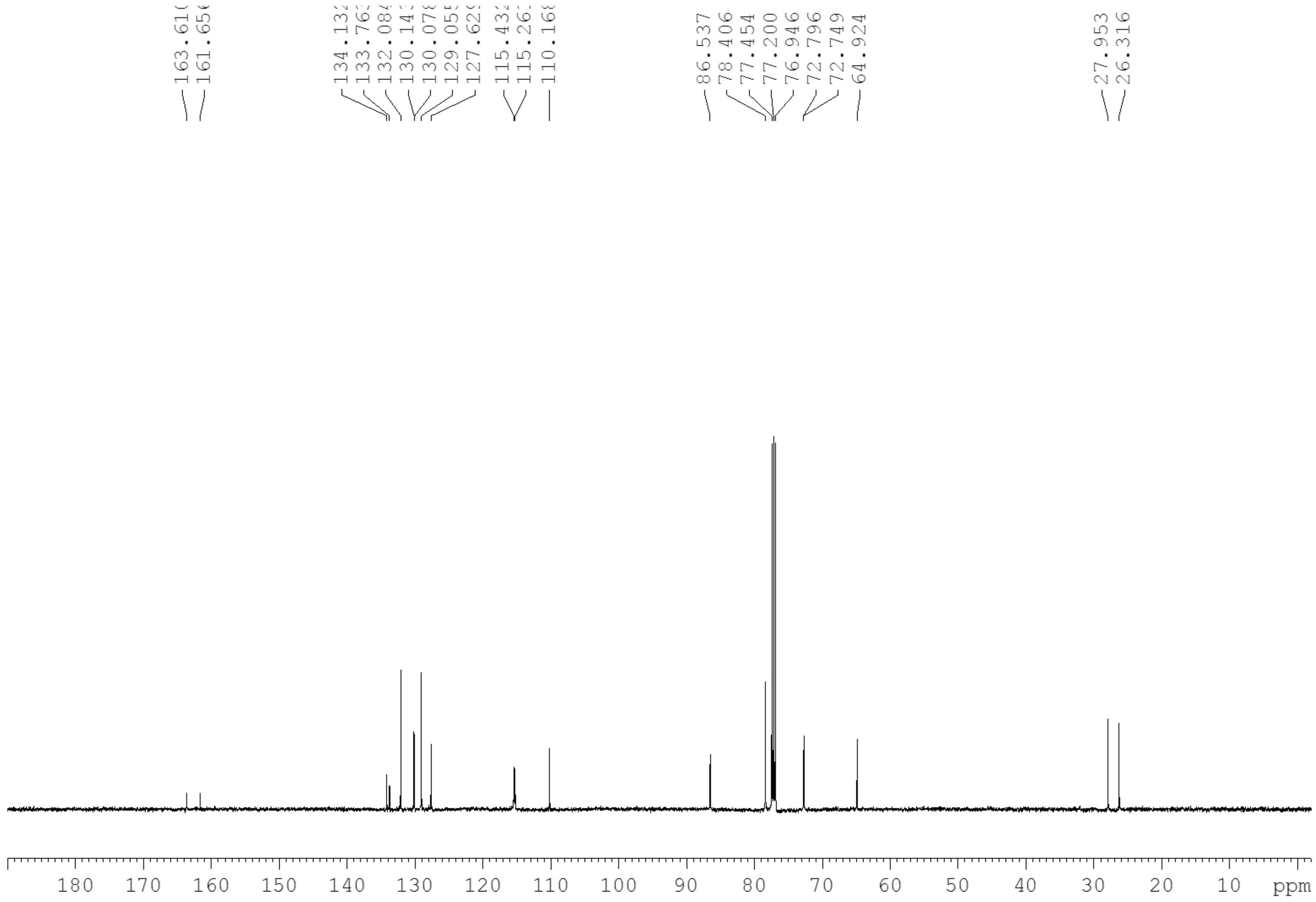

2-*O*-(*p*-fluorobenzyl)-3,4-*O*-isopropylidene-α,β-L-arabinopyranoside, (20(α,β))

^{1}H-NMR (500 MHz, $CDCl_3$)

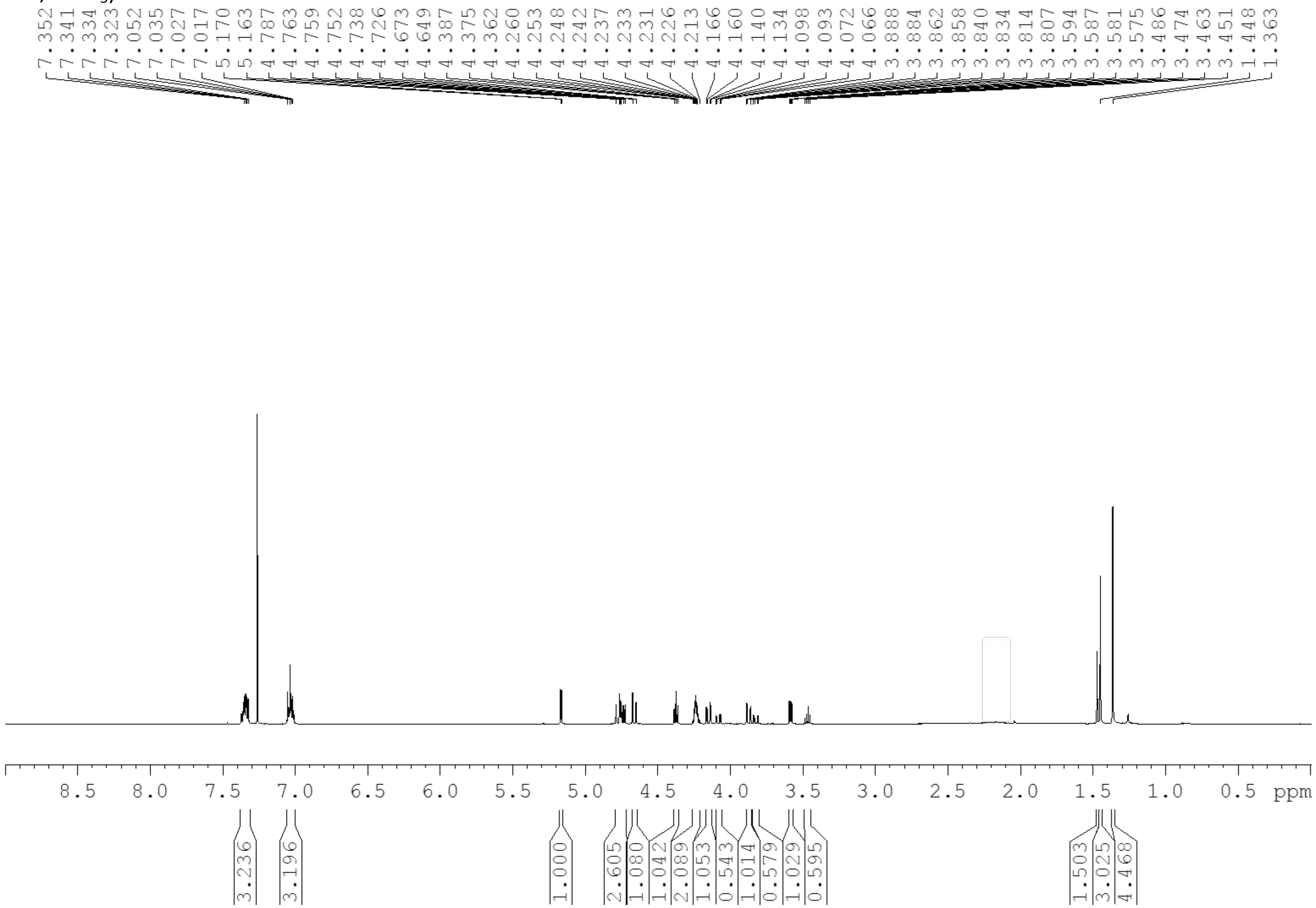

2-*O*-(*p*-fluorobenzyl)-3,4-*O*-isopropylidene-α,β-L-arabinopyranoside, (20(α,β))

^{13}C-NMR (125 MHz, $CDCl_3$)

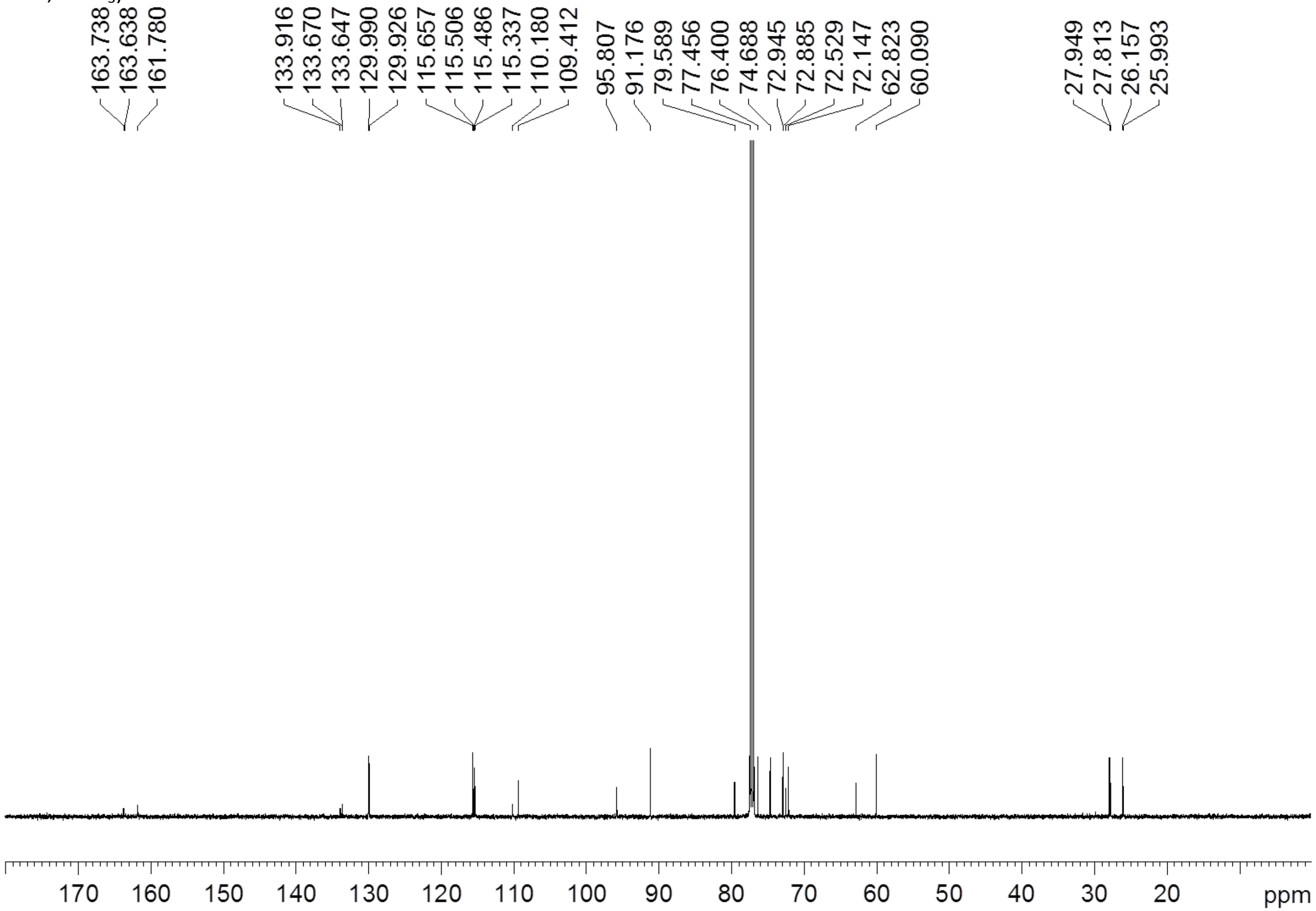

(*R*)-{1-[3,5-bis-(trifluoromethyl)phenyl]ethyl} 2-*O*-*p*-fluorobenzyl-3,4-*O*-isopropylidene-α-L-arabinopyranoside, (22α)

^{1}H-NMR (500 MHz, $CDCl_3$)

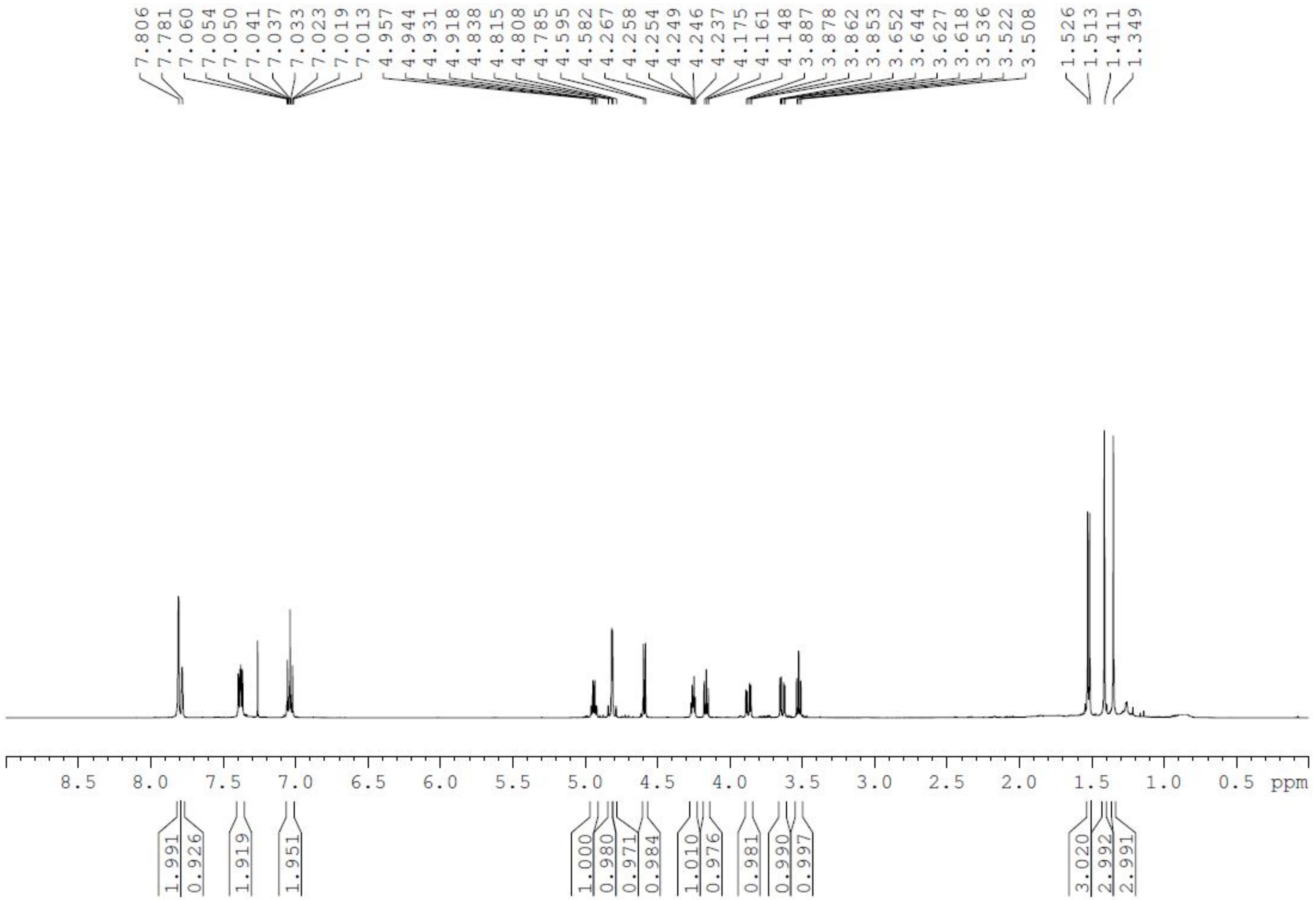

(*R*)-{1-[3,5-bis-(trifluoromethyl)phenyl]ethyl} 2-*O*-*p*-fluorobenzyl-3,4-*O*-isopropylidene-α-L-arabinopyranoside, (22α)

^{13}C-NMR (125 MHz, $CDCl_3$)

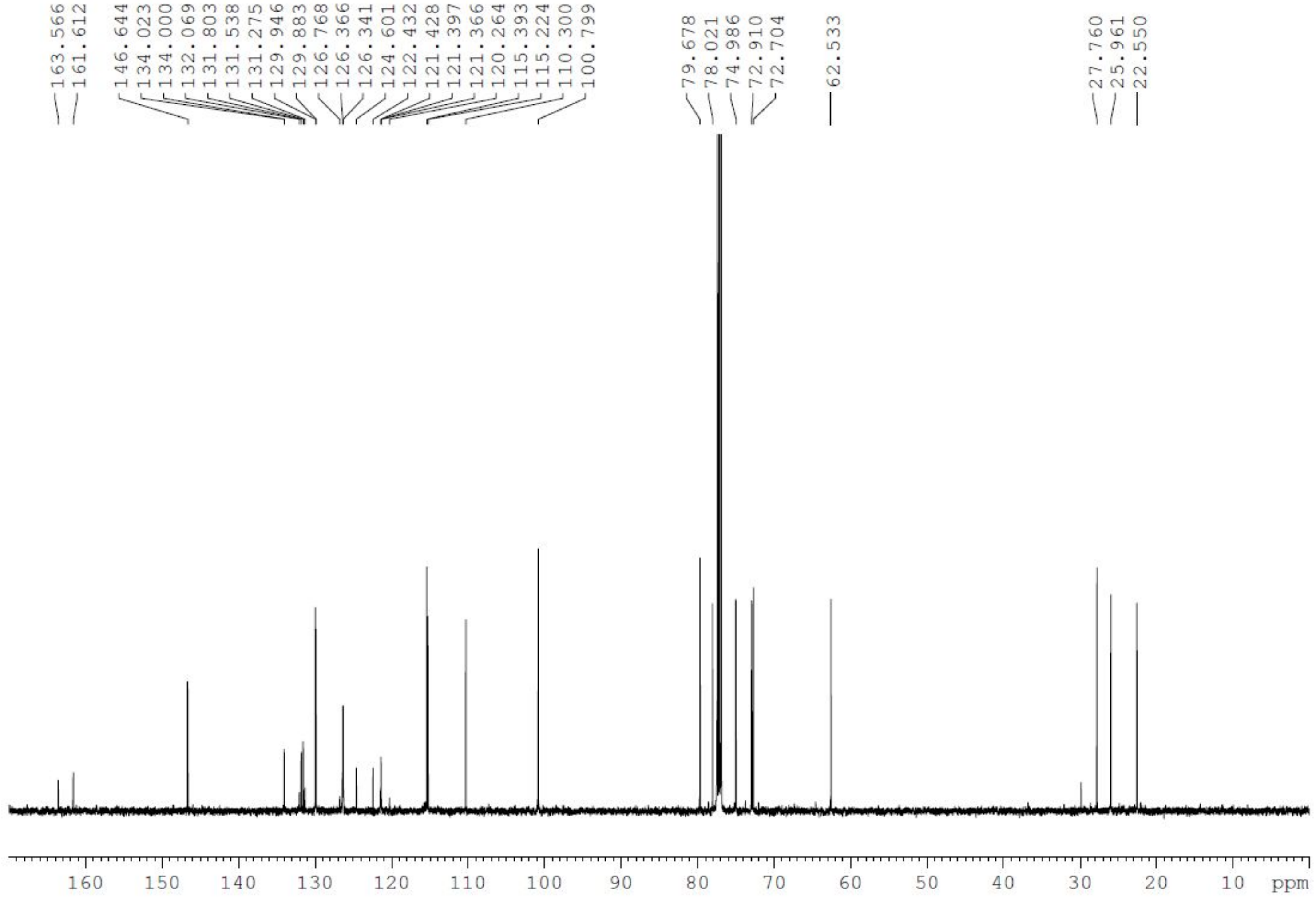

(*R*)-{1-[3,5-bis-(trifluoromethyl)phenyl]ethyl} 2-*O*-*p*-fluorobenzyl-3,4-*O*-isopropylidene-β-L-arabinopyranoside, (**22β**)

^{1}H-NMR (500 MHz, $CDCl_3$)

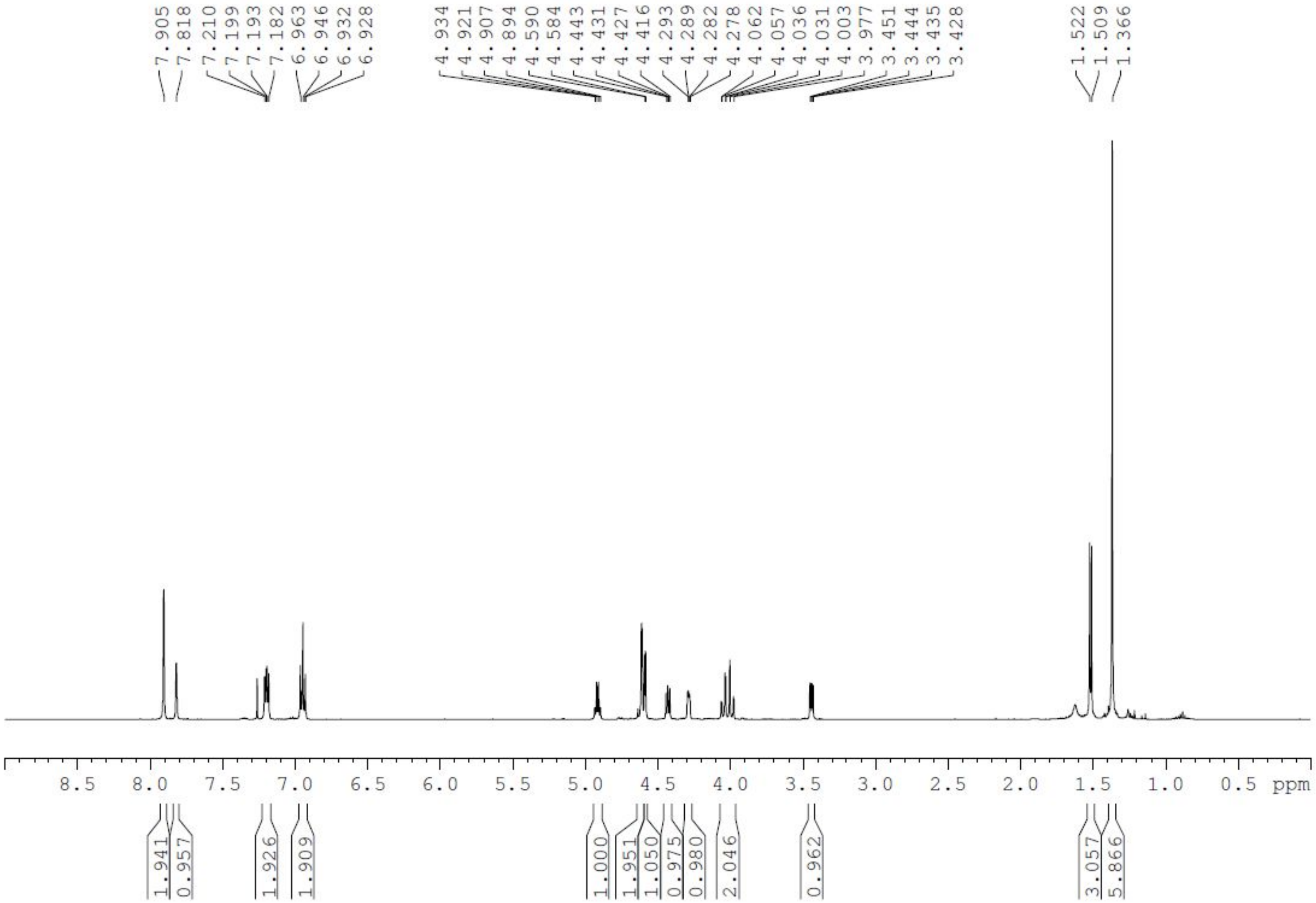

(*R*)-{1-[3,5-bis-(trifluoromethyl)phenyl]ethyl} 2-*O*-*p*-fluorobenzyl-3,4-*O*-isopropylidene-β-L-arabinopyranoside, (**22**β)

^{13}C-NMR (125 MHz, $CDCl_3$)

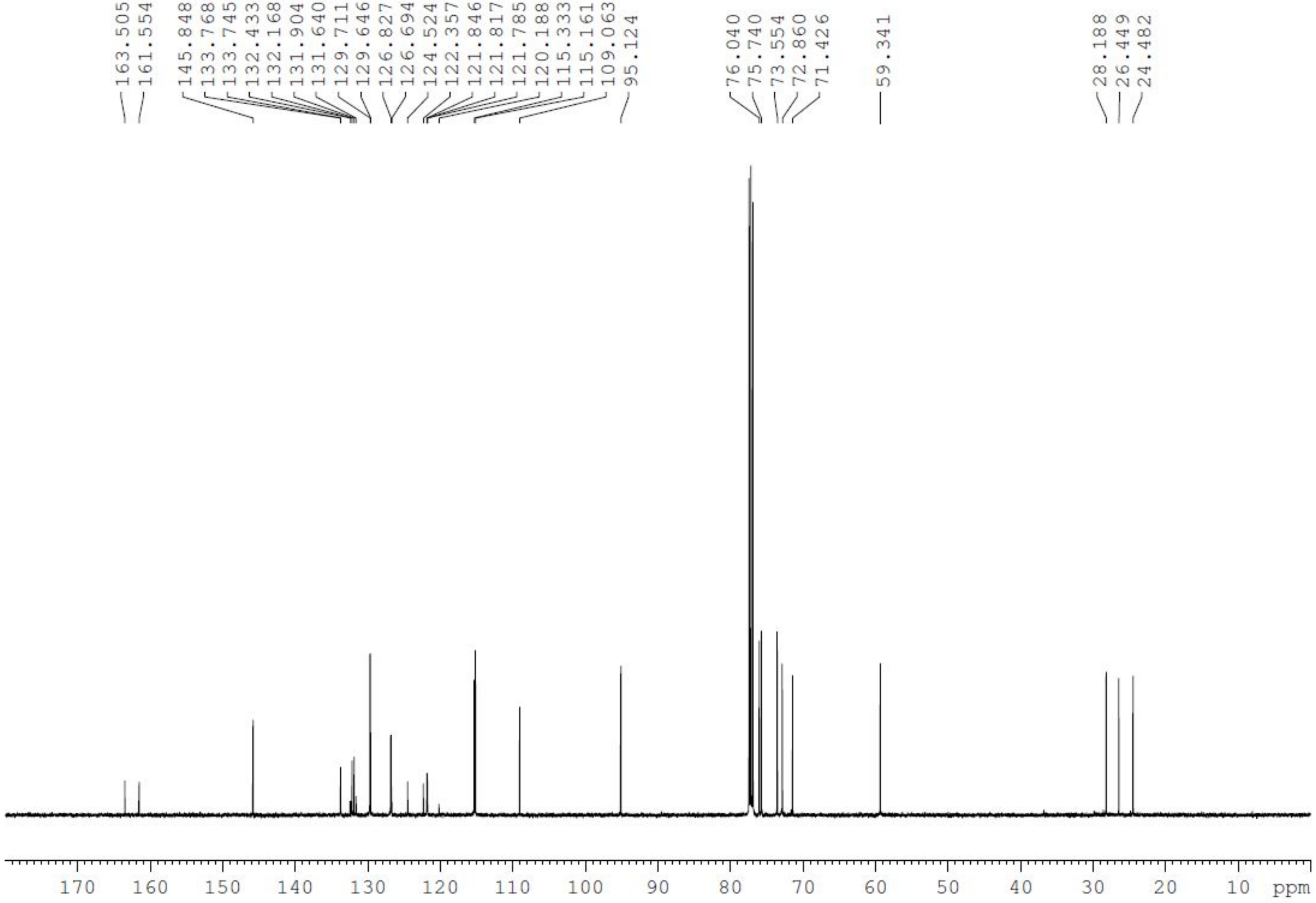

(*R*)-{1-[3,5-bis-(trifluoromethyl)phenyl] ethyl} 2-*O*-*p*-fluorobenzyl-α-L-arabinopyranoside, (**23α**)

^{1}H-NMR (500 MHz, $CDCl_3$)

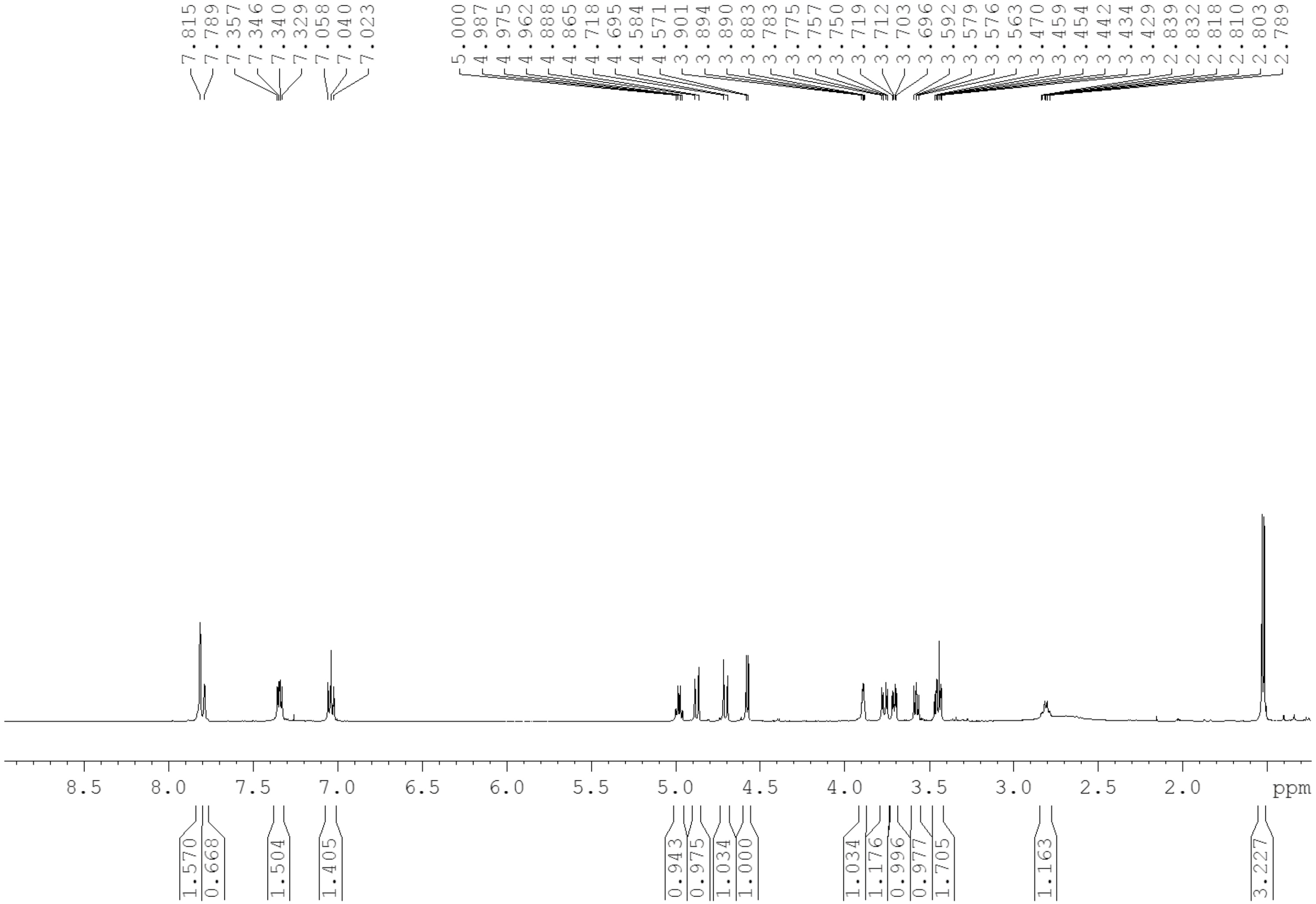

(*R*)-{1-[3,5-bis-(trifluoromethyl)phenyl] ethyl} 2-*O*-*p*-fluorobenzyl-α-L-arabinopyranoside, (**23α**)

^{13}C-NMR (125 MHz, $CDCl_3$)

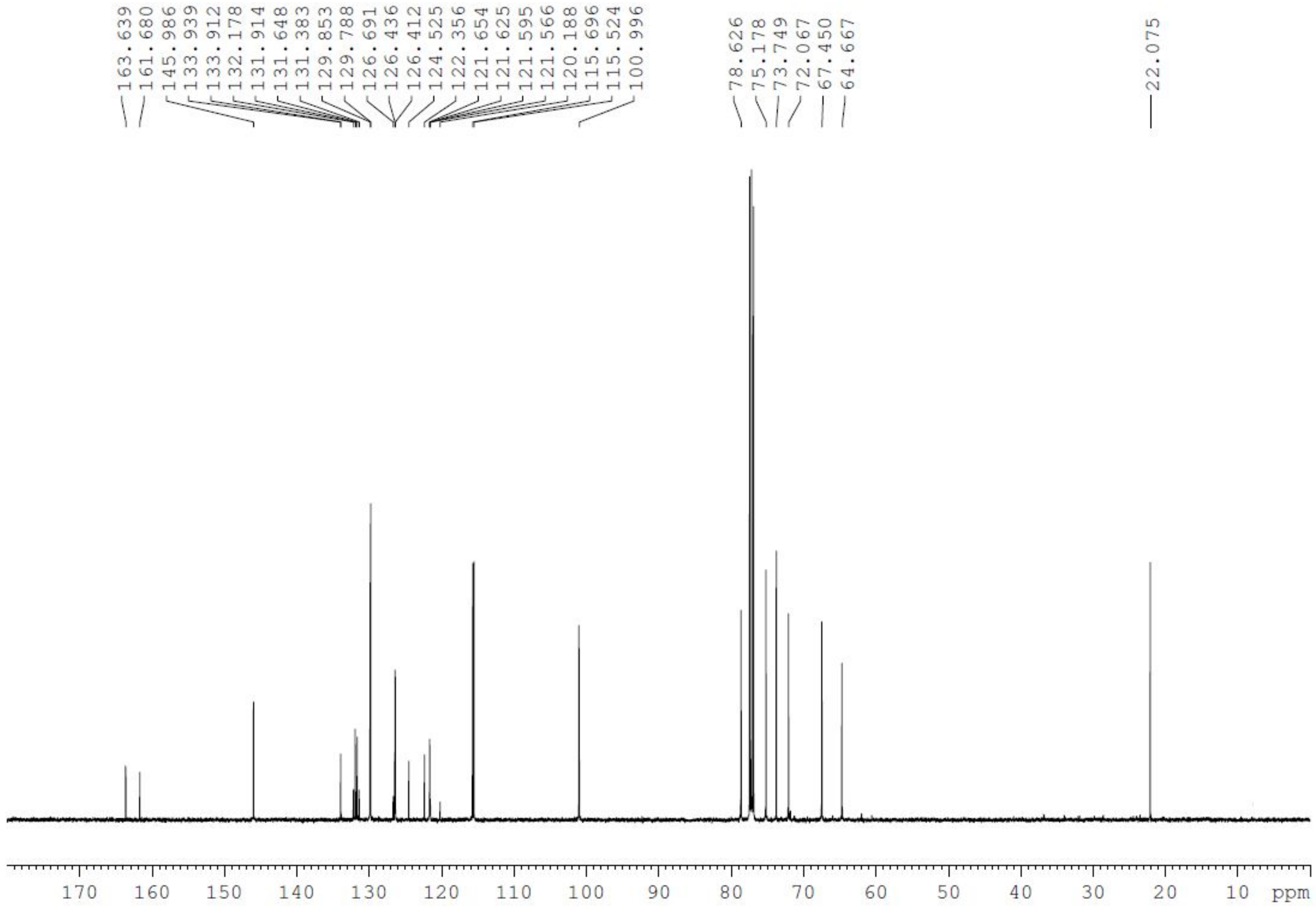

(*R*)-{1-[3,5-bis-(trifluoromethyl)phenyl]ethyl} 2-*O*-*p*-fluorobenzyl-β-L-arabinopyranoside, (**23β**)

^{1}H-NMR (500 MHz, $CDCl_3$)

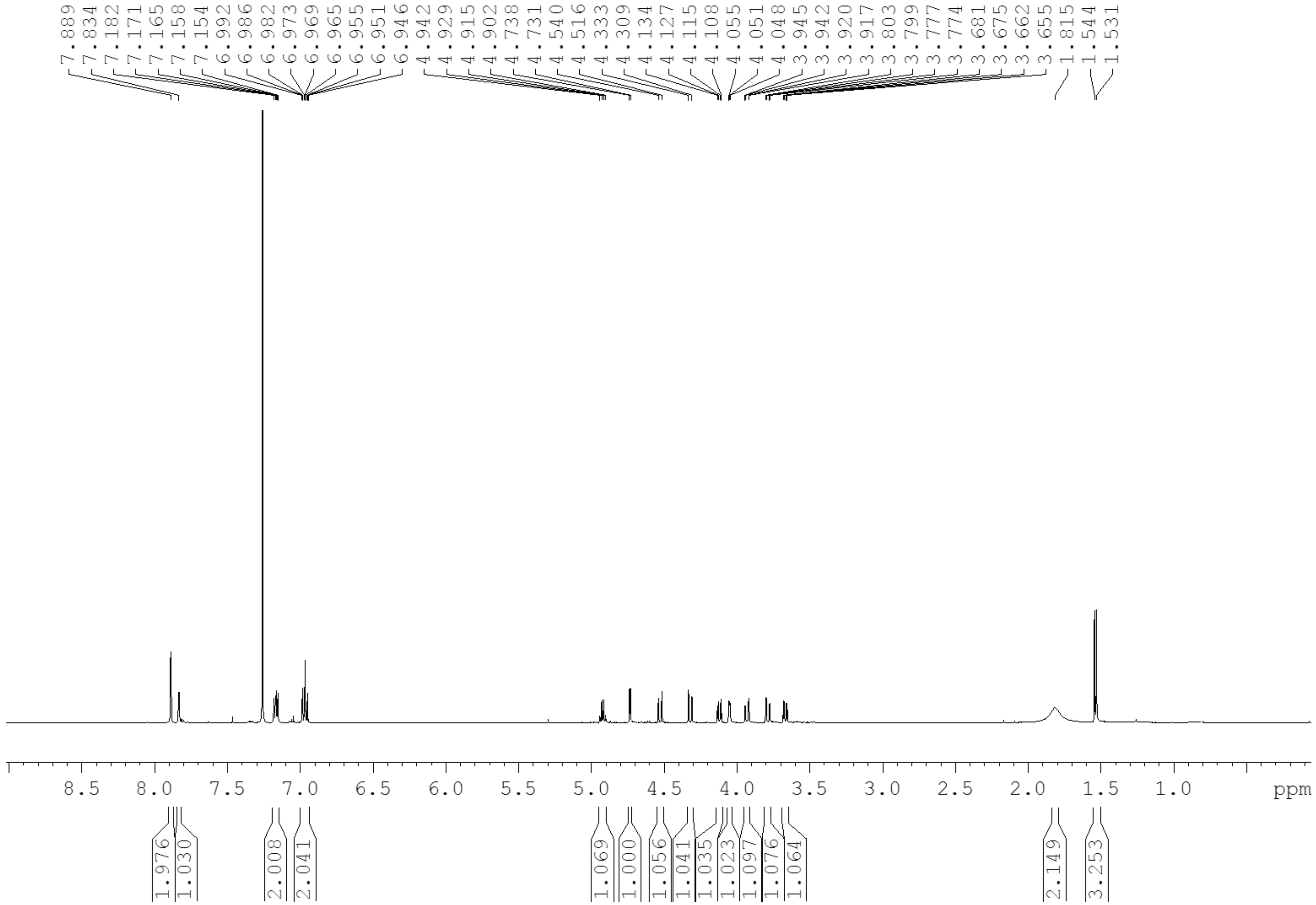

(*R*)-{1-[3,5-bis-(trifluoromethyl)phenyl]ethyl} 2-*O*-*p*-fluorobenzyl-β-L-arabinopyranoside, (**23β**)

^{13}C-NMR (125 MHz, $CDCl_3$)

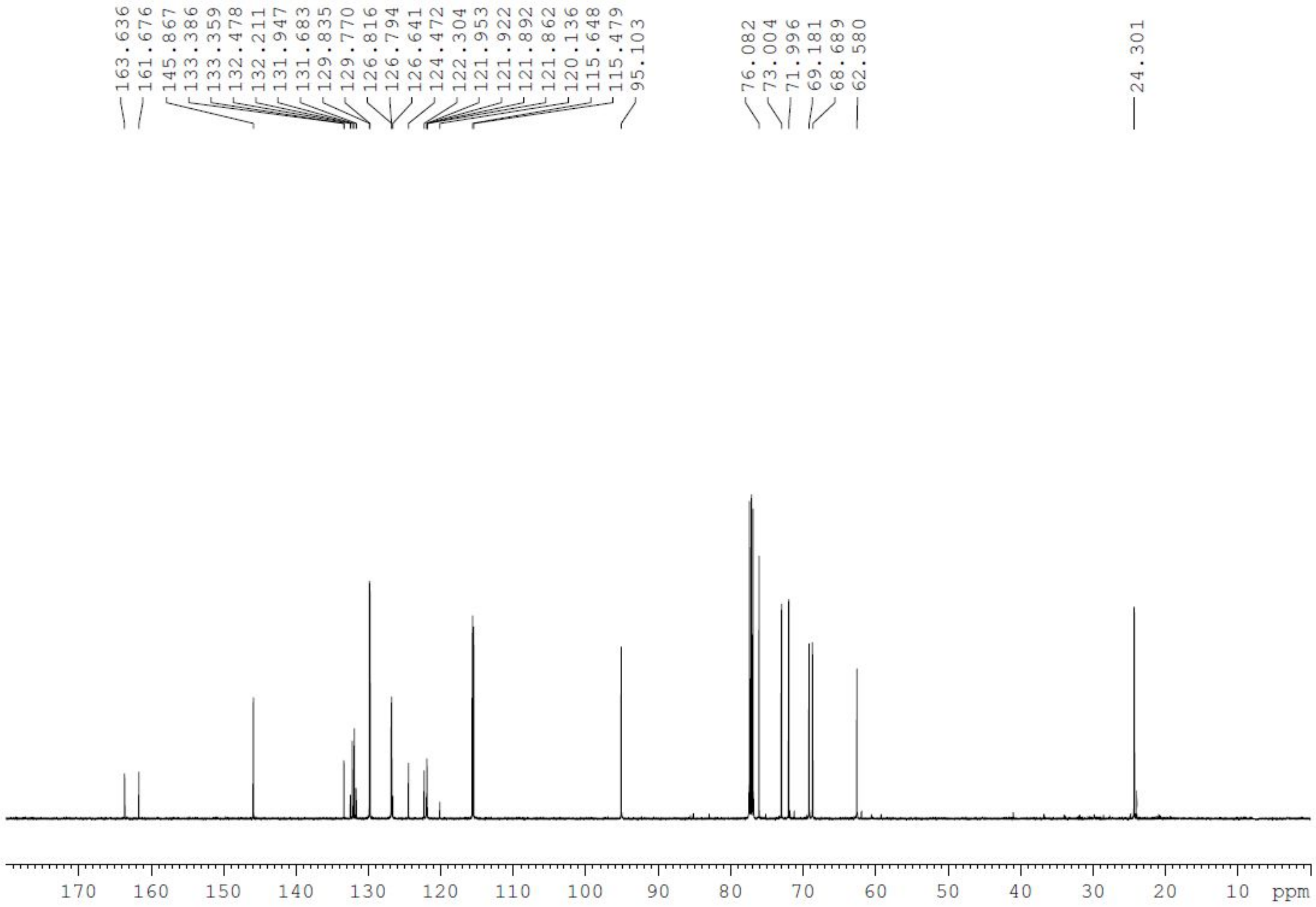

NOE studies of 14α and 14β

(*R*)-{1-[3,5-bis-(trifluoromethyl)phenyl]ethyl} (*R*)-(4,6-*O*-benzylidene)-2-*O*-*p*-fluorobenzyl-α-D-galactopyranoside, (14α)

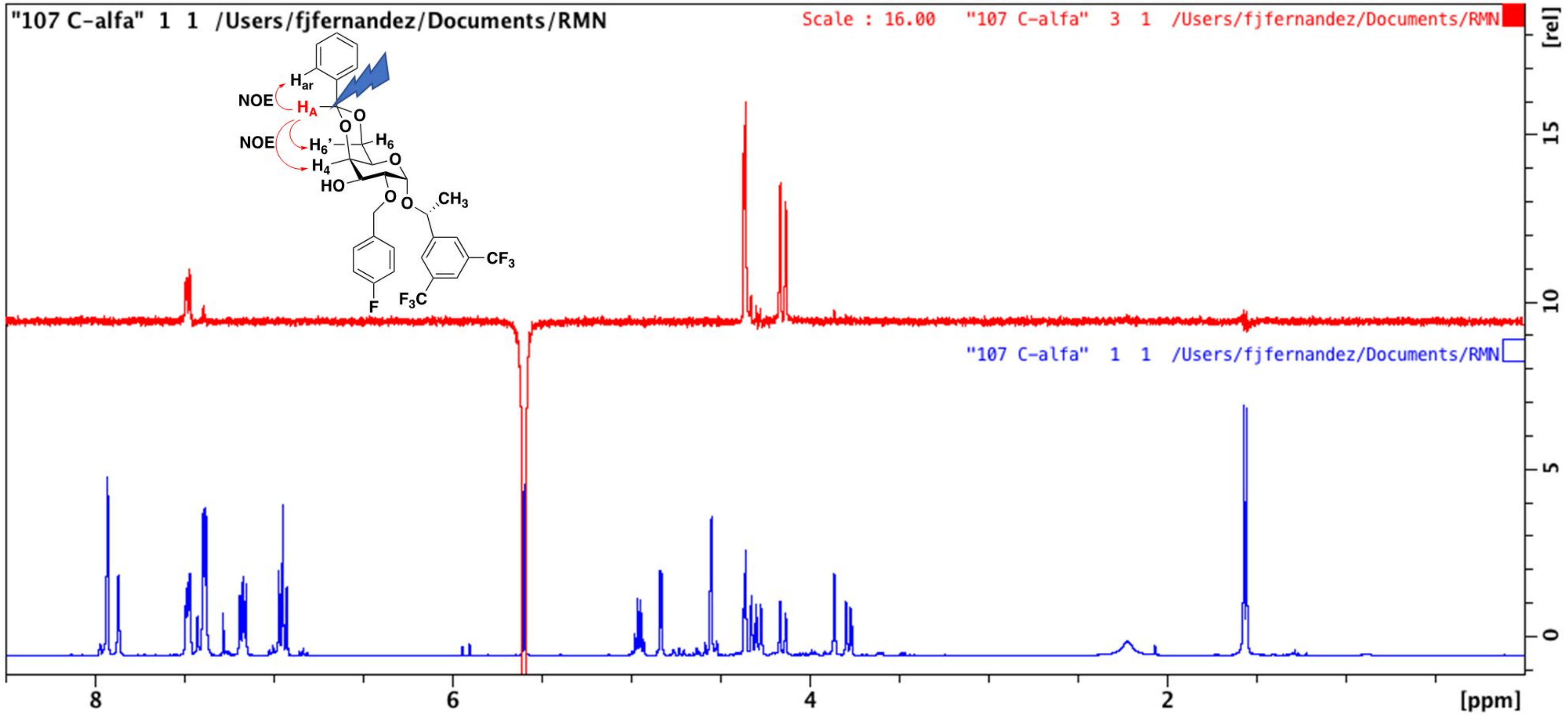

(*R*)-{1-[3,5-bis-(trifluoromethyl)phenyl]ethyl} (*R*)-(4,6-*O*-benzylidene)- 2-*O*-*p*-fluorobenzyl-β-D-galactopyranoside, (14β)

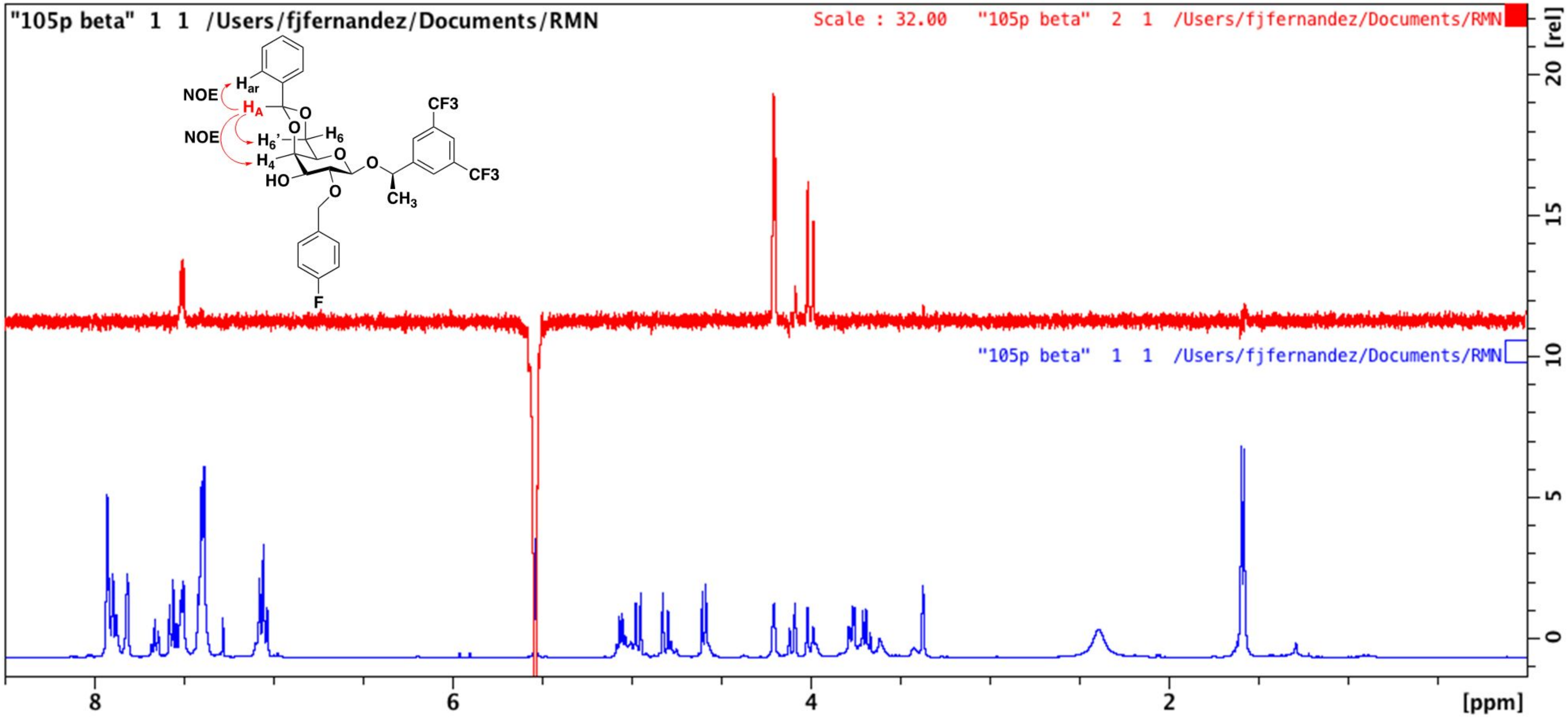

Molecular modelling

Docking validation

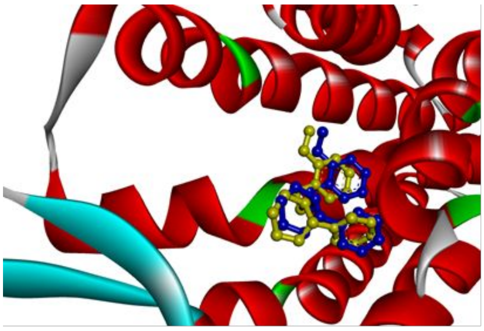

CP-99,994

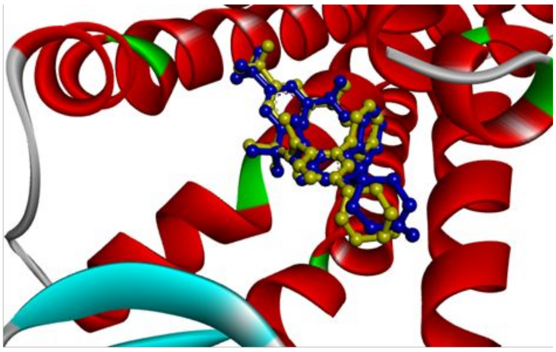

Netupitant

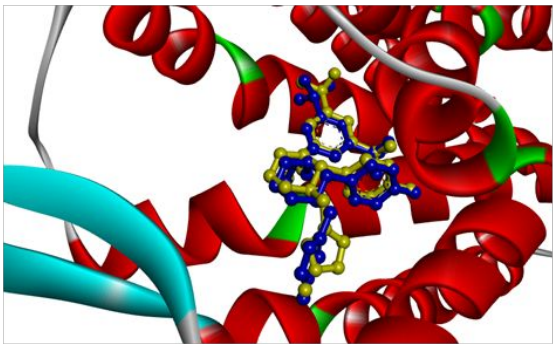

Aprepitant

Figure S-1. Docking validation, a comparison of the docked binding modes (blue) and the co-crystallized poses (yellow) of some NK1R antagonists: CP-99,994 (PDB ID: 6HLL), Netupitant (PDB ID: 6HLP) and Aprepitant (PBD ID: 6HLO). For clarity, hydrogen atoms have been removed.

Biological evaluation

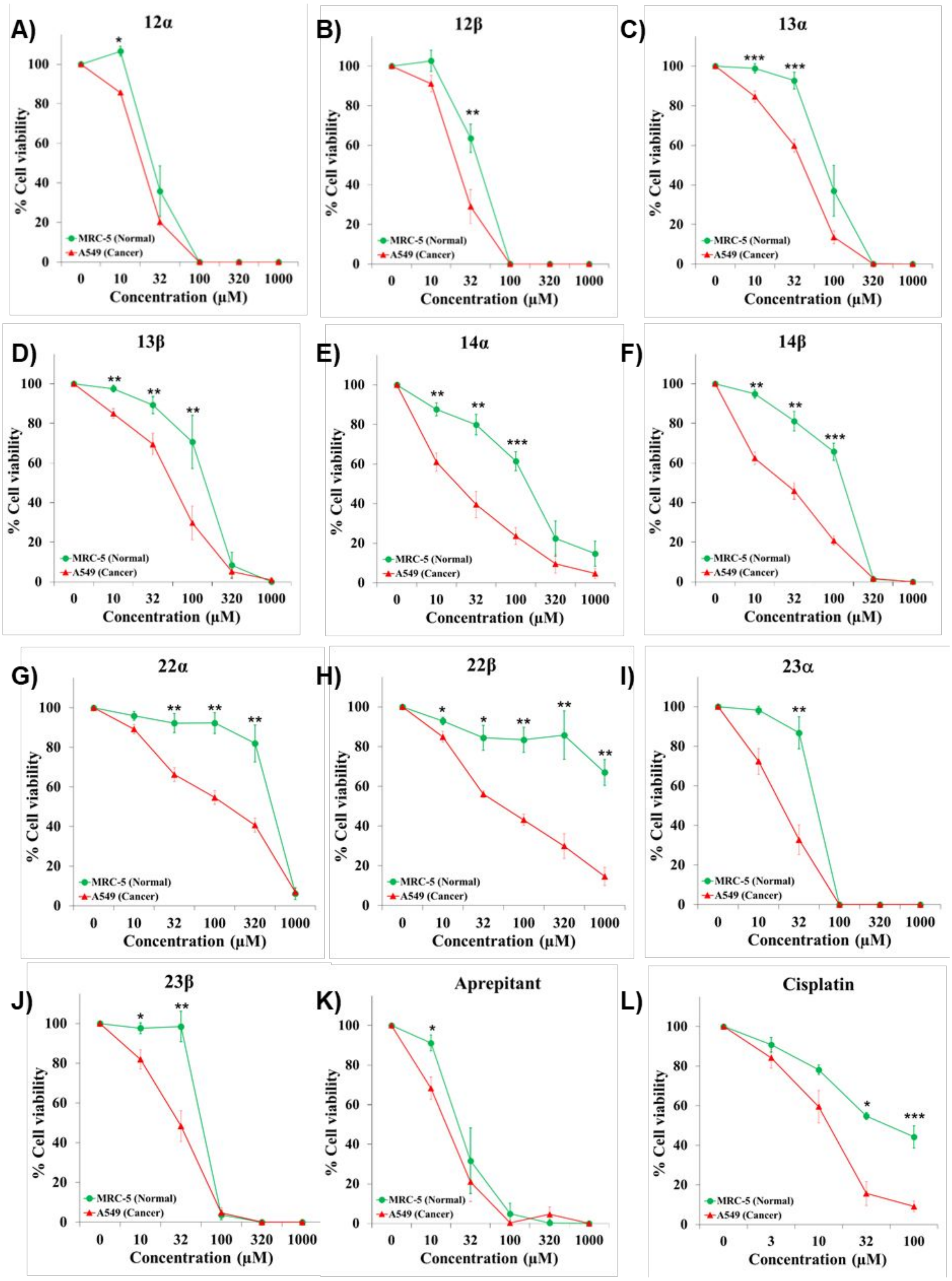

Figure S-2. Evaluation of cytotoxic activity of D-galacto and L-arabino derivatives, Aprepitant and Cisplatin on A549 lung cancer cells and MRC-5 lung normal cells. Cells were exposed to several concentrations of compounds for 48 h. Cell viability was deter-mined with the MTT assay. Results are representative of at least three independent experiments. Values are expressed in mean with SEM and p values (paired t-test) indicated.

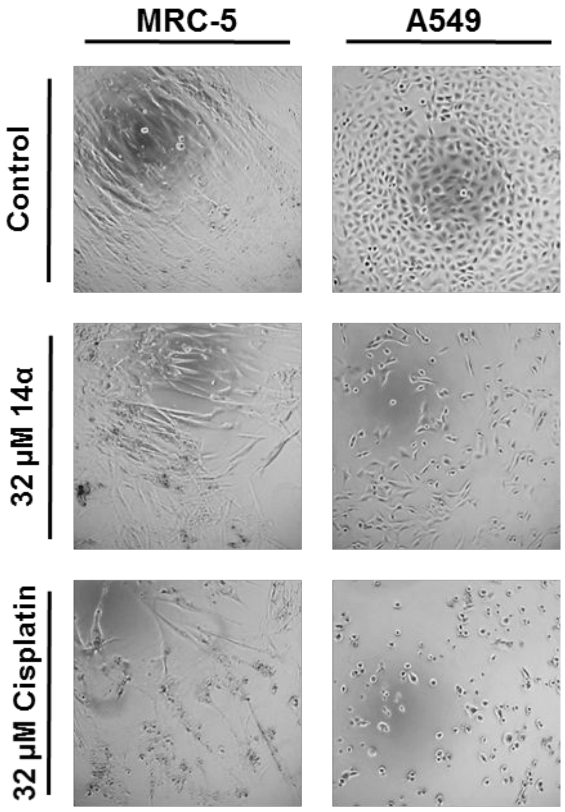

Figure S-3. Representative photographs of lung cancer cells (A549) and lung normal cells (MRC-5) untreated (control) and treated with **14α** (32 µM) or Cisplatin (32 µM) for 48 h.

Competition Experiments of Cytotoxic effect of 14α in presence of SP.

First, we treated A549 cells with several concentrations of SP (2.5 - 200 nM) for 48 h and we evaluated cell proliferation with the Resazurin assay. Although it has been reported that A549 cells overexpress NK1R,[1] we did not observe any increase in cell proliferation after the addition of SP. The highest SP concentration tested (200 nM) was added 1 h before 32 µM of **14α** or Cisplatin. After a 48 h treatment period, the cell viability was determined with the Resazurin assay. Results are indicated in Figure 4.

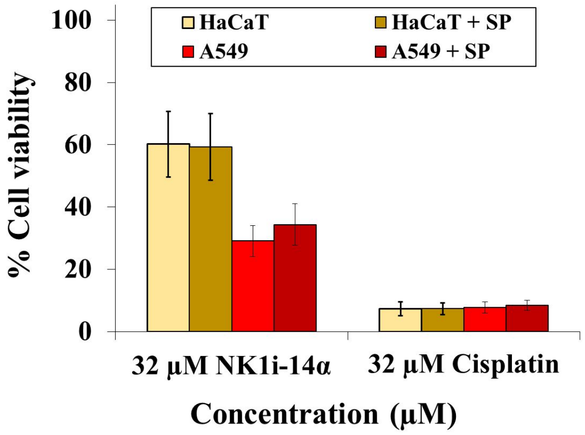

Figure S-4. Evaluation of cytotoxic activity of **14α** derivative and Cisplatin on A549 lung cancer cells and HaCaT normal cells in presence of SP. Results are representative of at least three independent experiments. Values are expressed in mean with SEM.

[1] O'Connor, T.M.; O'Connell, J.; O'Brien, D.I.; Bennett, M.W.; Goode, T.; Burke, L.; Bredin, C.P.; Shanahan, F. *J. Clin. Immunol.* **2003**, *23*(5), 425-35.

HPLC traces for lead compounds

Purity of the compounds was confirmed by HPLC, using an ACE Excel C18 2μm 4.6 x 100 mm column. The gradient elution and flux for all the compounds was from 70% acetonitrile/30% water to 100% acetonitrile/0% water with 0.1% formic acid in 7 min. and 1mL/min, respectively. Except for compound **22β**, where a gradient elution from 80% acetonitrile/20% water to 100% acetonitrile/0% water with 0.1% formic acid in 7 min. was used.

(*R*)-{1-[3,5-bis-(trifluoromethyl)phenyl]ethyl} 2-*O*-*p*-fluorobenzyl-3,4-*O*-isopropylidene-α-D-galactopyranoside, (**12α**)

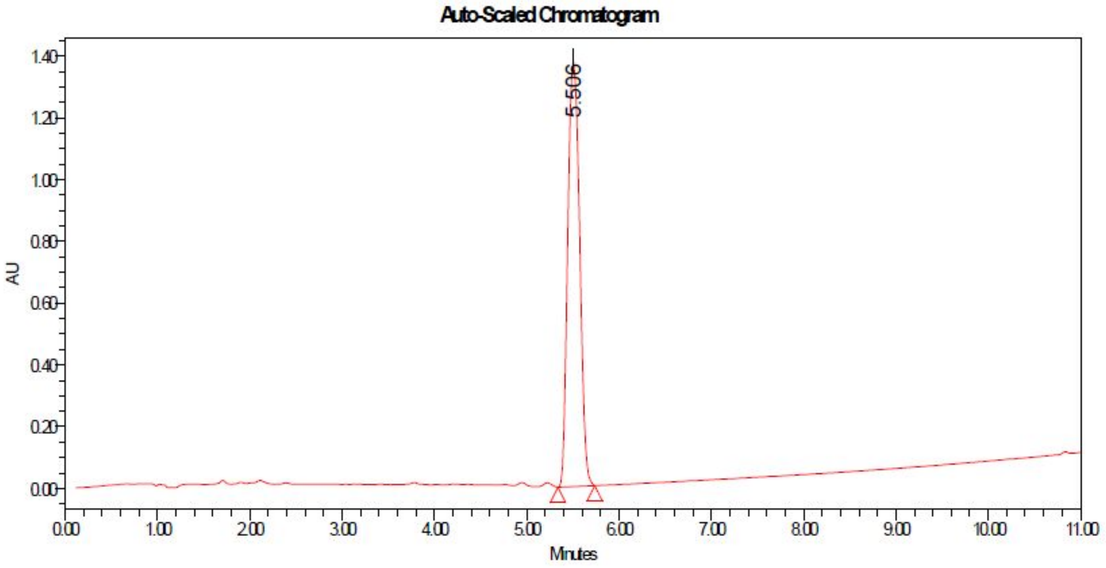

Peak Results

	RT	Area	% Area	Height (μV)
1	5.506	12212256	100.00	1375805

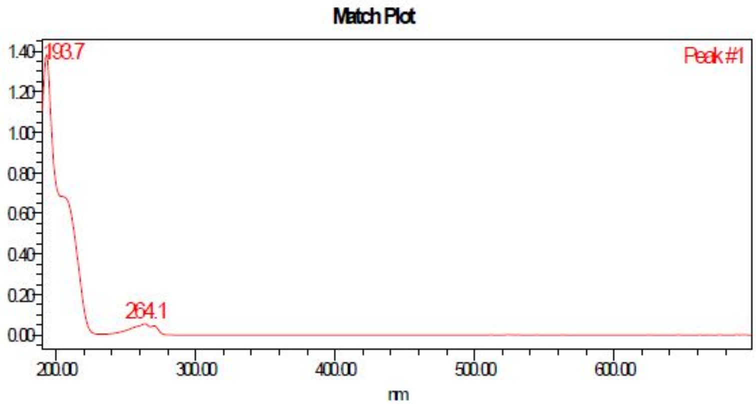

(*R*)-{1-[3,5-bis-(trifluoromethyl)phenyl]ethyl} 2-*O*-*p*-fluorobenzyl-3,4-*O*-isopropylidene-α-D-galactopyranoside, (**12β**)

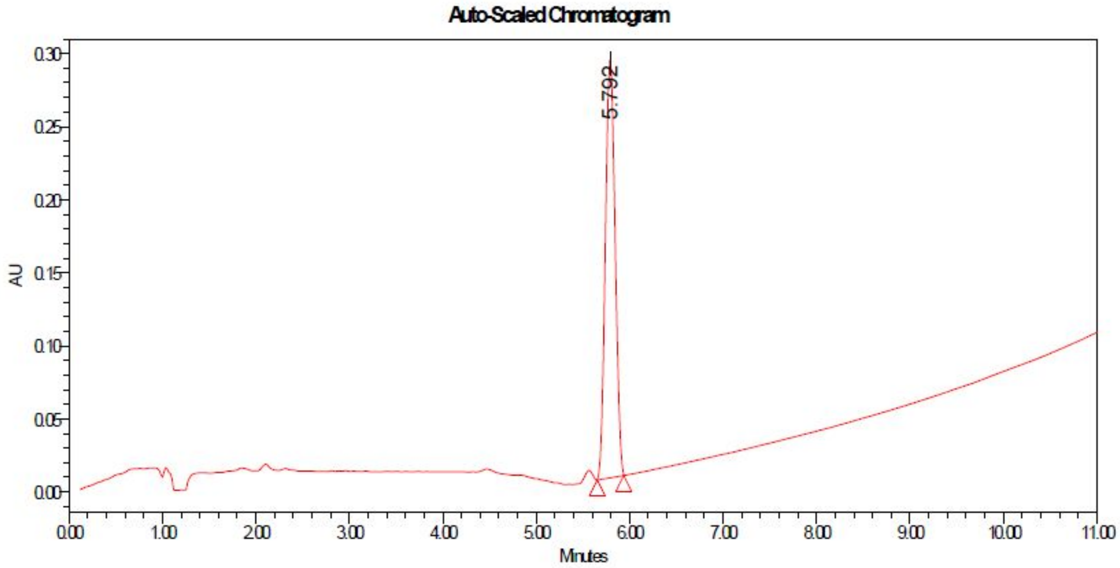

Peak Results

	RT	Area	% Area	Height (µV)
1	5.792	2062082	100.00	286277

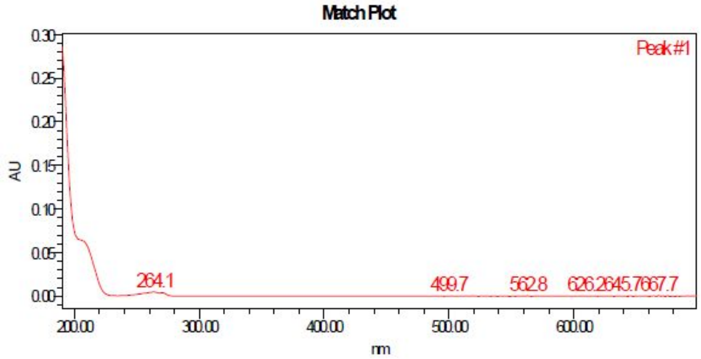

(*R*)-{1-[3,5-bis-(trifluoromethyl)phenyl]ethyl} 2-*O*-*p*-fluorobenzyl-α-D-galactopyranoside, (13α)

Auto-Scaled Chromatogram

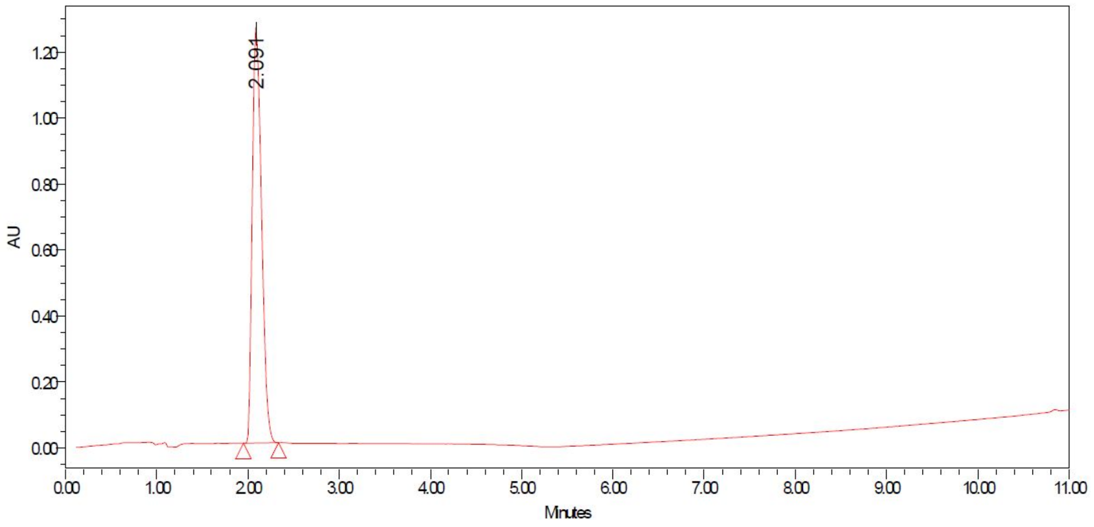

Peak Results

	RT	Area	% Area	Height (µV)
1	2.091	8852948	100.00	1262892

Match Plot

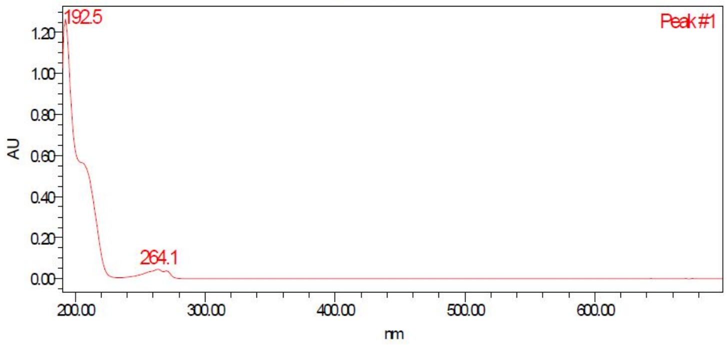

(*R*)-{1-[3,5-bis-(trifluoromethyl)phenyl]ethyl} 2-*O*-*p*-fluorobenzyl-α-D-galactopyranoside, (13β)

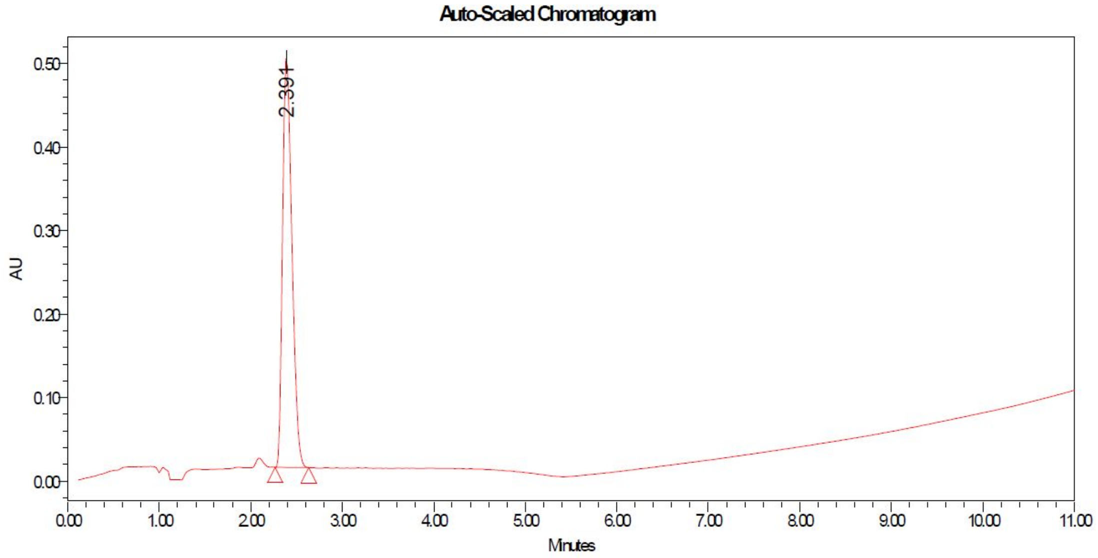

Peak Results

	RT	Area	% Area	Height (μV)
1	2.391	3474428	100.00	493870

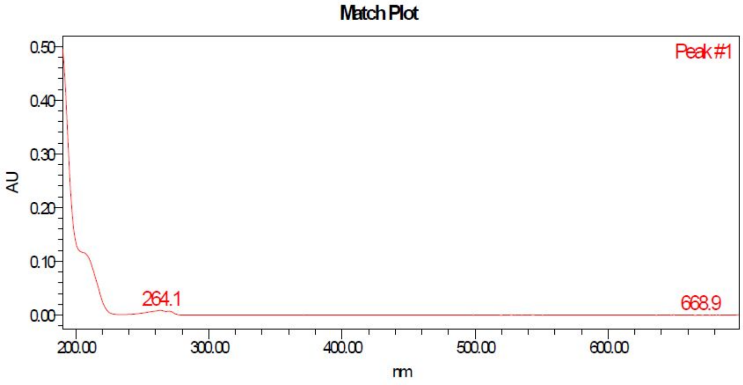

(*R*)-{1-[3,5-bis-(trifluoromethyl)phenyl]ethyl} (*R*)-(4,6-*O*-benzylidene)-2-*O*-*p*-fluorobenzyl-α-D-galactopyranoside, (14α)

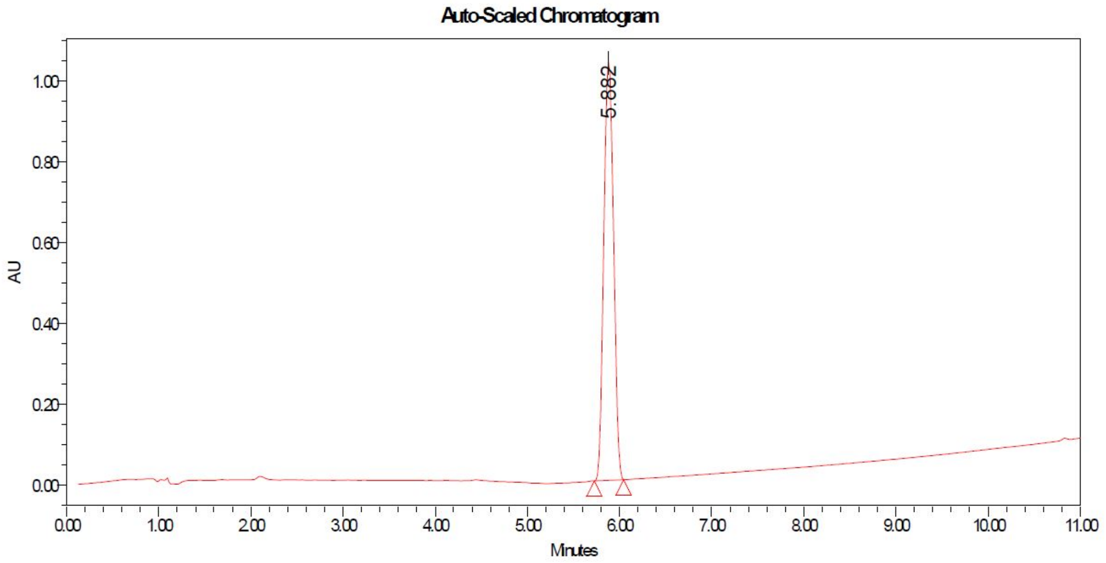

Peak Results

	RT	Area	% Area	Height (µV)
1	5.882	7609394	100.00	1039024

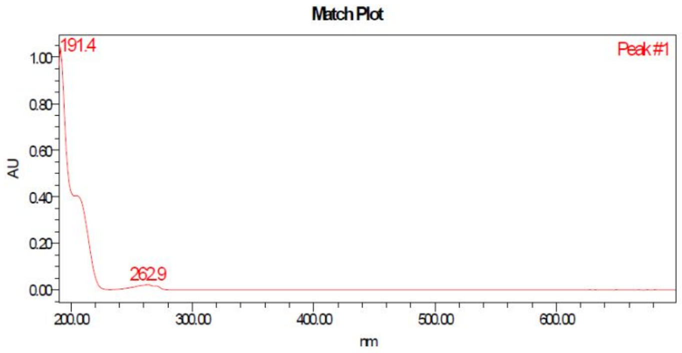

(*R*)-{1-[3,5-bis-(trifluoromethyl)phenyl]ethyl} (*R*)-(4,6-*O*-benzylidene)-2-*O*-*p*-fluorobenzyl-α-D-galactopyranoside, (14β)

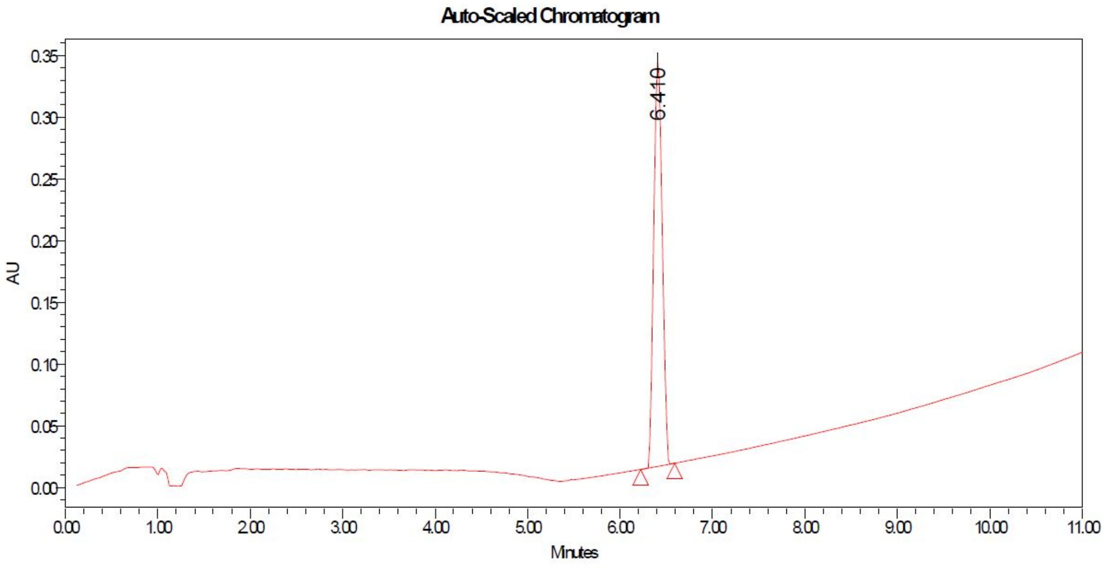

Peak Results

	RT	Area	% Area	Height (μV)
1	6.410	2095352	100.00	327885

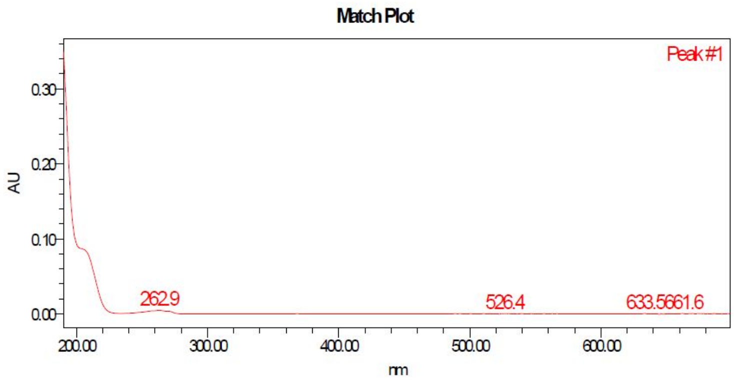

(*R*)-{1-[3,5-bis-(trifluoromethyl)phenyl]ethyl} 2-*O*-*p*-fluorobenzyl-3,4-*O*-isopropylidene-β-L-arabinopyranoside, (**22α**)

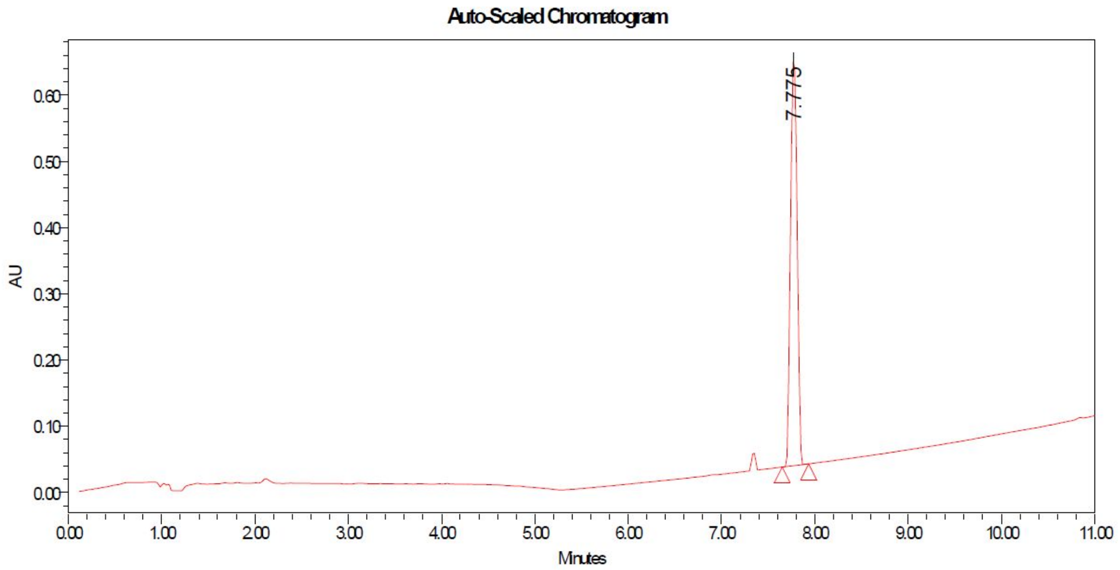

Peak Results

	RT	Area	% Area	Height (μV)
1	7.775	3037387	100.00	612799

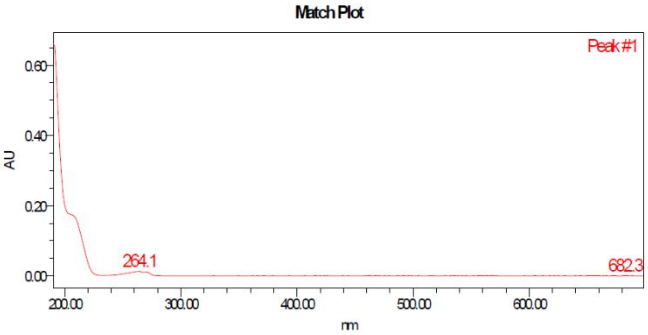

(*R*)-{1-[3,5-bis-(trifluoromethyl)phenyl]ethyl} 2-*O*-*p*-fluorobenzyl-3,4-*O*-isopropylidene-β-L-arabinopyranoside, (**22β**)

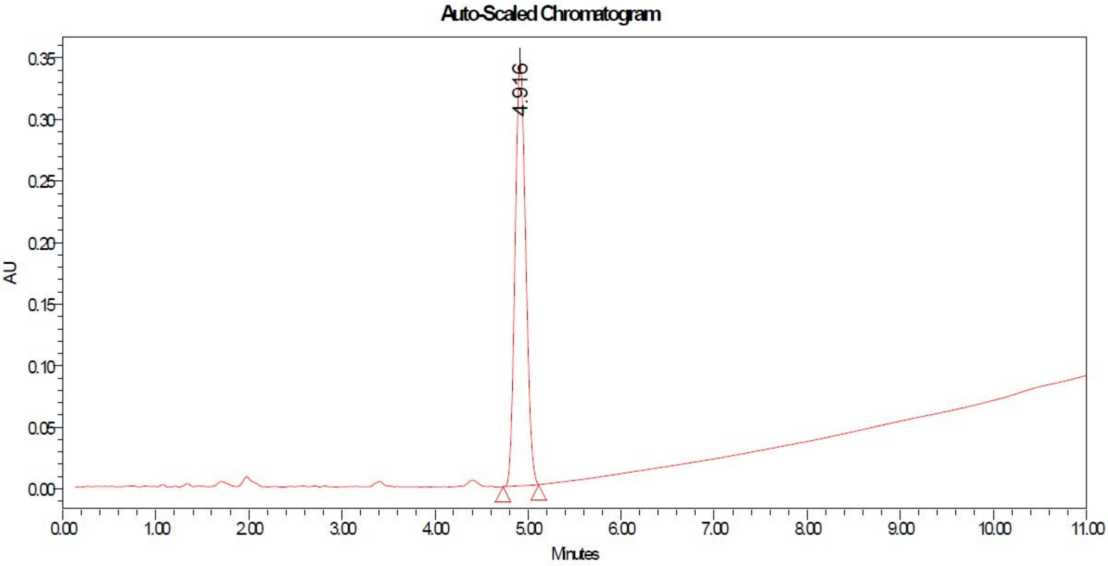

Peak Results

	RT	Area	% Area	Height (µV)
1	4.916	2729001	100.00	345888

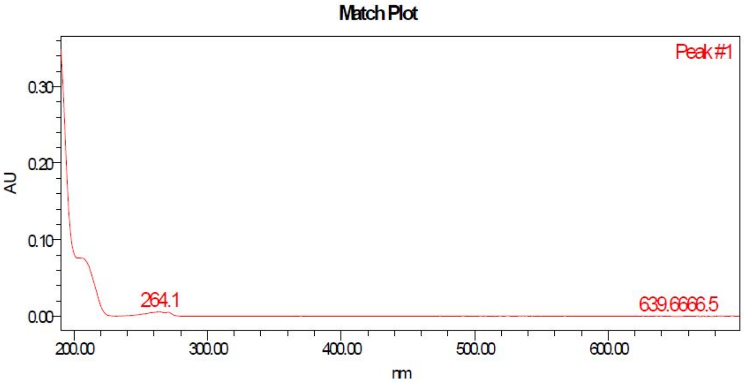

(*R*)-{1-[3,5-bis-(trifluoromethyl)phenyl]ethyl} 2-*O*-*p*-fluorobenzyl-β-L-arabinopyranoside, (23α)

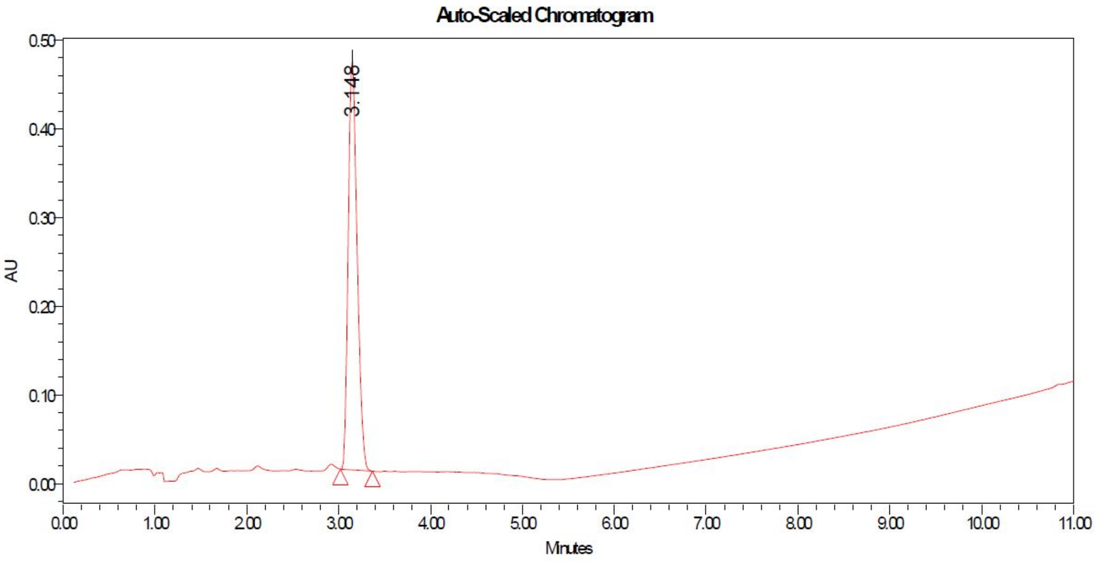

Peak Results

	RT	Area	% Area	Height (μV)
1	3.148	3142313	100.00	461923

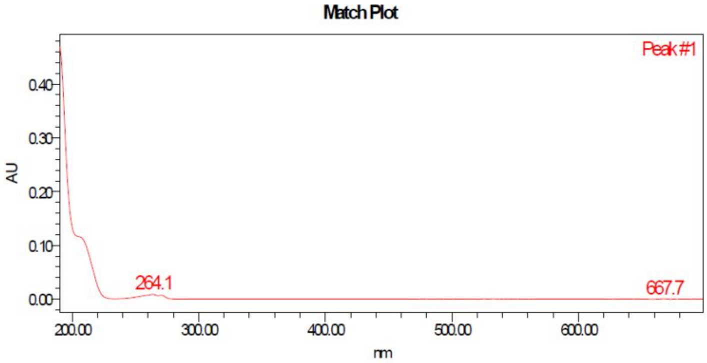

(*R*)-{1-[3,5-bis-(trifluoromethyl)phenyl]ethyl} 2-*O*-*p*-fluorobenzyl-β-L-arabinopyranoside, (23β)

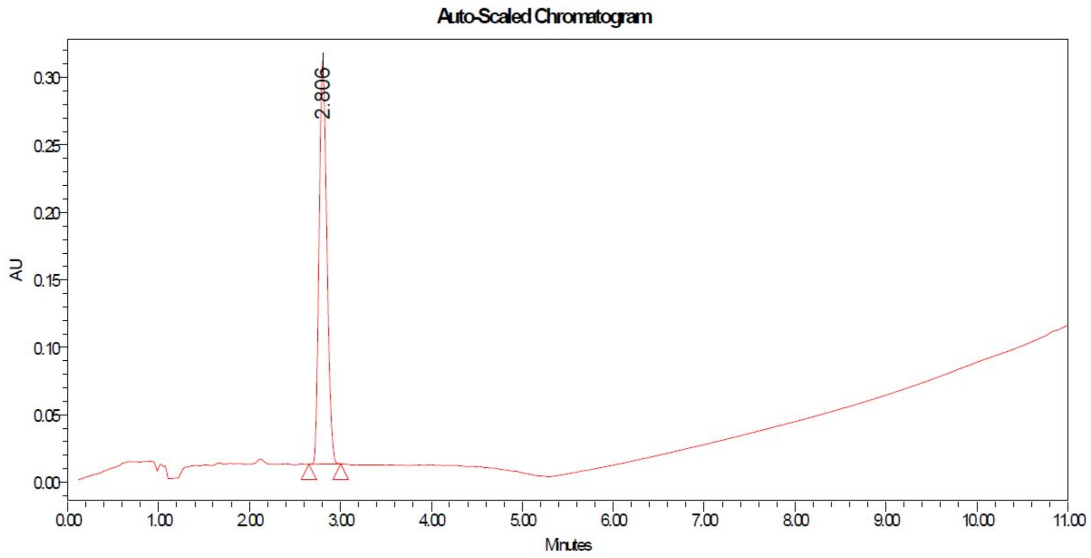

Peak Results

	RT	Area	% Area	Height (μV)
1	2.806	1793617	100.00	297639

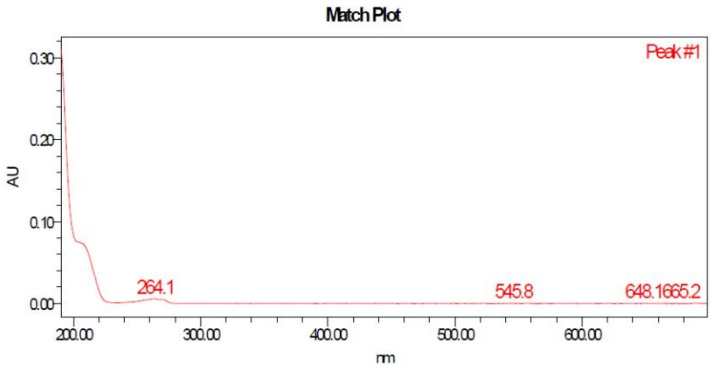

Supplement: Supplementary file 1 — jm1c00793_si_001.pdf [file jm1c00793_si_001.pdf]
